# Effectiveness of Later-Stage Exercise Programs vs Usual Medical Care on Physical Function and Activity After Total Knee Replacement: A Randomized Clinical Trial

**DOI:** 10.1001/jamanetworkopen.2019.0018

**Published:** 2019-02-22

**Authors:** Sara R. Piva, Michael J. Schneider, Charity G. Moore, M. Beatriz Catelani, Alexandra B. Gil, Brian A. Klatt, Anthony M. DiGioia, Gustavo J. Almeida, Samannaaz S. Khoja, Gwendolyn Sowa, James J. Irrgang

**Affiliations:** 1 Department of Physical Therapy, University of Pittsburgh, Pittsburgh, Pennsylvania; 2 Department of Orthopaedic Surgery, University of Pittsburgh, Pittsburgh, Pennsylvania; 3 Bone and Joint Center at UPMC Magee-Womens Hospital, University of Pittsburgh Medical Center, Pittsburgh, Pennsylvania; 4 Department of Physical Medicine & Rehabilitation, University of Pittsburgh, Pittsburgh, Pennsylvania

## Abstract

**Importance:**

Several functional limitations persist after total knee replacement (TKR). Intensive exercise programs could resolve these limitations but are not well tolerated by many patients until a later stage (>2 months) after surgery. Evidence for exercise at a later stage after TKR is limited.

**Objectives:**

To compare the effectiveness of later-stage exercise programs after TKR and to explore heterogeneity of treatment effects.

**Design, Setting, and Participants:**

Three-arm single-blind randomized clinical trial (January 7, 2015, to November 9, 2017) using an intent-to-treat approach with follow-ups at 3 months and 6 months. The setting was Allegheny County, Pennsylvania (an outpatient physical therapy clinic and 4 community centers). Participants had primary TKR performed more than 2 months previously, were 60 years or older, experienced moderate functional limitations, and were medically cleared to exercise.

**Interventions:**

Clinic-based physical therapy exercise (physical therapy arm), community-based group exercise (community arm), and usual care (control arm). The control arm continued their usual care, whereas the exercise arms participated in supervised exercise programs lasting 12 weeks.

**Main Outcomes and Measures:**

The primary outcome was arm differences in the Western Ontario and McMaster Universities Osteoarthritis Index–Physical Function (WOMAC-PF) at 3 months. The secondary outcomes included performance-based tests germane to knee replacement and additional surveys of physical function. Data were analyzed by linear mixed models and responder analysis.

**Results:**

A total of 240 participants (mean [SD] age, 70 [7] years; 61.7% female) were allocated to physical therapy (n = 96), community exercise (n = 96), or control (n = 48). All 3 arms demonstrated clinically important improvement. At 3 months, between-arm analyses for the WOMAC-PF demonstrated no differences between physical therapy and community (−2.2; 98.3% CI, −4.5 to 0.1), physical therapy and control (−2.1; 98.3% CI, −4.9 to 0.7), and community and control (0.1; 98.3% CI, −2.7 to 2.9). Performance-based tests demonstrated greater improvement in the physical therapy arm compared with both the community (0.1 *z* score units; 98.3% CI, 0.0-0.2) and control (0.3 *z* score units; 98.3% CI, 0.1-0.4) arms and the community arm compared with the control arm (0.2 *z* score units; 98.3% CI, 0.0-0.3). The physical therapy arm had more than 17.7% responders than the community arm and more than 19.0% responders than the control arm. There was no difference in responder rates between the community and control arms.

**Conclusions and Relevance:**

Based on the primary outcome, participation in late-stage exercise programs after TKR offered no benefit over usual care. The benefits of physical therapy identified by the secondary outcomes and responder analysis require confirmation.

**Trial Registration:**

ClinicalTrials.gov Identifier: NCT02237911

## Key Points

**Question:**

Can exercise programs delivered at a later stage (>2 months) after total knee replacement improve the functional limitations that persist after surgery?

**Findings:**

In this randomized clinical trial of 240 individuals at a later stage after knee replacement, all arms (physical therapy, community, and control) improved physical function. There were no differences between arms in the primary outcome of patient-reported physical function, whereas the secondary outcome of performance-based physical function demonstrated greater improvement in the physical therapy arm.

**Meaning:**

While the primary outcome suggests no benefit of later-stage exercise programs, the secondary outcomes suggest beneficial effects of physical therapy but require confirmation.

## Teaser

This randomized clinical trial compares the effectiveness of later-stage exercise programs of clinic-based physical therapy exercise or community-based group exercise vs usual care after total knee replacement and explores heterogeneity of treatment effects.

## Introduction

Total knee replacements (TKRs) are highly prevalent, with more than 4 million US adults living with a TKR, and by 2030 greater than 3 million are projected annually.^1^ Although TKRs are successful at reducing pain and improving quality of life,^2^ many of the long-term mobility limitations due to the chronic joint disease that existed for decades before surgery do not spontaneously resolve after TKR.^2,3,4,5^ Exercise therapy is a simple solution for alleviating these persistent mobility limitations and enhancing TKR outcomes.

Current rehabilitation care in TKR typically consists of discharge from supervised exercise within 2 to 3 months after surgery.^6,7^ However, during these first few months (early stage), patients after TKR are still healing from the surgical insult, and exercises cannot be performed with sufficient intensity to reduce the mobility limitations. To succeed in reversing long-lasting mobility limitations, exercise programs should specifically address mobility deficits germane to patients after TKR and be sufficiently dosed (ie, duration, frequency, and intensity) to promote adaptive responses, which may not be tolerated by most patients until the later stage of rehabilitation (>2 months) after TKR.

Clinical guidelines from the American Academy of Orthopaedic Surgeons conclude that there is limited evidence to recommend supervised exercise during later-stage rehabilitation after TKR and called for additional research.^8^ We designed a study to inform patients and clinicians about the benefits of exercise programs for the later stage after TKR and to provide evidence to tailor interventions according to patient characteristics. The study compared the outcomes of physical function, activity, and safety and explored heterogeneity of treatment effects.

## Methods

### Study Design and Oversight

This study was a 3-arm single-blind randomized clinical trial conducted in an outpatient physical therapy clinic (the Physical Therapy Clinical and Translational Research Center at the University of Pittsburgh) and 4 community centers in Allegheny County, Pennsylvania. Participants signed an informed consent document reviewed and approved by the University of Pittsburgh Institutional Review Board, which also approved the study protocols. The study was monitored by an independent data and safety monitoring board and an advisory panel of patients and clinicians. This study followed Consolidated Standards of Reporting Trials (CONSORT) reporting guidelines. The trial protocol is available in Supplement 1 and is described elsewhere.^9^

### Participants

Enrollment occurred from January 7, 2015, to November 9, 2017. Inclusion criteria were unilateral primary TKR, age 60 years or older, TKR 2 to 4 months before screening, moderate functional limitations defined by a Western Ontario and McMaster Universities Osteoarthritis Index–Physical Function (WOMAC-PF) of 9 or higher, ability to read and write English, willingness to be randomized, and medical clearance to exercise. Exclusion criteria were contraindications to exercise,^10^ neuromuscular disorders of the lower extremities, inability to independently walk 50 m, regular participation in supervised exercise, terminal illness, intent to undergo another TKR, or unavailability during the study period. Arthroplasty design, material and instrumentation, and fixation method were not considered for inclusion because they have been shown not to affect TKR outcomes.^8^

### Randomization and Masking

Adaptive randomization was used with the minimal sufficient balance algorithm^11,12^ using factors related to functional recovery, including age, sex, body mass index, surgical knee flexion, and WOMAC-PF.^8,13,14^ The system analyst created the algorithm for the randomization sequence in the electronic data capture system, and the allocation was concealed until retrieval by the research coordinator (M.B.C.) immediately after the baseline assessment. The allocation ratio was 2:2:1 to clinic-based individual outpatient rehabilitation exercise, community-based group exercise, or usual medical care. This unbalanced design was used because larger functional recovery was expected in both exercise arms compared with the usual care arm.

The assessors were masked to arm allocation. While participants could not be masked to interventions, they were asked not to discuss any aspects of the treatment with the assessors. Interventionists were masked to the participants’ outcome measurements.

### Study Arms

The interventions in this study were pragmatic based on stakeholders’ preferences and typical options currently offered to patients after TKR.^9^ Clinic-based individualized physical therapy consisted of 12 sessions (approximately 60 minutes) of exercise supervised by physical therapists (physical therapy arm) over 12 weeks. Sessions were 2 times per week in weeks 1 through 3, a single time per week in weeks 4 through 7, and then bimonthly. Each session included warm-up; moderate-intensity to vigorous-intensity (rating of somewhat hard to hard on a perceived exertion scale) resistance training of the major lower extremity muscle groups; moderate-intensity (rating of moderate to somewhat hard on the perceived exertion scale) aerobic training on a treadmill or bicycle; and functional activities, such as walking fast and in narrow paths, squatting, and rhythmic stepping. Exercises were tailored to individuals’ impairments and progressed in intensity and complexity provided they did not increase pain or effusion. Participants were taught a home exercise program and were asked to exercise at least 2 times per week (either in the clinic or at home) during the intervention phase, for a total of 24 sessions.

Community-based group exercise involved participation in supervised classes for older adults at senior community centers (community arm). Participants were asked to attend at least 2 exercise classes per week for 3 months, for a total of 24 classes (approximately 60 minutes) taught by certified senior fitness instructors. Participants were instructed to partake in evidence-based exercise classes for older adults that have shown to be challenging for active older adults and safe for more frail individuals (eg, EnhanceFitness [Sound Generations] and SilverSneakers Circuit [Tivity Health, Inc]). The program focused on dynamic cardiovascular exercise, strength training, balance, and flexibility. Attendance at each class was documented by the community centers and then sent to the research coordinator.

In the usual medical care arm, no attempt was made to interfere with the care received by participants. This arm served as a waiting list (control arm). After completing the 6-month waiting period (data collection phase), these participants were offered participation in the exercise interventions. This was done to enhance adherence and mitigate ethical concerns of not offering exercises.

### Outcomes

The primary outcome was arm differences in physical function at 3 months assessed by a patient-reported outcome measure, the WOMAC-PF.^15^ The secondary outcome of physical function was a battery of 6 performance-based tests germane to TKR endorsed by the Osteoarthritis Research Society International.^16^ These performance-based measures were used to complement the patient-reported outcome measures because they are known to capture different elements of the broad construct of physical function.^17,18,19^ Tests included the 6-minute walk, 40-m gait speed, stair ascend/descend test, single-leg stance balance, chair rise, and floor sitting and rising.^20,21,22,23,24^ These 6 test results were combined into a composite score based on the unit-weighted *z* scores of constituent tests to provide a more representative and stable measure of the participants’ underlying functional performance.^25^ This composite score was also used because not all patients shared the same functional limitations, nor do they all respond to the exercise interventions by improving in all tests. The unit weights refer to averaging standardized scores (eg, the scores for each performance-based test are converted to *z* scores before applying equal weights). Higher *z* scores represent better functional performance. The *z* scores for each participant can be interpreted as deviations from the baseline mean of the whole group.

Complementary patient-reported outcomes of physical function were recommended by the advisory panel during study setup due to concerns that the WOMAC-PF might underrepresent the high level of physical function expected at a late stage after TKR.^17,18^ These included satisfaction and performance in activities assessed by the Canadian Occupational Performance Measure (COPM),^26^ the Patient-Reported Outcomes Measurement Information System–Physical Function (PROMIS-PF)^27^ computer adaptive test, and physical health assessed by the RAND 36-Item Health Survey (RAND-36).^28^ Physical activity was assessed during 7 days using real-time accelerometry (SenseWear; BodyMedia Inc)^29^ and the Community Healthy Activities Model Program for Seniors (CHAMPS) questionnaire.^30^ Psychosocial factors were measured to explore heterogeneity of treatment response and included self-reported measures of depression,^31^ anxiety,^32^ fear of movement,^33^ and self-efficacy.^34^ Additional outcomes were adverse events, attrition, adherence, and cointerventions. Outcomes were assessed at baseline and follow-ups at 3 months and 6 months. Participants were phoned at 1.5 and 4.5 months to promote retention and assess adverse events.

### Sample Size

Eighty-six participants in the exercise arms and 43 participants in the control arm would provide 80% power to detect a mean (SD) difference of 3.3 (7.7) points between the exercise arms in the WOMAC-PF^35^ and more than 80% power to detect a 5.2-point difference between the control arm and any exercise arm (2-tailed α = .05). Assuming 10% attrition at 3 months, we proposed to recruit 240 participants (96 in the exercise arms and 48 in usual care).

### Statistical Analysis

Analysis used an intent-to-treat approach. We used linear mixed models with time and time by arm interactions using all available data for each participant (baseline, 3 months, and 6 months in the outcome vector) accounting for repeated measures per participant with unstructured covariance.^36,37,38^ The 3 arms were compared at each time point using *F* tests from the linear mixed model. We were specifically interested in contrasts at 3 months as the primary time point. The contrasts at 6 months were secondary. For the WOMAC-PF and the performance-based *z* scores, the mean differences were calculated along with 98.3% CIs due to adjustments for multiple comparisons (α = .02). No adjustments were made for the secondary outcomes, and the mean differences were calculated with 95% CIs. All models were controlled for baseline randomization covariates. We used a statistical software program (SAS, version 9.4; SAS Institute Inc).

Clinical relevance was assessed by responder analysis using several definitions of response as recommended by the Pharmaceutical Research and Manufacturers of America^39^ and comprised multiple outcome dimensions. The unidimensional definition of responder was a rating of at least moderately better in patient global rating of change in health status.^40^ The bidimensional definition was a change of at least 20% in both the WOMAC-PF and in 3 of 6 performance-based tests, consistent with international recommendations for clinical trials in arthritis.^41^ The tridimensional definition was a change of at least 50% in the WOMAC-PF, at least 20% in 2 of 6 performance-based tests, and at least somewhat better in patient global rating of change, aligned with recommendations from the Outcome Measures in Rheumatology Committee.^42^ χ^2^ Test was used for responder analysis.

Safety and adherence were compared using *t* test or χ^2^ test. Heterogeneity of treatment effects was explored by moderator analysis using the WOMAC-PF as the outcome measure and the randomization prognostic factors and psychosocial variables as potential moderators. We tested for arm by treatment effect interactions at each time point.^43^ Two-sided α was set at .05.

## Results

**Figure 1.**
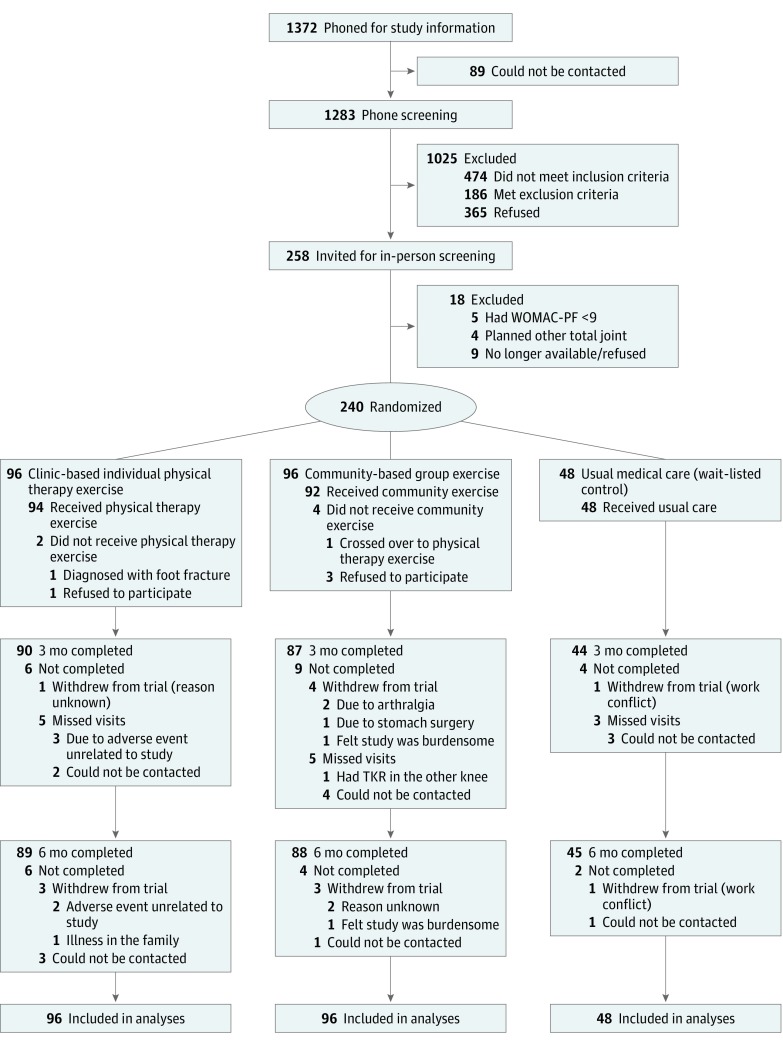
Consolidated Standards of Reporting Trials (CONSORT) Diagram of Participant Flow During the Study TKR indicates total knee replacement; WOMAC-PF, Western Ontario and McMaster Universities Osteoarthritis Index–Physical Function.

We screened 1283 people over the phone; 660 were not eligible, and 365 refused participation. Of the 258 individuals invited for study screening, 18 refused to attend or failed screening, resulting in 240 randomized (mean [SD] age, 70 [7] years; 61.7% female) (Figure 1). Attrition did not differ across arms at 3 months or 6 months: values for physical therapy were 1.0% (1 of 96) and cumulative 7.3% (7 of 96), for community were 4.2% (4 of 96) and cumulative 8.3% (8 of 96), and for control were 2.1% (2 of 48) and cumulative 6.3% (3 of 48). Baseline characteristics were not different between arms (Table 1).^44^

**Table 1.** Participant Characteristics at Baseline

Variable	Physical Therapy (n = 96)	Community (n = 96)	Control (n = 48)
Age, mean (SD), y	69 (6)	70 (7)	70 (7)
Female, No. (%)	59 (61.5)	58 (60.4)	31 (64.6)
Race, No. (%)^a^			
White	86 (89.6)	77 (80.2)	37 (77.1)
African American	10 (10.4)	18 (18.8)	11 (22.9)
American Indian/Alaskan Native	0	1 (1.0)	0
Hispanic or Latino, No. (%)	0	1 (1.0)	1 (2.1)
Education, No. (%)			
Less than college degree	31 (32.3)	33 (34.4)	12 (25.0)
Completed college or technical training	59 (61.5)	57 (59.4)	31 (64.6)
Other/missing	6 (6.3)	6 (6.3)	5 (10.4)
Married, No. (%)	64 (66.7)	62 (64.6)	31 (64.6)
BMI, mean (SD)	30.8 (5.3)	31.1 (6.3)	31.5 (5.1)
No. of comorbidities, mean (SD)^b^	4.3 (2.0)	4.4 (1.7)	4.6 (1.9)
Type of comorbidity, No. (%)			
Musculoskeletal	96 (100)	96 (100)	48 (100)
Hypertension	55 (57.3)	67 (69.8)	33 (68.8)
Eyes, ear, nose, throat, larynx	39 (40.6)	43 (44.8)	25 (52.1)
Cardiac	26 (27.1)	33 (34.4)	18 (37.5)
Upper gastrointestinal	29 (30.2)	28 (29.2)	19 (39.6)
Other genitourinary	27 (28.1)	25 (26.0)	13 (27.1)
Respiratory	28 (29.2)	25 (26.0)	12 (25.0)
Psychiatric/behavioral	30 (31.3)	16 (16.7)	12 (25.0)
Endocrine/metabolic	18 (18.8)	24 (25.0)	14 (29.2)
Vascular	19 (19.8)	24 (25.0)	11 (22.9)
Reason for TKR, No. (%)			
Osteoarthritis	92 (95.8)	92 (95.8)	48 (100)
Inflammatory arthritis	4 (4.2)	4 (4.2)	0
Time since surgery, mean (SD), d	124 (26)	124 (26)	127 (25)
Surgical knee flexion, mean (SD), degrees	124 (11)	123 (11)	124 (10)
Pain in surgical knee, mean (SD)^c^	2.7 (2.1)	2.3 (1.7)	2.2 (1.8)
Pain in nonsurgical knee, mean (SD)^c^	1.7 (2.0)	1.4 (1.7)	1.4 (2.0)

Abbreviations: BMI, body mass index (calculated as weight in kilograms divided by height in meters squared); TKR, total knee replacement.

^a^ Race and ethnicity were self-reported by participants.

^b^ Measured by the Cumulative Illness Rating Scale.^44^ Conditions with at least 20% prevalence are described.

^c^ Measured by an 11-point numeric pain scale ranging from 0 (no pain) to 10 (extremely intense pain).

Although the 3 arms demonstrated substantial improvements in all outcomes of physical function during the study, the results for the primary outcome of the WOMAC-PF do not support the benefit of the 2 exercise programs (Table 2). At the primary time point of 3 months, although the arm means for the WOMAC-PF suggested most improvement in the physical therapy arm, the adjusted analysis indicated no between-arm differences. The adjusted between-arm contrasts for the WOMAC-PF demonstrated no differences between the physical therapy and community arms (−2.2; 98.3% CI, −4.5 to 0.1), the physical therapy and control arms (−2.1; 98.3% CI, −4.9 to 0.7), and the community and control arms (0.1; 98.3% CI, −2.7 to 2.9). The results were similar and not significant at 6 months.

**Table 2.** Physical Function and Physical Activity Outcomes Over Time in Study Arms^a^

Outcome	No.	Physical Therapy, Mean (SD)	No.	Community, Mean (SD)	No.	Control, Mean (SD)	Arm *P* Value	Mean Difference (CI)		
								Physical Therapy vs Control	Community vs Control	Physical Therapy vs Community
**Physical Function**										
Patient reported using the WOMAC-PF (primary)^b^										
Baseline	96	20.9 (7.9)	96	20.4 (7.4)	48	20.2 (7.9)	NA	NA	NA	NA
3 mo	90	10.1 (6.6)	87	12.2 (7.9)	44	11.9 (7.6)	.04	−2.1 (−4.9 to 0.7)	0.1 (−2.7 to 2.9)	−2.2 (−4.5 to 0.1)
6 mo	89	9.8 (7.2)	88	10.8 (7.9)	45	11.8 (7.5)	.16	−2.1 (−5.0 to 0.7)	−0.8 (−3.7 to 2.0)	−1.3 (−3.6 to 1.0)
Performance-based test scores combined by a composite score, *z* score^c^										
Baseline	96	0.0 (0.8)	96	−0.1 (0.8)	48	−0.1 (0.7)	NA	NA	NA	NA
3 mo	89	0.5 (0.7)	87	0.3 (0.8)	44	0.1 (0.7)	<.001	0.3 (0.1 to 0.4)	0.2 (0.0 to 0.3)	0.1 (0.0 to 0.2)
6 mo	83	0.5 (0.8)	76	0.4 (0.9)	40	0.3 (0.7)	.04	0.2 (0.0 to 0.4)	0.2 (−0.0 to 0.3)	0.0 (−0.1 to 0.2)
Patient reported using the COPM-Performance^d^										
Baseline	96	3.6 (1.3)	96	3.8 (1.4)	48	4.1 (1.3)	NA	NA	NA	NA
3 mo	90	6.5 (1.7)	87	6.0 (1.8)	44	5.4 (1.7)	<.001	1.3 (0.8 to 1.9)	0.7 (0.2 to 1.3)	0.6 (0.1 to 1.0)
6 mo	89	6.8 (1.9)	88	6.6 (1.9)	45	6.0 (1.6)	.04	1.0 (0.4 to 1.6)	0.7 (0.1 to 1.2)	0.3 (−0.1 to 0.8)
Patient reported using the COPM-Satisfaction										
Baseline	96	3.0 (1.5)	96	3.3 (1.6)	48	3.2 (1.7)	NA	NA	NA	NA
3 mo	90	6.6 (1.8)	87	5.7 (2.1)	44	5.0 (2.0)	<.001	1.7 (1.1 to 2.4)	0.7 (0.1 to 1.4)	1.0 (0.5 to 1.5)
6 mo	89	6.8 (2.1)	88	6.5 (2.1)	45	5.7 (1.9)	<.01	1.3 (0.6 to 2.0)	0.8 (0.1 to 1.5)	0.4 (−0.1 to 1.0)
Patient reported using the PROMIS-PF^e^										
Baseline	96	42 (5)	96	42 (5)	48	43 (5)	NA	NA	NA	NA
3 mo	89	45 (5)	86	45 (5)	43	45 (5)	.34	1.0 (−0.4 to 2.5)	0.5 (−1.0 to 1.9)	0.6 (−0.6 to 1.7)
6 mo	89	45 (5)	84	45 (5)	45	44 (5)	.02	2.1 (0.7 to 3.6)	1.4 (−0.1 to 2.9)	0.8 (−0.4 to 2.0)
Patient reported using the RAND 36-PCS^f^										
Baseline	96	38 (7)	96	41 (7)	48	40 (8)	NA	NA	NA	NA
3 mo	90	45 (9)	87	45 (8)	44	44 (8)	.13	2.3 (−0.2 to 4.7)	0.7 (−1.8 to 3.2)	1.6 (−0.4 to 3.6)
6 mo	89	46 (9)	88	45 (9)	45	44 (10)	.03	3.4 (0.5 to 6.2)	0.9 (−2.0 to 3.7)	2.5 (0.2 to 4.8)
**Physical Activity**^g^										
Measured by a portable accelerometer, kcal/d										
Baseline	96	511 (411)	95	541 (419)	47	459 (352)	NA	NA	NA	NA
3 mo	85	566 (451)	86	547 (407)	43	525 (411)	.91	4 (−106 to 114)	−15 (−125 to 95)	19 (−71 to 109)
6 mo	85	538 (403)	80	480 (420)	40	509 (518)	.61	15 (−112 to 142)	−36 (−164 to 92)	51 (−52 to 154)
Patient reported using the CHAMPS, kcal/wk										
Baseline	96	4125 (2954)	96	3840 (3124)	48	3448 (2793)	NA	NA	NA	NA
3 mo	90	4489 (2907)	87	4791 (3539)	44	3765 (2896)	.35	305 (−615 to 1224)	659 (−265 to 1583)	−355 (−1105 to 396)
6 mo	89	3956 (2695)	88	4781 (3739)	45	3540 (2724)	.03	−33 (−939 to 872)	896 (−11 to 1803)	−930 (−1672 to 188)

Abbreviations: CHAMPS, Community Healthy Activities Model Program for Seniors questionnaire; COPM, Canadian Occupational Performance Measure; NA, not applicable; PROMIS-PF, Patient-Reported Outcomes Measurement Information System–Physical Function; RAND 36-PCS, RAND 36-Item Health Survey Physical Component Scores; WOMAC-PF, Western Ontario and McMaster Universities Osteoarthritis Index–Physical Function.

^a^ The means (SDs) of outcomes in study arms at each time point are based on complete cases. The results from the statistical tests and mean differences (CIs) for contrasts between arms are based on linear mixed models and include all participants originally randomized to each arm. The models are adjusted by age, sex, body mass index, surgical knee flexion, and baseline outcome. The CIs for the WOMAC-PF and *z* scores of functional performance represent 98.3% CIs. For the other outcome measures, the CIs represent 95% CIs.

^b^ The WOMAC-PF consists of 17 items scored on a 5-point Likert-type scale (version LK3.1). Scores of each item are summed, for a maximum total score of 68. Higher scores indicate worse functional limitations.^15^

^c^ Composite *z* score formed with unit-weighted *z* scores of the following 6 performance-based tests: 6-min walk, 40-m gait speed, stair ascend/descend test, single-leg stance balance, chair rise, and floor sitting and rising.^20,21,22,23,24^ Higher *z* scores represent better functional performance. The *z* scores for each participant can be interpreted as deviations from the baseline mean of the whole group (eg, a change in *z* score of 0.1 represents approximately 10% of an SD relative to the baseline mean of the whole group).

^d^ The *t* statistics range from 30 to 70. Higher scores represent better physical function.^27^

^e^ Scores range from 0 to 100, with higher scores representing better physical health.^28^

^f^ In the COPM, the patients identify up to 5 activities that are limited, and performance and satisfaction are quantified (0-10 scale).^26^ For COPM-Performance and COPM-Satisfaction, scores are the mean across activities and range from 0 to 10 (higher scores represent better performance and satisfaction). The same activities identified during baseline were used for follow-ups.

^g^ Estimated as energy expenditure during light-intensity activities or above (≥1.5 metabolic equivalent tasks) to account for intensity and time of activities.

For the secondary outcome of performance-based function, the adjusted between-arm contrasts at 3 months showed better functional performance in the physical therapy arm compared with both the community (0.1 *z* score units; 98.3% CI, 0.0-0.2) and control (0.3 *z* score units; 98.3% CI, 0.1-0.4) arms and the community arm compared with the control arm (0.2 *z* score units; 98.3% CI, 0.0-0.3) (Table 2). At 6 months, although the physical therapy arm continued to have better functional performance compared with the control arm (0.2; 98.3% CI, 0.0-0.4), there were no differences between the physical therapy and community arms (0.2 *z* score units; 98.3% CI, −0.0 to 0.3) or between the community arm compared with the control arm (0.0 *z* score units; 98.3% CI, −0.1, to 0.2). Descriptive characteristics of the individual performance-based tests that form the *z* score are available in the eFigure and eTable 1 in Supplement 2.

Complementary patient-reported outcomes at 3 months demonstrated better COPM-Performance and COPM-Satisfaction in the physical therapy arm compared with both the community and control arms and the community arm compared with the control arm (Table 2). There were no between-arm differences for PROMIS-PF and RAND-36 physical component score. At 6 months, the physical therapy arm had better COPM-Performance and COPM-Satisfaction compared with the community and control arms and the community arm better than the control arm. The PROMIS-PF was better in the physical therapy arm compared with the control arm, and RAND-36 physical component score was better in the physical therapy arm compared with the community and control arms. The findings for physical activity were not significant.

**Figure 2.**
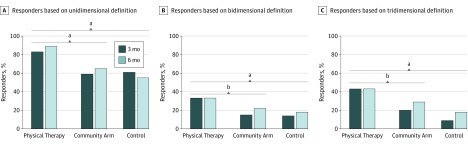
Responder Analysis With Percentage of Responders by Arm for Each Definition of Treatment Response A, Response defined as a rate of at least moderately better in patient global assessment of change in health status. B, Response defined as more than 20% improvement in both the Western Ontario and McMaster Universities Osteoarthritis Index–Physical Function (WOMAC-PF) and at least 3 of 6 tests of performance. C, Response defined as more than 50% improvement in the WOMAC-PF, more than 20% improvement in at least 2 of 6 tests of performance, and a rate of at least somewhat better in patient global assessment of change in health status. ^a^*P* < .05 for between-arm contrast at 3 and 6 months. ^b^*P* < .05 for between-arm contrast at 3 months.

Responder analysis showed a greater proportion of responders in the physical therapy arm compared with the community or control arms, which was consistent across all definitions of response at 3 months. The absolute differences between the physical therapy and control arms were 21.9% (83.3% vs 61.4%, *P* = .005) for unidimensional, 19.0% (32.6% vs 13.6%, *P* = .02) for bidimensional, and 33.6% (42.7% vs 9.1%, *P* < .001) for tridimensional definitions of response. The absolute differences between the physical therapy and community arms were 24.7% (83.3% vs 58.6%) for unidimensional, 17.7% (32.6% vs 14.9%) for bidimensional, and 23.2% (42.7% vs 19.5% for tridimensional definitions of response (*P* < .01 for all) (Figure 2). At 6 months, the absolute differences between the physical therapy arm and the other arms were attenuated and ranged from 10.2% to 35.3%. No differences were found between the community and control arms at any time point.

There were no serious adverse events related to study participation (Table 3). As anticipated, more reports of joint pain and soft-tissue soreness occurred in the exercise arms, which typically resolved within days or weeks. Subsequent joint replacement and intent to seek additional treatments were not different across arms. A higher percentage of participants in the control arm (43.8% [21 of 48]) engaged in substantial exercise outside the study compared with the exercise arms (21.9% [21 of 96] and 18.8% [18 of 96]). The physical therapy arm participated in more study-prescribed exercise (mean [SD], 11 [1] supervised and 21 [7] home sessions, for a total of 32 [7] sessions) than the community arm (mean [SD], 18 [10] exercise classes) (*P* < .001).

**Table 3.** Adverse Events, Cointerventions, and Adherence

Variable	No. (%)			*P* Value
	Physical Therapy (n = 96)	Community (n = 96)	Control (n = 48)	
**Adverse Events**				
Related to study participation				
Arthralgia	12 (12.5)	7 (7.3)	1 (2.1)	.10
Back pain	1 (1.0)	2 (2.1)	0	.81
Fall	0	1 (1.0)	1 (2.1)	.68
Myalgia	0	1 (1.0)	0	>.99
Other musculoskeletal and connective tissue	5 (5.2)	0	0	.03
Skin and subcutaneous tissue	1 (1.0)	0	0	>.99
Related to study participation, severity greater than mild				
Arthralgia	10 (10.4)	6 (6.3)	1 (2.1)	.01
Back pain	1 (1.0)	2 (2.1)	0	.81
Other musculoskeletal and connective tissue	2 (2.1)	0	0	.36
Related or not to study participation with >5% in a single arm				
Arthralgia	18 (18.8)	17 (17.7)	8 (16.7)	.95
Back pain	7 (7.3)	9 (9.4)	5 (10.4)	.84
Other musculoskeletal and connective tissue	13 (13.5)	13 (13.5)	6 (12.5)	>.99
Cataract	1 (1.0)	3 (3.1)	3 (6.3)	.21
Fall	4 (4.2)	4 (4.2)	2 (4.2)	>.99
Related or not to study participation with >5% in a single arm, severity greater than mild				
Arthralgia	15 (15.6)	15 (15.6)	8 (16.7)	.98
Back pain	5 (5.2)	8 (8.3)	2 (4.2)	.65
Other musculoskeletal and connective tissue	8 (8.3)	7 (7.3)	6 (12.5)	.63
Cataract	1 (1.0)	3 (3.1)	3 (6.3)	.21
Fall^a^	3 (3.1)	2 (2.1)	1 (2.1)	>.99
**Cointerventions**				
TKR in the other knee	2 (2.1)	2 (2.1)	0	.69
TKR revision	0	0	0	NA
Total hip replacement	0	0	1 (2.1)	.20
Sought health professional for knee pain	16 (16.7)	24 (25.0)	8 (16.7)	.29
Sought health professional for pain elsewhere	31 (32.3)	27 (28.1)	14 (29.2)	.81
Engaged in substantial exercise outside the study^b^	21 (21.9)	18 (18.8)	21 (43.8)	.005

Abbreviations: NA, not applicable; TKR, total knee replacement.

^a^ Falls are reported regardless of less than 5% occurrence because it was a prespecified adverse event.

^b^ Defined as participation in exercise of moderate intensity for 30 minutes at least 2 times per week.

Moderator analysis (eTables 2, 3, and 4 in Supplement 2) at 3 months demonstrated a larger treatment effect of the physical therapy arm compared with the control arm among participants with low self-efficacy function and a larger treatment effect of the community arm compared with the control arm among the subgroup with high self-efficacy symptoms. At 3 months and 6 months, those with higher levels of anxiety/depression appeared to experience less improvement for the community arm compared with both the control arm and the physical therapy arm. At 6 months, there were larger treatment effects for the physical therapy and community arms (compared with the control arm) among the subgroups of nonobese participants and those with more surgical knee flexion.

## Discussion

This study provides new evidence about the safety and effectiveness of exercise programs at a late stage after TKR. The results based on the primary outcome of the study do not support the benefit of either of 2 exercise programs over usual care. The differences in the WOMAC-PF across the arms were small and not clinically important, demonstrating that there were no benefits of clinic-based physical therapy exercises or community-based group exercises at a later stage after TKR. In addition, both exercise programs were demonstrated to be safe.

The results based on the secondary outcomes suggest that participation in physical therapy seems to provide greater improvements than community-based group exercise or usual care. These findings were demonstrated for the between-arm tests of functional performance and some of the complementary patient-reported outcomes. Responder analysis also showed a greater proportion of responders in the physical therapy arm (≥17.7% more responders at 3 months and ≥10.2% more responders at 6 months) compared with the community or control arms. The secondary outcomes also suggested a small benefit of community-based exercise vs usual care for some measures, but these findings were not robust or clinically important.

The paradox of negative results based on the primary outcome yet positive results based on the secondary outcomes is intriguing. While patient-reported outcomes like the WOMAC-PF evaluate what individuals perceive they can do, performance-based tests evaluate what individuals can actually do. The latter were selected to complement the assessment of the broad construct of physical function based on recommendations from international organizations in osteoarthritis.^16^ Studies^17,45^ indicated that while patients tend to self-report improvement in their ability to complete functional tasks (eg, climbing stairs or walking) in the WOMAC-PF after TKR, their objectively measured performance during these tasks actually worsens. These discrepancies are partially explained by the association between reduced knee pain and an inflated self-reported perception of improved mobility in the WOMAC-PF.^17,18,19^ Another plausible explanation for these conflicting results is the potential for the WOMAC-PF to underrepresent high levels of functional performance expected by patients who are at a later stage of recovery after TKR,^17,18^ a concern that was raised by the advisory panel of this study. Emerging evidence suggests that the items in the WOMAC-PF underrepresent the functional activities identified as important by patients at a later stage after TKR, particularly the more physically demanding activities, such as kneeling, squatting, carrying objects, transfers to and from the floor, yard work, and walking up and down hills and curbs.^46^ Moreover, although the mean baseline values in the WOMAC-PF (approximately 20 points; range, 0-68 points) represent moderate functional limitations and would allow sufficient room for improvement, it is possible that several participants enrolled in the study had low levels of patient-reported functional limitation and consequentially had limited room for improvement.

The improvements over time in all arms were above the published thresholds for clinically important improvement for each measurement and tended to be largest in the physical therapy arm. For example, the cut point for the WOMAC-PF is 20% improvement relative to baseline,^41^ and all arms herein improved at least twice that amount (53% for physical therapy, 47% for community, and 42% for control). For the 6 performance-based tests that form the *z* score, all arms surpassed the cut points of 2.7 seconds for the stair ascend/descend test,^20^ 0.05 m/s for gait speed,^47^ and 20 m in the 6-minute walk test.^48^ For the latter, the improvement in the physical therapy arm at 3 months was double (50 m) that of the control arm (25 m). For the 5 times sit to stand test, both exercise arms surpassed the cut point of 2.3 seconds,^49^ whereas the control arm did not. While cut points are not available for single-leg stance balance or floor sitting and rising, improvements in the physical therapy arm at 3 months were visibly larger. For the complementary patient-reported outcomes, only the physical therapy arm surpassed cut points for COPM-Performance and COPM-Satisfaction (3.0 and 3.2, respectively).^50^ For the RAND-36 physical health,^51,52^ the cut points range from 5 to 7 points, which was only achieved in the physical therapy arm. We are not aware of published cut points for PROMIS-PF and physical activity.

The differences between the arms based on the secondary outcomes, although favoring physical therapy exercise, were modest and require confirmation. It is possible that later-stage exercise may be too late after surgery to promote relevant benefits. Perhaps a better model of later-stage exercise delivery (not tested in this study) would be a 2-stage approach. Individualized physical therapy could be delivered first to address the persistent functional limitations of selected patients, followed by long-term group exercise delivered in a community setting to promote sustained benefit. However, this model needs exploration in future trials with longer-term follow-ups.

Moderator analysis identified that not all patients benefit from interventions in the same way. Community-based exercise does not seem like a good alternative for patients with high anxiety/depression and low self-efficacy, who show only small improvements. For these patients, physical therapy may be particularly beneficial because individualized therapy generally uses psychological strategies to overcome anxiety and promote self-efficacy, and the exercises can be tailored during therapy to the limitations of each patient. The results also suggest that nonobese patients and those with more surgical knee flexion might experience added benefit from either exercise intervention compared with obese patients and those with limited surgical knee flexion.

The clinically important improvements in physical function in the control arm were not anticipated because participants were on average 4 months after TKR, a time frame when the literature has shown a plateau in functional recovery.^53,54^ However, the results from this study suggest that the outcomes of TKR continue improving longer than suggested in the literature. It is also possible that the improvements in the control arm observed during the study could represent a Hawthorne effect.^55^ Agreeing to be part of an exercise study may have influenced the participants’ behavior and increased engagement in substantial exercise outside the study, which was found to be 2 times higher in the control arm than in the other study arms.

### Limitations

Our study has some limitations. The study had a short follow-up of 6 months, which was constrained by a 3-year funding period. Based on other exercise studies,^56,57,58^ we anticipate that the modest benefits of exercise would be even less at longer follow-ups unless the individuals continue to exercise. Another limitation was not including a cost-effectiveness analysis,^59^ which was due to funding agency restrictions. It is expected that physical therapy would be more expensive, and the additional cost may not be meaningful to this population. There are also inherent differences in the amount of personalized attention, intensity, and individualization of the exercises between individualized physical therapy and community group exercises, which may have introduced bias.

## Conclusions

This study provides new evidence about the safety and effectiveness of exercise programs at a late stage after TKR. The results based on the WOMAC-PF (primary outcome) demonstrated no benefit of later-stage exercise after TKR. However, the findings based on the secondary outcomes suggested greater improvement in the physical therapy arm, but these require confirmation. Both exercise programs were safe at a later stage after TKR. The results from moderator analysis suggest that individuals with higher anxiety/depression symptoms seemed to benefit more from the physical therapy arm and less from the community arm.

## Notes

Supplement 1. **Trial Protocol**

Supplement 2. **eFigure.** Graphs of Individual Performance-Based Tests Over Time **eTable 1.** Performance-Based Tests Over Time in Study Arms **eTable 2.** Baseline Values of Variables Tested as Potential Moderators **eTable 3.** Mean Values of Physical Function Measured by WOMAC-PF and Number of Participants at Each Time Point for the Levels of Potential Moderators **eTable 4.** Test of Heterogeneity of Treatment Effects at Each Time Point

Supplement 3. **Data Sharing Statement**
