# Supplementary material for: Effectiveness of Later-Stage Exercise Programs vs Usual Medical Care on Physical Function and Activity After Total Knee Replacement: A Randomized Clinical Trial
Source: JAMA Netw Open. 2019 Feb 22;2(2):e190018. doi: 10.1001/jamanetworkopen.2019.0018 (PMC6484600; doi:10.1001/jamanetworkopen.2019.0018)
Supplement: Supplement 1. — Trial Protocol [file jamanetwopen-2-e190018-s001.pdf]

Supplement 1

This supplement contains the following items:

1. Original protocol, and final protocol with amended changes.

2. The statistical analysis plan (including initial and final analysis plan).

Manual of Operational Procedures (MOP)

for the research study:

“A comparison of treatment methods for patients following total knee replacement”

Principal Investigator:

Sara R. Piva, PhD, PT

Supported by the Patient Centered Outcomes Research Institute (PCORI)

CER-1310-06994

Version 1.0

2015

Table of Contents

1. STUDY OBJECTIVES 5

1.1. Primary Aim 5

1.2. Secondary Aim 5

1.3. Exploratory Aim 5

2. BACKGROUND 5

3. STUDY DESIGN 7

3.1. Overview of Study Design 7

3.2. Summary of Study Procedures 10

3.3. Summary of Study Recruitment and Screening Procedures 11

4. SELECTION AND ENROLLMENT OF SUBJECTS 12

4.1. Inclusion/Exclusion Criteria 12

4.2. Telephone Screening Process and In-person Eligibility Screening Visit Scheduling 13

4.3. In-person Eligibility Screening 14

5. BASELINE TESTING 15

5.1. Self-Reported Measures: 15

5.2. Clinical Examination 15

6. RANDOMIZATION 18

7. MASKING 18

8. STUDY INTERVENTIONS 19

8.1. Clinic-based Individual Outpatient Rehabilitative Exercises 19

8.2. Community-based Group Exercise 19

8.3. Usual Medical Care (waited-list control group) 20

9. EXERCISE COMPLIANCE 20

10. FOLLOW-UP PHONE-CALLS 20

11. FOLLOW-UP IN-PERSON TESTING 21

11.1. Three and Six Months Testing Sessions (and Nine Months for the Usual Medical Care Group) 21

11.2. Wait-list Usual Care group at the 6 months visit 22

12. CLINICAL MEASURES 23

12.1. Outcome Measures 23

12.1.1. Primary Outcome Measure 23

12.1.2. Secondary Outcome Measure 23
12.1.3. Other Measures 24
13. ADVERSE EVENTS 25
13.1. Management 25
13.2. Report 25
14. STUDY COMPENSATION 25
15. DATA ANALYSIS AND SAMPLE SIZE JUSTIFICATION 26
15.1. Primary Hypothesis 26
15.2. Secondary Hypothesis 26
15.3. Exploratory Aim 27
16. DROPOUT AND MISSING DATA 27
17. RECORD KEEPING 28
18. DATA MANAGEMENT PLAN 29
19. QUALITY ASSURANCE 30
20. DATA SAFETY AND MONITORING BOARD 30
21. HUMAN SUBJECTS 32
21.1. Institutional Review Board (IRB) Review and Inform Consent 32
21.2. Risks/Benefits Assessment 32
21.3. Potential Risks 32
21.4. Risk Management and Emergency Response 32
21.5. Confidentiality 33
21.6. Potential Benefits of the Proposed Research to Human Subjects and Others 33
21.7. Importance of the Knowledge to be Gained 33
22. STUDY ORGANIZATION AND ADMINISTRATION 33
22.1. Administration/ Research Personnel 33
23. STUDY PLACES 34
23.1. Community Centers 34
23.2. PT-CTRC 35
24. RESEARCH ENGAGEMENT PLAN 35
25. STUDY OPERATIONS 36
26. PUBLICATIONS 36
27. REFERENCES 37

APPENDIX *(documents from appendix are available upon request)* 42
A. Study Forms 42
B. Study Forms Coded 42
C. Scoring 42
D. Informed Consent 42
E. Study Advertisement 42

Table of Figures

Figure 1- Study Design 8
Figure 2 - Study Flow Chart 9

Table of Tables

Table 1. Clinical measures completed at each time point. 23
Table 2. Type of data storage 28

1. STUDY OBJECTIVES

This study is a pragmatic comparative effectiveness study of exercise for patients following total knee replacement (TKR), designed as a 3-group randomized clinical trial. The main goal is to provide evidence to inform the choice of exercise programs during later stages after TKR.

1.1. Primary Aim

The primary aim of the study is to compare the outcomes of physical function and physical activity between the three groups: 1) clinic-based individual outpatient rehabilitative exercise; 2) community-based group exercise and 3) usual medical care waited-list.

The first hypothesis is that subjects in groups 1 and 2 will demonstrate greater improvement in physical function as compared to group 3. Physical function will be assessed using a self-reported questionnaire the Western Ontario and McMaster Universities Arthritis Index-WOMAC, and assessed by a battery of performance-based tests germane to patients post-TKR (walking speed, chair rise, stair climbing, single leg stance, 6-minute walk, and rising from the floor). The second hypothesis is that subjects in groups 1 and 2 will demonstrate larger increases in physical activity as compared to group 3. Physical activity will be measured in real-time using a portable monitor (SenseWear Armband) and by a questionnaire (Community Healthy Activities Model Program for Seniors-CHAMPS).

1.2. Secondary Aim

The secondary aim of this study is to identify baseline predictors of functional recovery for both exercise groups. The hypothesis with this aim is a group of baseline biomedical (age, sex, education, chronicity of disease, physical function), physical impairments (range of motion, pain, muscle strength), and psychosocial measures (fear of activity, coping, depression, self-efficacy, expectation) will be associated with treatment response. The expectation is that the predictors of treatment response to be different in the treatment groups.

1.3. Exploratory Aim

This study also has an exploratory aim to determine attrition, adherence, adverse events and co-interventions across treatment groups. The hypothesis related to this aim is adherence and co-interventions will be similar in all groups. The attrition rate and adverse events- mainly number of falls- will be lower in groups 1 and 2 compared to group 3.

This study will inform the choice of interventions for later stages after TKR and will provide evidence for the design of public health programs to extend the number of years free of disability in this population and to tailor interventions according to patient characteristics.

2. BACKGROUND

The demand for TKR is growing exponentially. Over 4 million US adults currently live with a TKR and it is projected that greater than 3 million TKRs will be performed annually in the US by 2030.[1] The lifetime risk of undergoing TKR is 8% for all persons.[2] TKR represents the highest aggregate cost among the fast increasing surgical procedures, posing a large economic burden on the US health system.[3] Patients who undergo TKR experience considerable functional limitations, muscle weakness, de-conditioning, physical activity,[4-7] and also

represent a rapidly increasing population with multiple comorbidities. Around 49% are overweight or obese, 16% have diabetes and 50% have high blood pressure.[8,9]

By most metrics, TKRs are successful surgeries, as they reduce pain and are cost-effective. [10,11] However, long-term functional and activity limitations, due to chronic joint disease prior to surgery, do not spontaneously resolve after TKR. Limitations in basic activities such as walking and managing stairs along with knee pain remain for years after TKR.[12-18] A study found that after one year, 52% of subjects who have received TKR surgeries continued to have substantial limitations during biomechanically demanding activities such as kneeling, squatting, turning and cutting, carrying loads, playing tennis, dancing, gardening, and sexual activity, in contrast to only 22% of matched controls.[19] Subjects post-TKR are also at increased risk for falls, [20] do not reach recommended levels of physical activity to prevent morbidity, [21] and gain weight in the years after surgery. [22] Patients have voiced that aside from the stress and disruption in their lives caused by the TKR, they are not getting the later benefit they expected.[23]

Persistent functional limitations combined with physical inactivity post-TKR are a major public health concern as they are precursors to disability and comorbidities. For example, decreased walking speed is a risk factor for falls, future disability, and mortality,[24-27] physical inactivity is a risk factor for hypertension, diabetes, obesity, cardiovascular disease, and cognitive decline.[28] These limitations can also affect the ability of older adults to stay in the workforce and live independently. Hence, there is an urgent need to overcome the persistent disability and physical inactivity of these patients to averting permanent disability and comorbid conditions.

Rehabilitation is a simple solution to alleviate the functional limitations, promote physical activity, and enhance TKR outcomes. While virtually all patients receive rehabilitation for about 1-2 months (early stage) after surgery,[29-31] studies demonstrated very modest benefits of rehabilitation during the first months post-TKR.[28] These modest benefits are not surprising; during the initial months post-TKR, patients are still healing from the major surgical wounds, and rehabilitative exercises are only able to focus on improving knee movement and promoting safe and independent mobility. It is not realistic to expect that early post-op rehabilitation alone can reverse years (or decades) of pre-operative muscle weakness, deconditioning and functional limitations. Participation in more extended exercise programs that intensively target the muscle weakness, deconditioning, and poor mobility is likely the only way to reverse these persistent deficits. Exercise intervention should be continued at a later stage post-TKR when patients can tolerate intense doses of exercise required to promote substantial changes.

Despite the misconception that patients reach a limit in their recovery within a few months post-TKR, emerging evidence indicates that patients who perform intense exercise at later stages post-TKR (at least 2 months post-op) achieve substantial functional recovery.[32-35] While the collective findings from these studies emphasize that patients can tolerate intense exercise programs at later stages post-TKR and that it has potential to recover function, the evidence from these studies is limited due to small samples or non-rigorous research methods. [32-35]

The effect of exercise used at later stage post-TKR has been an understudied area of research with a large gap in knowledge that affects patients, clinicians and extends to healthcare payers and policymakers. They lack the compelling evidence to alter healthcare policy decisions about the management of patients at later stage post-TKR, to enhance the outcome of this prevalent and expensive surgery. Due to currently limited evidence, only a

small fraction of patients are referred for rehabilitative exercises at later stages (2 months or more) post-TKR. This results in the majority of patients receiving insufficient care. They are prematurely discharged from rehabilitation before exercises can be intensified to enhance surgical outcome. Healthcare providers state they simply lack good evidence for such recommendations, [23] leaving patients and providers without guidance to inform decisions on prevention of morbidity and maximize the benefits of TKR.

3. STUDY DESIGN

3.1. Overview of Study Design

This comparative effectiveness study is designed to combine patient-centered research questions with rigorous research methods that minimize bias and balance internal and external validity. The study is designed as a three-group single-blind randomized clinical trial. Eligible subjects undergo baseline assessment and are randomized in a 2:2:1 allocation to one of the 3 groups: 1) clinic-based individual outpatient rehabilitative exercise; 2) community-based group exercise classes; or 3) usual medical care. The usual medical care group continues their usual care whereas the other two groups receive an exercise intervention for 12 weeks. Endpoint measures are assessed in-person at 3 and 6 months after randomization. Participants are also interviewed over the phone at 1.5 and 4.5 months after randomization to promote retention. After the 6 month follow-up, subjects in the usual medical care group are randomized to either clinic-based individual outpatient rehabilitation exercise or community-based group exercise and participate in a phone interview at 7.5 months and in-person assessment at 9 months after initial randomization. Figure 1 gives an overview of study design.

The design of this study is unbalanced with an unequal number of subjects per group, meaning that the exercise groups have twice the number of subjects as the control group. The unbalanced design was chosen because larger functional recovery is expected in both exercise groups as compared to the usual care group, thus requiring larger sample size in the two exercise arms to detect smaller differences between them as compared to larger differences expected between either of the exercise arms and the usual care arm.

Figure 1- Study Design

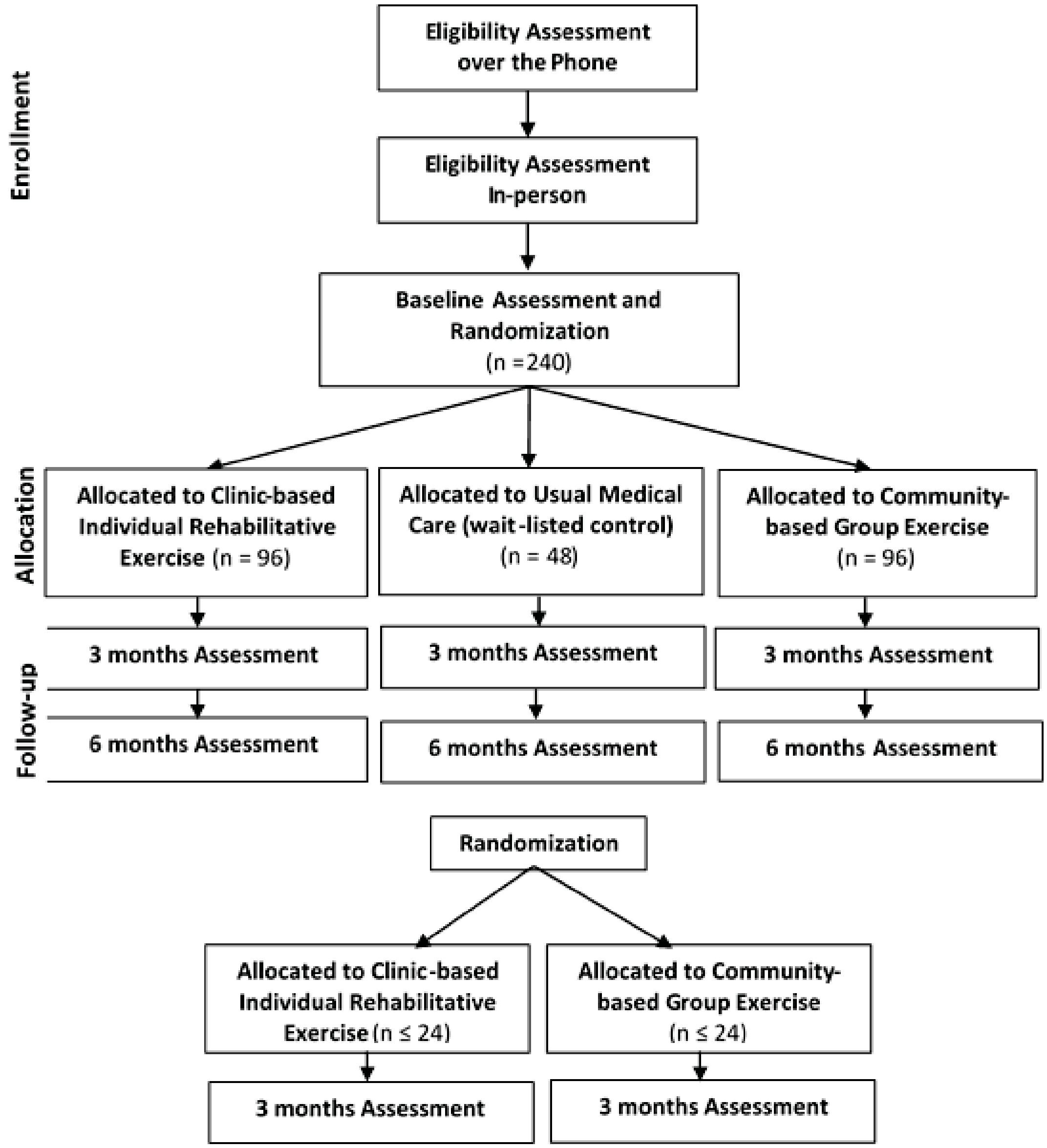

Figure 2 - Study Flow Chart

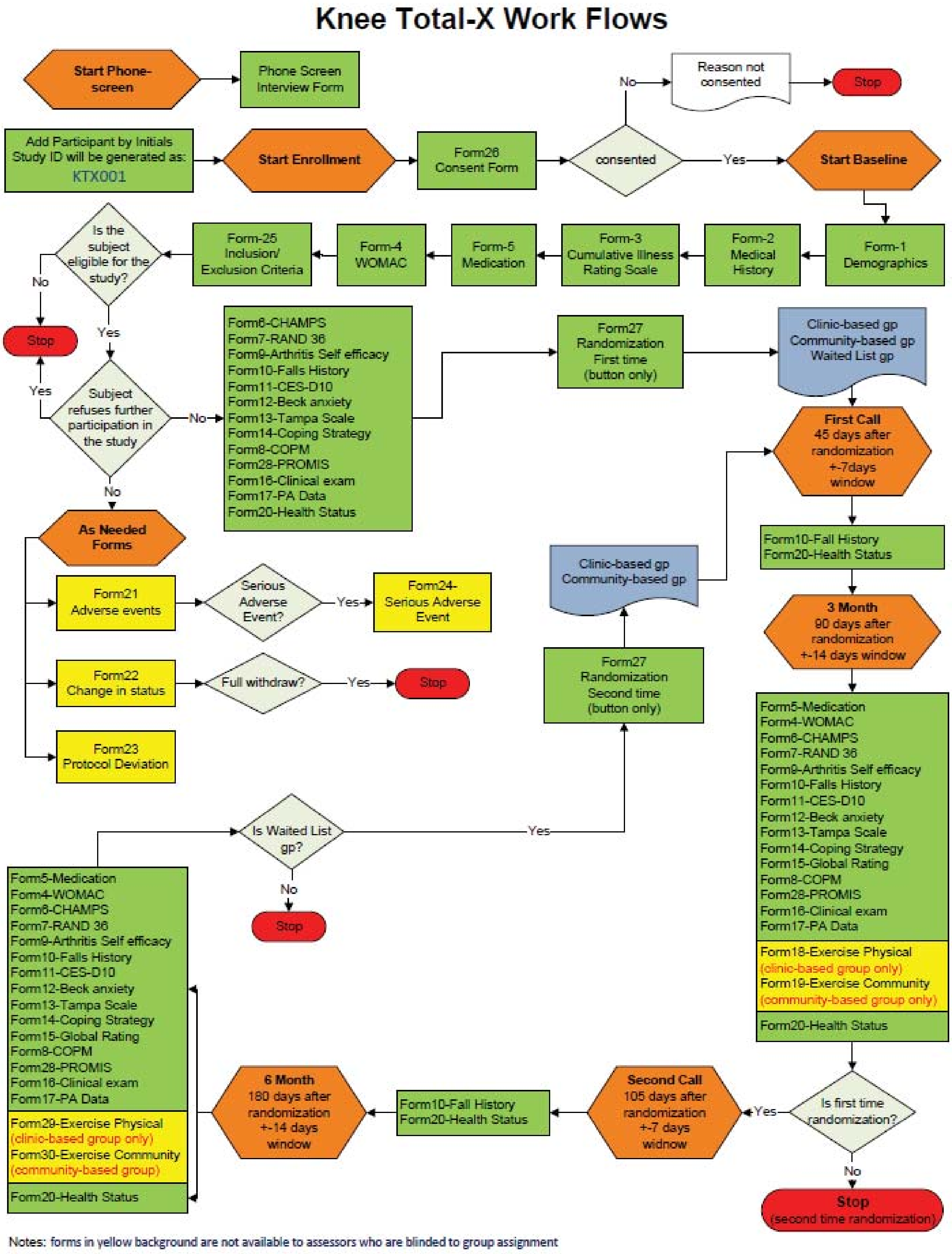

3.2. Summary of Study Procedures

For organization purposes, this study is divided in 6 main elements of study procedures. Figure 2 describes the flow chart of these procedures along with paths to continue or not study procedures, and the main elements are shown in the orange hexagons.

3.2.1. Telephone Screening: potential subjects that called the research team are contacted for telephone screening by the study research coordinator/trained research assistants. During this phone call, information about the study is given to subjects and they are screened for eligibility to participate in the study.

3.2.2. Medical Release process: subjects deemed eligible on the phone screening need clearance from the surgeon to schedule the in-person screening visit. A medical release is obtained by the research assistants and the coordinator of the study, through fax or email. Study research assistants send the Medical Referral form to the surgeons (or their assistants), and other physicians (e.g. cardiologist, primary care physician, etc) in case there is any concern with other health conditions.

3.2.3. Schedule in-person eligibility visit: after the medical release is obtained, the research coordinator can schedule in-person eligibility visit. The tentative appointment is scheduled with the subject, and it is confirmed after tester confirms availability. Once this is done, a package for in-person eligibility visit is prepared and mailed to the subject, containing: a letter with general instructions for the appointment, informed consent and driving directions to the research facility with parking instructions.

3.2.4. Call to confirm appointment: research coordinator calls subjects scheduled for the next day to confirm/reschedule the appointment. During this phone call, the coordinator must ask if the subject received, and if he/she had any questions on, the material sent in the eligibility package.

3.2.5. In-person screening visit: Trial coordinator goes over the informed consent document with the subject to clarify any questions. If the subject agrees with all study procedures, both the subject and the research coordinator sign 2 copies of the informed consent document (one copy stays with the coordinator and the other one is given to subject). The research coordinator turns study laptop on, connects the laptop to the Wi-Fi (if not done automatically) and open KTX database (http://www.crhc.pitt.edu/KneeTotalX/Default.aspx). Once in the KTX database webpage, the research coordinator logs in and add the new subject into the system (subject is given a study identification number). The subject is asked to complete questionnaires (Demographics, Medical History form and the Western Ontario and McMaster Universities Osteoarthritis Index Physical Function Subscale - WOMAC-PF) in the database, and the research coordinator checks the list of current medications (provided by the subject), and interviews the subject to collect comorbidity data using the Cumulative Illness Rating Scale. The Inclusion/Exclusion form is a smart form (using data collected in the screening visit) to deem subject's final eligibility to participate in the study.

3.2.6. Baseline visit: usually occurs on the same day immediately after the in-person eligibility screening visit. A series of self-reported questionnaires are completed by the eligible subject followed by a clinical evaluation.

3.2.7. Intervention: delivered for 3 months. Intervention sessions can be extended if there is a medical condition that requires immediate treatment and interruption of the intervention. Intervention will resume after release from the treating physician and/or subside once symptoms are resolved. Intervention will be discontinued if the subject was harmed during the sessions or deemed unsafe.

3.2.8. Phone follow-up: occurs every 1.5 months subjects are in the study. The trial coordinator calls subjects at every 1.5 months once they are in the study to track adverse events, exercise compliance and co-interventions. The window for the phone calls to be done is 1 week prior and after to phone call target date (totalizing 2 weeks).

3.2.9. In-person follow-up testing sessions: scheduled after 3 months, 6 months and 9 months from the randomization date. The window for the testing sessions to be scheduled is 1 week prior to the target date, and 3 weeks after the target date (totalizing 1 month).

3.3. Summary of Study Recruitment and Screening Procedures

The study uses several recruitment strategies with the intent of enrolling 8 to 10 subjects each month. Study recruitment started in December 2014 and it will be continued until December 2016. The primary strategy comprises of invitations sent to recent TKR patients directly from the knee surgeons who performed the procedure. Knee surgeons recruit participants either during a regular follow-up visit or send letters to their patients offering study participation. We anticipate participation in this recruitment strategy from 10 to 15 knee surgeons from several clinics, and who operate in different hospitals within Allegheny County.

Additional recruitment strategies include direct mailings of postcards to local neighborhoods, study letters sent to participants of research registries (Clinical and Translational Science Institute -CTSI - Registry and the Pittsburgh Claude D. Pepper Older Americans Independence Center Registry), advertisements on local radio stations, newspapers, and other publications. The study also recruits subjects directly from the Vintage Community Senior Center and the Squirrel Hill Jewish Community Center. Both centers are designated community senior citizen centers by the Allegheny County Area Agency on Aging. The directors of these centers have agreed to assist with the study's recruitment efforts by allowing posters and informational brochures to be placed in their facilities, by sending e-blasts to their respective members, and by posting announcements about the research study in their newsletters.

For all recruitment efforts, we use services from the University of Pittsburgh - University Marketing Communications (UMC). The UMC is a resource available to researchers at the University of Pittsburgh, which has a full-time staff that provides advertising, planning, copy-writing, design, and production services and handles reservation of newspaper space.

All recruitment materials have the research coordinator's telephone number so that subjects may contact the research team, if interested in the study. Thus, regardless of the recruitment method, the potential subjects initiate contact with study personnel. All individuals who call to inquire about the study give their verbal consent to undergo telephone screening. Those deemed potentially eligible over the phone are scheduled for an in-person assessment to sign informed consent and re-confirm eligibility. If eligibility is confirmed during the in-person assessment, the participant undergoes an in-person screening assessment (Figure 1).

4. SELECTION AND ENROLLMENT OF SUBJECTS

4.1. Inclusion/Exclusion Criteria

The study enrolls adults older than 60 years of age from Allegheny County who underwent a unilateral TKR 2 to 4 months prior to study participation. Thus, subjects have healed from the surgical insult and knee pain, effusion and motion are improved, and are no longer restricted from more intense exercises. Participants also have to experience at least moderate functional limitation in daily activities to represent those with persistent limitations after TKR (minimum score of 9 points on the Western Ontario and McMaster Universities Osteoarthritis Index Physical Function Subscale -WOMAC-PF), speak English sufficiently to understand study instructions, be willing to be randomized to one of the three treatment groups, and have a medical clearance to participate in the study. Subjects will be excluded from the study if they meet any the following criteria:

- Absolute or relative contraindication to exercise testing by the American College of Sports Medicine/American Heart Association
  - Absolute Contraindications to Exercise:
    - A recent significant change in the resting ECG suggesting significant ischemia, recent myocardial infarction (within 2 months) or other acute cardiac event;
    - Unstable angina
    - Uncontrolled cardiac dysrhythmias causing symptoms or hemodynamic compromise
    - Symptomatic severe aortic stenosis
    - Uncontrolled symptomatic heart failure
    - Acute pulmonary embolus or pulmonary infarction
    - Acute myocarditis or pericarditis
    - Suspected or known dissecting aneurysm
  - Relative Contraindications to Exercise:
    - Left main coronary stenosis
    - Moderate stenotic heart disease
    - Electrolyte abnormalities (e.g. hypokalemia, hypomagnesemia)
    - Tachydysrhythmia or Bradydysrhythmia
    - Hypertrophic cardiomyopathy and other forms of outflow tract obstruction
    - High degree atrioventricular block
    - Ventricular aneurysm
- Uncontrolled cardiovascular disease or hypertension
- Current total knee replacement is a revision

- Unable to walk 50 meters without an assistive device and to comfortably bear weight on the surgical knee
- History of muscular or neurologic disorder that may affect lower extremity function (e.g. muscular dystrophy, Parkinson's disease, multiple sclerosis)
- Participates in structured exercise more than twice a week
- Has a terminal illness
- Plans to have another total joint replacement in the lower extremities during study period
- Plans to relocate outside the immediate area during study period
- Refuses to participate in study protocol

4.2. Telephone Screening Process and In-person Eligibility Screening Visit Scheduling

All subjects who call to inquire about the study are asked for verbal consent to undergo telephone screening. The telephone screening is performed by the research staff of the study and contains questions regarding subject's demographic information, medical history and functional questionnaire (WOMAC-PF) that comprises eligibility criteria.

Research staff follows the telephone screening form (see **APPENDIX: A. Study Forms**: Telephone Screening form).

When a subject is eligible to be in the study, the research staff informs the research coordinator, who then schedules an in-person visit to perform the final screen and determines if the subject meets eligibility criteria to participate in the study.

The tentative appointment is confirmed with the physical therapist who will also evaluate the subject during the baseline visit in case eligibility is confirmed. The research coordinator then prepares an in-person eligibility screening/baseline mail package to be sent to subjects in preparation for the visit. The mailed package contains a copy of the informed consent document, driving directions to get to the research facility and a letter with general guidelines for the entire visit.

Prior to the in-person eligibility screening visit taking place, the research coordinator requests and obtains a medical release form from the surgeon for the subject to participate in the study. Additional medical releases (from a cardiologist and/or primary care physician) may be required in cases when subject answers any 'Yes' to the following questions on the telephone screening:

1. Has your doctor ever said that you have a heart condition and that you should only do physical activity recommended by a doctor?
2. Do you feel unreasonably out of breath?
3. Do you experience dizziness, fainting, or blackouts?
4. Do you feel pain in your chest when you do physical activity?
5. In the past month, have you had chest pain when you were not doing physical activity?
6. Is your blood sugar often above what doctor recommended?
7. Do you feel shaky, confused, or dizzy when you exercise?

The appointment is confirmed with the subject one day prior to the scheduled visit. The research coordinator must contact the subject to verify if the subject has any questions about the study (after reading the informed consent), remind subject to bring an updated list of medications, discuss transportation and directions, and re-confirm or reschedule the appointment.

4.3. In-person Eligibility Screening

At the in-person eligibility screening visit, the research coordinator greets and guides the subject to a clinical examination room. There, the research coordinator explains the study to the potential subject and clarifies any questions the subject might have. If the subject has no questions or after addressing the subject's concerns, both the coordinator and the subject sign 2 copies of the informed consent (1 signed copy is given to the subject and the other one is kept in a locked research file).

All subjects are asked to complete a Contact Information Form (see **APPENDIX: A. Study Forms**: Contact Information form) that is used for compensation purposes (i.e., to assign a pre-paid card - WePay System- to the subject). This form is stored in a locked file, separate from research data, to prevent breaches of confidentiality. The research coordinator logs-in the study web system and enters the subject's initials. When subject's initials are submitted, the database generates the study subject ID. The convention chosen for the subjects' IDs is the acronym of the study KTX followed by 3 digits (e.g. KTX001, KTX002, etc.)

Prior to completing the inclusion/exclusion criteria smart form, the database requires confirmation that the subject signed the informed consent form followed by the respective date in which the consent was obtained. This is a required step in the electronic database to certify that the study is in compliance with the University of Pittsburgh Institutional Review Board. Once the information is recorded the database, this allows access to study data collection forms.

The following forms are completed in the order provided below to confirm subject's eligibility (**APPENDIX: A. Study Forms**):

1. Demographics Form: demographical information is recorded in this form.
2. Medical History Form: records subject's body mass index (BMI in kg/m²), systolic and diastolic blood pressure, information about subject's TKR surgery (surgery date, TKR side, surgical technique), other total joint replacement in the lower extremities, medical conditions that affect subject's mobility, information on the standard rehabilitation the subject received after the TKR, if subject is engaged in supervised exercise and the type of exercise, and the subject's expectation on study's exercise programs.
3. Cumulative Illness Rating Scale: measures the severity of the subject's comorbidities.[38-41].
4. Medication Form: records the name and dosage of the medications taken by the subject to improve knee symptoms.
5. The Western Ontario and McMaster Universities Osteoarthritis Index (WOMAC): This form assesses the level of pain, stiffness and physical function while performing daily activities. This form was developed for people with hip and/or knee osteoarthritis. This is the primary outcome measure of the study.[42-45]
6. Inclusion/Exclusion form: inclusion and exclusion criteria are checked. Specific information that is collected in previous forms (e.g. unilateral TKR – "YES" or "NO" collected in the Medical History form)

are auto-populated from the respective forms. Criteria that are not collected from previous forms are asked to subject and entered manually by the research coordinator. Submission of this form establishes the subject's eligibility.

At any point during the screening process if a subject is deemed ineligible the evaluation is terminated and the subject is compensated for his/her time (see Section **14. STUDY COMPENSATION**).

If the subject is deemed eligible for the study, the research coordinator introduces the subject to the tester who continues onto baseline evaluation component of this visit.

5. BASELINE TESTING

5.1. Self-Reported Measures:

The baseline visit usually occurs on the same day as the in-person eligibility, unless subject requests the continuation to occur in a different day or in case there is a health concern (e.g. blood pressure above 170 x 110 mmHg) that would require a medical release from the primary care physician or other specialist. In this case, the continuation of the baseline visit is then scheduled after proper medical release is obtained.

The study tester provides the instructions and necessary assistance to subjects to complete the self-reported forms. (**APPENDIX: A. Study Forms**)

- Community Healthy Activities Model Program for Seniors (CHAMPS): measure self-reported physical activity. The CHAMPS questionnaire is formatted so that specific activities are listed on the form. Subject reports the activities he/she did in a typical week during the last 4 weeks, the number of times in a week he/she did the activity and the total amount of hours usually spent in that activity.[46]
- RAND-36: measures self-reported quality of life.[45]
- Arthritis Self-Efficacy Scale (ASES): this form has 3 subscales that are scored separately: pain, function and other symptoms subscales.[47,48]
- Falls History form: measure the number of falls the subject had in the past year.[49]
- Center of Epidemiologic Studies Depression Scale (CES-D10): measure depression symptoms.[50,51]
- Beck Anxiety Inventory: measure anxiety symptoms.[51,52]
- Tampa Scale for Kinesiophobia: 17 items scale that measures fear related to exercise activity.[53-56]
- Coping Strategies Questionnaire: measure self-reported coping strategies related to pain.[57-58]
- EQ-5D: measure general health status.[59]
- Canadian Occupational Performance Measure (COPM): measure self-reported performance and satisfaction of daily tasks that are deemed important to the subject.
- Patient-Reported Outcomes Measurement Information System (PROMIS) Physical Function & Pain Interference: questionnaire administered via Computerized Adaptive Testing (CAT). It measures self-reported physical function and self-reported pain interference during daily and social activities. (REF)

5.2. Clinical Examination

The Clinical Examination procedures are conducted by the tester. The results of each test are recorded on a hard copy of the Clinical Examination form (**APPENDIX: A. Study Forms**: Clinical Examination form)

and immediately transposed into the database. The database is set up with possible ranges for the results of each test to avoid typos while recording data.

- Clinical Examination procedures:
    1. Knee range of motion:
        a. Passive knee flexion: measured with a standard goniometer while the patient is lying in supine on an examination table. The knee is flexed its end range and value is recorded in degrees. This test is performed on both knees. Possible ranges: 50 to 150 degrees.
        b. Passive knee extension: measured with a standard goniometer while subject is lying in supine. An 18.5cm diameter round cushioned bolster is placed under the ankles so passive extension can be measured. In case the subject has a flexion contracture the value in degrees should be marked down as negative. If the subject has hyperextension, the value should be marked down as positive. This test is performed on both knees. Possible ranges: -20 to 20 degrees.

    2. Knee extension lag: determines presence of knee lag or not. Knee extension is measured actively and passively. Presence of knee lag is established if subject has a difference of 5 or more degrees between the two measurements.

    3. Single-leg balance test: subject is asked to be on single leg support while keeping the hands of the hips. The test lasts up to 60 seconds and it is stopped if the swing leg touches the floor, support foot moves on the floor, or arms swing away from the hips. Three trials are performed on each lower extremity. A stop watch is used to record the duration of each trial.[59]

    4. Repeated Chair Stand Test: tester records the number of times the subject is able to stand up from the chair without help. Subject sits on a standard chair without armrests with arms crossed in front of their chest. Subject rises to a full upright position repeatedly for 30 seconds while the tester counts the number of times subject did it. In addition, the tester records the time (in sec) it took for the subject to do 5 chair stands using one stopwatch. In case subject cannot stand from the chair safely, the test is not completed and this information is recorded.[60]

    5. Walking Ability Tests:
        a. Timed up and go test: subject sits on a standard chair (with armrests). The test consists on the subject getting up from the chair, walking to a pre-marked line 3 meters away, turning and sitting on the chair again. Time (in sec) is recorded in seconds using a stopwatch. Subject can use walking aid if required.[60,61]
        b. Time to walk 4-meters: measures self-selected gait speed (in m/sec). Time is recorded in seconds while subject walks 4-meter distance. [60]
        c. 40-meters Fast-Paced Walk Test: measures subject's ability to walk fast over short distances. Time is recorded while subject walks 40-meter distance as fast as he/she can. Ten-meter distance is marked on the floor, with cones placed 1 meter before and after the marks. Subject walks the distance, going around the cones 2 times. Assistive devices are allowed and recorded if used.[60]

d. 6 Minute Walk Test: measures aerobic capacity over long distances (in m). The total amount of laps walked on the outer part of the track, during 6 minutes, is counted and multiplied by 37.56 meters (1 lap). Additional meters walked are added to that value. Assistive devices are allowed and recorded if used. Subjects can sit down, if needed, while the stopwatch continues running.

6. Stair climbing test: measures the time (in sec) to go up and down 1 flight of stairs (11 standard height of 17 cm). One side of the handrail is used for safety purposes. Subject goes up and down the steps as fast as he/she can, without putting themselves at risk. The time to go up and the total time (time to go up and down) are recorded using 2 stopwatches. The time to go down is automatically calculated in the database.[61,62]

7. Sitting/Rising Test: measures subject's ability to sit and rise from the floor. Subject sits down on the floor using as minimal support as possible. The number of supports used (hands, forearms, knees, side of leg and/or of the foot) is recorded. Subject rises from the floor to a standing position, while tester records the number of support used and the number of unsteadiness (partial loss of balance). One practice trial is done before the test, and tester can provide instructions to improve subject's performance. In case subject refuses to do this test or tester deems unsafe, test is not completed and this information is recorded.[63]

8. Muscle Strength Tests:
    a. Hip Abductors Strength Test: measured while the patient is side lying. The tester uses a measuring tape to measure the moment arm, which is the distance in centimeters between the greater trochanter and 1 inch above the lateral epicondyle. The hip is placed in slight abduction and extension while the knee extended. A hand held dynamometer is placed at the line (1 inch above the lateral epicondyle) and the subject is asked to push up against the dynamometer as hard as he/she can for 5 seconds. Tester is trained to match the force produced by the subject, in other words, the tester cannot exert more force than the subject and "break" their maximum voluntary contraction. The force (in kg) produced is recorded in the database. Verbal encouragement is given during the test, and the test is performed on both lower extremities.[64]
    b. Quadriceps Strength Test: is measured bilaterally using a maximum voluntary isometric contraction (MVIC) of the quadriceps muscle. Subject sits on an isokinetic dynamometer (Biodex System 4 Pro) with the dynamometer force sensing arm secured to the ankle. The knee being tested is positioned in 70 degrees of flexion. The tester fasten subject's body with belts across the chest and hip while sitting on the chair to assure body stability and avoid compensatory muscle force from other muscle groups. The subject is asked to exert as much force as possible while extending the knee against the force sensing arm of the dynamometer. Subject performs 3 warm-up trials: one at 50%, one at 75% and one last at 100% of the maximal voluntary isometric strength. Five trials at 100% MVIC are performed with 1 minute rest for recovery in between trials. Data is

processed on a later date by a research assistant. The MVIC is measured in *newton-meter* (Nm). The highest value of each curve sustained for at least 3 time points is recorded as the MVIC for that curve. Three of the five trials with the highest values are recorded into the database.[64,65]

9. Real-time physical activity (SWA monitor): measures physical activity in real-time. At the end of the baseline testing session, a clean monitor and armband is given to subjects to be worn on the back of the right arm for 8 days. Subjects are instructed to remove the monitor during sleep and during water activities (e.g. shower, swimming). Subject also receives a daily log, instructions form (**APPENDIX: A. Study Forms**: Physical Activity forms) and a pre-stamped envelope with 1 additional AAA battery to return the monitor to the research team. Subject signs the receipt form, where monitor serial number is written.[36,37]

The in-person eligibility screening and baseline visit are estimated to last approximately 3.5 hours.

6. RANDOMIZATION

The study coordinator performs the randomization through a web-based computer system at the end of the baseline visit, thereby preserving allocation concealment. Once group assignment is established, the coordinator discloses this information to the subject followed by instructions on how to proceed with the study. Subjects randomized to either the clinic-based or the community-based exercise programs are scheduled for the first exercise visit only after physical activity data is collected (~8 days).

Patients are randomized using a 2:2:1 allocation ratio to receive one of the two exercise interventions as compared to usual medical care. This study uses an adaptive randomization approach with minimal sufficient balance algorithm [(66,67)] to minimize imbalances in important prognostic variables at baseline including gender, age, BMI, physical function, and knee range of motion. These measures have been selected due to their strong associations with the study outcomes of physical function and activity. Allocation is assigned based on the instantaneous imbalances instead of being generated as a fixed list prior to the beginning of the trial.

7. MASKING

While the treatment assignments clearly cannot be masked to the patient, several steps are taken to decrease bias: (1) Subjects are masked from in-depth information of intervention in the other group and are instructed not to discuss any aspects of the treatment with the testers; (2) The treating physical therapist and the leader of the group exercise are masked to subjects' performances on outcome measurements; (3) The testers are masked to subjects' group assignments. Despite the efforts to keep tester unmasked, the research team acknowledges that break of blinding may occur and a protocol deviation is completed reporting a break of blinding when that happens. To ascertain if the testers are kept masked throughout the study, at the end of the study the tester will try to guess group assignment.

8. STUDY INTERVENTIONS

8.1. Clinic-based Individual Outpatient Rehabilitative Exercises

The exercise program used in this group has been shown to be safe and feasible and combines the best research evidence. Subjects participate in 12 supervised sessions of exercise (60 minutes each) followed by a home exercise program. The 12 sessions are supervised by a physical therapist during 3 months in the following schedule: 2 sessions per week during weeks 1-3; 1 session per week in weeks 4 to 7; and 1 session every 2 weeks for the last two visits. This gradual weaning is designed is allow enough time for the subjects to learn the exercises and increase adherence with the home exercise program. Subjects are instructed to start home exercise after the 3rd week of the supervised program in a way that they exercise twice a week (either supervised exercise in the clinic or at home) during the 3-month intervention phase.

Treatment sessions utilize a pragmatic approach and include: (1) warm-up with stretching of lower extremity muscles and range of motion exercises; (2) moderate to vigorous intensity strengthening exercises of the major lower extremity muscle groups (knee extensors, knee flexors, hip extensors, and hip abductors); (3) moderate intensity aerobic training using a treadmill or exercise bicycle; (4) functional activities such as getting up from and sitting down in a chair, squatting, walking in place, kneeling, stair climbing and dancing; and (5) agility and balance exercises. All 5 components of the exercise program are used with each subject because patients with physical limitations post-TKR are all affected to varying degrees by these impairments. Exercises are performed in both legs and are initially performed at low intensity and progressively increased to the target level, as long as subjects do not experience increased pain, effusion, or decreased range of knee motion. Treatment sessions utilize a pragmatic approach, allowing the physical therapist to make modifications accordingly to subjects' needs. Individualization of exercise occurs in the selection of what exercises are emphasized in each component and the rate of exercise progression.

8.2. Community-based Group Exercise

Participants randomized to this group attend 45-60 minutes group exercise classes for older adults at local community senior centers at the same frequency/duration as the clinic-based exercise group; 2 times per week for 3 months. The size of group exercise classes is variable but generally larger than 4 participants. The research participants attend classes along with non-research participants who are members of the community centers. The community senior centers participating in this study are the Jewish Community Center (JCC) in Squirrel Hill, and the Vintage Senior Center. In these centers, there are target classes that have the same elements targeted by the physical therapist in the clinic-based exercise group (strengthening, balance and coordination exercises). The target classes at JCC are the SilverSneakers Circuit classes, at Vintage are the Enhanced Fitness classes. The classes consist of a variety of exercises designed to increase general muscular strength, improve cardiovascular fitness, joint mobility, balance, and daily living skills. No specific body region is targeted with these exercise classes. Some of the exercises include: partial squats, leg and knee extension/flexion, elastic tubing or free weight for strength training of the upper arm and chest muscles, coordination drills with a gym ball such as bouncing, throwing and catching, and low-impact cardiovascular exercise using treadmill, bikes or aerobic series on the floor. The classes are taught by trained physical fitness instructors. Subjects randomized to this group are

allowed to do the activities provided in the center they chose (i.e., participate in other classes, have lunch and other social events, use the fitness center), and the activities are tracked by the research personnel.

8.3. Usual Medical Care (waited-list control group)

The usual medical care group does not receive any attempt from the research team in a way that would interfere with their activities up their 6 months follow-up visit. At this visit, subjects randomized to clinic-based individual outpatient rehabilitative exercise group or to the community-based group exercise and will be exercising for 3 months.

9. EXERCISE COMPLIANCE

The exercise procedures of the study are being monitored by the research team on a regular basis through phone calls. Every month and a half, the research team contacts the subjects to track their compliance with the exercise program, collect information on additional exercises done outside the ones proposed by the study, and on adverse events. This information is recorded using the Health Status Update form in the database.

For the individualized outpatient rehabilitative exercise group, exercise is monitored using the daily logs completed by the physical therapist. Subjects are also asked to complete the home exercise log form, where they mark the exercises done at home. For the community-based group exercise, the research team contacts the community centers participating in the study to get a monthly report of subjects' attendance (electronic swipe of the center's cards, and a copy of the class signing sheet – at the JCC.

10. FOLLOW-UP PHONE-CALLS

The phone calls target dates are calculated to take place at 1.5 months (45 days), 4.5 months (105 days) following the original randomization date. The waited-list usual care group has a follow-up phone call 45 days after the second randomization date. The window of time to have these phone calls is 7 days prior or after the target date. The goal of these calls is to keep the subjects engaged in the study. [68,69] During the phone calls, the coordinator obtains information on health status, adverse events and co-interventions (**APPENDIX: A. Study Forms**: Health Status Update form). Exercise compliance is also monitored during these phone calls with questions regarding frequency and type of exercise that subjects are doing and it is recorded on the health status update form.

Subjects are asked by the research coordinator to contact the research personnel in case any of any change in health status occurs during their participation in the study.

11. FOLLOW-UP IN-PERSON TESTING

The target date to schedule the follow-up visits are calculated to take place 90 days (3 months visit) and 180 days (6 months visit) after the date of randomization. There is also an additional follow-up visit for the Usual Medical Care group 270 days (9-month visit) after the randomization date. The ideal window of time for the follow-up visits to take place is 7 days prior or 21 days after the target date. The trial coordinator attempts to contact subjects prior to the opening of the window to ensure that the visit is successfully scheduled within the specified 4-week window.

During the scheduling call, the subject is asked to bring an updated list of the medications (including the ones over the counter), reading glasses if needed, and exercise clothes. Subjects in the individualized physical therapy group are instructed to bring the home exercise log to this visit.

11.1. Three and Six Months Testing Sessions (and Nine Months for the Usual Medical Care Group)

The follow-up visits are estimated to last approximately 3.5 hours.

The trial coordinator greets the subject and walks him/her to the clinical evaluation room. During the initial part of this visit, the research coordinator is responsible for completing the following forms:

- Health Status Update Form: measures health status, adverse events, exercise program compliance, and co-interventions.
- Global Rating of Change: this form measures subjects' overall knee condition from the time they started the research study (baseline) to the current time point.
- Medication form: this form tracks any changes in subjects' medication list since enrollment in the study.

During the second part of the visit the tester is responsible to instruct the subject on how to complete the following self-reported measures:

- WOMAC
- CHAMPS
- RAND 36
- ASES
- Falls History form (same version as the one completed during the phone calls at 1.5 months)
- CES-D10
- Beck Anxiety Inventory
- Tampa Scale for Kinesiophobia
- Coping Strategies Questionnaire
- EQ-5D
- COPM
- PROMIS Physical Function & Pain Interference

Once the self-reported information above is completed, the tester starts to perform the following tests and record onto the clinical examination form:

- Passive knee flexion and extension, knee extension lag, single-leg balance test, time up and go test, repeated chair stand test, stair climbing test, time to walk 4-meters, 40-meter fast-paced test, 6-minute walk test, sitting/rising test, and muscle strength. During the clinical examination test, the tester measures the subject's height, weight, and blood pressure. If the tester deems any of tests unsafe for the subject to perform, the test is not completed and this information is recorded. In case the subject did not perform the strength tests in the baseline visit, he/she should not do it in the follow-up visits.
- Real-time physical activity (SW monitor): an activity monitor is given to the subject at the end of the visit. Instructions are the same as in the baseline visit.

At the end of this visit, the research coordinator reminds the subject about upcoming time point according to group assignment:

- Individualized physical therapy: subjects are instructed to continue the exercises learned in the sessions at home for the next 3 months. A new home exercise log is given to them so the research team can track their exercises.
- Community group exercise: subjects are instructed to continue attending the exercises classes during the next 3 months.
- Usual care wait-listed group: subjects are instructed to continue their regular activities up to their 6-month follow-up visit.

During this final part of the visit, the trial coordinator loads the subject's WePay card to reimburse for travel and time. A receipt is printed for the trial coordinator's records and a receipt with the subject's name is printed and given to the subject for his/her records.

11.2. Wait-list Usual Care group at the 6 months visit

At the end of the 6 month testing session, subjects in the wait-list usual care group are randomized to one of the two exercise groups: 1) individualized outpatient rehabilitative exercise group; or 2) community-based group exercise. The trial coordinator checks if the subject's health history has changed in the past 6 months to ensure continued eligibility. If the subject continues to be eligible to exercise, the trial coordinator gives him/her instructions to continue for the next 3 months according to the group to which they were randomized.

The second randomization (for the wait-list group only) follows the same procedures described above. Based on the second randomization date, these subjects have one follow-up phone call (45 days) and one follow-up testing visit (90 days).

12. CLINICAL MEASURES

12.1. Outcome Measures

Table 1 describes the clinical measures completed at each time point.

12.1.1. Primary Outcome Measure

The primary outcome measure is physical function at the 3-month follow up assessed by a patient-reported survey, the WOMAC-PF. The WOMAC-PF consists of 17 items related to physical function. Each item is scored on a 5-point Likert-type Scale with descriptors from 0-4 (none, mild, moderate, severe and extreme). The WOMAC PF is calculated as the sum of the items, for a maximum total score of 68. Higher scores indicate worse functional limitations. Reliability and validity of this instrument have been established.[42–44]

12.1.2. Secondary Outcome Measure

Secondary outcomes of physical function include a battery of performance-based tests that include: Self-Secondary outcomes of physical function comprise a battery of performance-based tests that include: Gait speed assessed by the 40-meters fast-paced walk test; Chair rise test that times participants during 5 repetitions of rising to a full upright position and sitting back down in the chair (18" chair without armrests) without assistance; Single leg stance test that records the time of balancing on one leg while keeping the hands on the hips. The test lasts up to 60 seconds and is stopped if the swing leg touches the floor, support foot moves on the floor, or arms swing away from the hips; Stair ascend/descend test that times participants while climbing up and down a set of 11 stairs (30 cm depth, 17 cm height) using a handrail on the preferred side; Six min walk test that assesses the distance covered while walking during 6 min on an unobstructed, rectangular circuit (marked in meters)[60-62]; Sitting-rising test that assesses the ability of participants to sit and rise from the floor.[63] Results of these tests are combined using a composite score formed with unit-weighted z scores of constituent tests to provide a more stable measure of the subject's underlying functional performance.

Additional secondary outcome includes physical activity measured using the SenseWear Minifly (SWM) (Body Media Inc, Pittsburgh PA) and the Community Healthy Activity Model Program for Seniors questionnaire (CHAMPS). The SWM provides real-time measures of physical activity in subjects' homes or communities during normal activities of daily life. The SWM has good reliability and validity. [37] Subjects are instructed to wear the SWM on the back of the left arm during wake time (they are asked to wear the monitor on the arm from the time they get up in the morning to the time they go to bed), except during shower and water activities. Moderate-intensity activities are recorded along with data captured on sedentary behavior and physical activity performed at light intensity (up to 3METs). The CHAMPS assess self-reported physical activity, and it is a reliable, valid and responsive instrument.[46] It assesses activities such as hobbies, work- and social-related activities, walking, swimming, dancing; and complements the information obtained from the SWM.

Table 1. Clinical measures completed at each time point.

Questionnaire/Form	Ba	Call 1.5 m	3 m	Call 4.5 m	6 m	Call 7.5 m	9 m
Demographics	X						
Medical History form	X						
Cumulative Illness rating scale	X						
Medication	X		X		X		X

WOMAC	X		X		X		X
Inclusion/Exclusion Criteria form	X						
CHAMPS	X		X		X		X
RAND 36	X		X		X		X
Arthritis Self-efficacy	X		X		X		X
Falls history	X	X	X	X	X	X	X
CES-D 10	X		X		X		X
Beck Anxiety Inventory	X		X		X		X
Tampa Scale for Kinesiophobia	X		X		X		X
Coping Strategy Questionnaire	X		X		X		X
COPM	X		X		X		X
Clinical examination form	X		X		X		X
Physical Activity Data	X		X		X		X
EQ-5D	X		X		X		X
PROMIS Physical Function & Pain Interference	X		X		X		X
Global Rating change form			X		X		X
Health Status Update		X	X	X	X	X	X
Attrition			X		X		X
Adherence to Intervention		X	X	X	X	X	X
Adverse Events		X	X	X	X	X	X
Co-interventions		X	X	X	X	X	X

12.1.3. Other Measures

At baseline, data are collected on demographics and biomedical characteristics and comorbidity. These data are used to characterize the sample. Demographics and biomedical characteristics include age, gender, race, education, BMI, self-rated health (excellent, good, fair, poor, or bad), discharge placement, number of prior rehabilitation sessions, surgical technique, and surgeon experience. Comorbidity is assessed by the Cumulative Illness Rating Scale (**APPENDIX: A. Study Forms**).

Additionally, data on medication, psychosocial factors, and impairments of the lower extremities are collected at baseline and each in-person follow-up visit to test potential predictors or modifiers of treatment response. Medication information includes medication prescribed and over-the-counter used for pain. Psychosocial Factors include fear-avoidance beliefs, anxiety, self-efficacy, depression, and pain coping. Lower extremities impairments knee pain, knee range of motion, and muscle strength (described in Section **5.BASELINE TESTING**).

Safety and exploratory outcomes include the measures of harm assessed by adverse events and measures of study engagements including attrition, adherence to intervention, and participation in co-interventions, respectively. Adverse Events include, but are not limited to, changes in knee symptoms, falls, hospitalizations, and TKR on the other knee. Attrition is defined as the number of patients dropping out of the study in each group. Adherence to intervention is estimated by the proportion of sessions attended in each group and the proportion of patients missing each session. Co-Intervention is defined as additional treatment sought besides the ones prescribed by the study.

13. ADVERSE EVENTS

13.1. Management

The occurrence of adverse events is monitored for each subject on an ongoing basis throughout the study. Reporting of adverse events in the context of the proposed program of research occurs according to the following definitions:

- Serious. This adverse event is fatal or life-threatening; requires hospitalization, or produces a disability.
- Moderate or greater severity. This adverse event requires medical evaluation and/or medical treatment; or is a serious adverse reaction.
- Unexpected. This event is not identified in nature, severity or frequency in the IRB-approved research protocol or informed consent document.
- Associated with the research intervention. There is a reasonable possibility that this event may have been caused by the research intervention (i.e., a causal relationship between the event and research intervention cannot be ruled out by the investigators).

13.2. Report

All adverse events that are (a) unexpected; (b) of moderate or greater severity; and (c) associated with the research intervention are reported to the IRB. In the case of a serious adverse event, an emergency meeting of the investigative team is called. At the time of this meeting, a determination is made as to whether the trial should be prematurely interrupted. Expected adverse events; unexpected adverse events of minor severity; or adverse events which are determined by the PI to be unrelated to the research intervention are not reported to the IRB. These events are reported to PCORI during the annual report.

All adverse events are reported according to the following timeline: If the event is fatal or life-threatening, the report to the IRB and the PCORI occurs within 24 hours of the event. If the event is unexpected, and of moderate or greater severity (but not fatal or life-threatening), and associated with the research intervention, it is reported to the IRB and the PCORI within 10 calendar days of the reaction. The IRB and the PCORI are also notified as soon as possible of major disputes between the PI and/or project staff and a research subject or between research investigators (including research staff) involved in the proposed program of research if the resolution of the dispute is or will be problematic. If an unexpected adverse event occurs, the PIs re-assess the risk/benefit ratio of the study and submit any modifications deemed necessary to the IRB and the PCORI for approval.

14. STUDY COMPENSATION

This study is using the UPMC WePay System (https://wepay.upmc.com/WP/) to compensate subjects for their participation. Staff online training is required.

During the baseline visit, the coordinator searches for or adds subject information to the WePay System. Subjects need to be registered in the WePay system so compensations can be processed. Once the subject is registered in the system, the coordinator assigns one pre-paid WePay card to the subject (need card verification

value information and 4-digit PIN) and loads US$40.00. Two receipts are printed. The one with subject's name is given to the subject. The other, with subject's study ID, is kept by the research team.

Subjects were compensated for their time at all testing sessions (baseline, 3 and 6 months testing visits).

15. DATA ANALYSIS AND SAMPLE SIZE JUSTIFICATION

15.1. Primary Hypothesis

The primary hypothesis is that subjects in Groups 1 and 2 will demonstrate better physical function and physical activity as compared to Group 3 (usual medical care). Analysis for this hypothesis will use an intention-to-treat approach. The primary outcome for this analysis is the WOMAC-PF subscale at 3 months. This analysis will use contrasts from a linear mixed models analysis for 3 and 6-month function controlling for baseline function and the randomization covariates (age, gender, BMI, physical function, ROM). We will first explore the intervention by time interaction and then proceed to a main effects model with only group and time. Our primary interest is the 3-month comparison between the clinic-based individual outpatient exercise and the community-based exercise groups. The linear mixed models allow maximization of the number of individuals used for the analyses, as a person can contribute information at both time points, or just at one time point. To test if the improvements in outcomes are sustained, we will use contrasts from the linear mixed model at 6 months. For the secondary outcomes of physical function (battery of performance-based tests such as walking ability, chair rise, single leg stance, stair climbing, six-minute walk, and sitting-rising) and physical activity, analyses are performed as described above, one for each measure. We will combine the score of the performance-based tests using a composite score formed with unit-weighted z scores of constituent tests to provide a more stable measure of the subjects' underlying functional performance.[68]

Sample size and power calculations for primary analysis were based on the primary endpoint of WOMAC-PF subscale at 3 months. We propose to recruit 240 subjects (96 in each exercise arm and 48 in the usual care arm) to allow approximately 86 subjects in each exercise arm and 43 in the usual care arm available for a complete case analysis (assuming 10% attrition at 3 months). With an alpha level of 0.05, 2 tails test, a sample size of 172 (n=86 in each exercise group) will provide 81% power to detect a difference of 3.3-point difference between the two exercise groups in WOMAC-PF (SD of 7.7)[35]. The sample size of 43 in the usual medical care group will provide 80% power to detect a difference of 5.2-point difference in WOMAC-PF between the usual medical care group and any exercise group. Power analysis was conducted in NCSS/PASS (PASS 12 Power Analysis and Sample Size Software (2013). NCSS, LLC. Kaysville, Utah, USA, ncss.com/software/pass).

15.2. Secondary Hypothesis

The secondary hypothesis is that a group of baseline biomedical and psychosocial measures will be associated with treatment response. For this analysis, each subject will be classified as a responder or non-responder based on a minimum change score of 20% in both the WOMAC-PF and the composite score of functional performance at 3 months, thus yielding a binary outcome. Baseline variables will be summarized separately for responders and non-responders. Unadjusted odds ratios will be estimated using univariate logistic regression. To consolidate potential predictors, we will test for collinearity among baseline variables that are associated with

the response. Baseline measures associated with response at the p<0.15 level in unadjusted models will be added to multivariable logistic regression models to assess predictors of treatment response. We will limit the number of predictors going into any one model to no more than one predictor per 10 responses (or 10 non-responses, whichever is less); if more variables are significant, the model will be limited to the most significant variables, after adjusting for those deemed a-priori to be clinically significant.[74]

Power calculation for the secondary analysis is based on the binary outcome of 20% change in physical function. Participants initially randomized to one of the exercise arms and those in the usual care group later randomized to the exercise arms will be included in the analysis for a total of approximately 200. If the expected response rate ranges between 50% and 60%, we would be able to detect an odds ratio of 2.2 to 2.4 with 80% power assuming a binary predictor with 50% split in the sample.

15.3. Exploratory Aim

For the exploratory aim, we will calculate dropout rates as proportions of subjects randomized and as a cumulative probability of remaining in the study using survival analysis techniques, such as the product-limit estimator. These statistics can be estimated at various times following randomization and take into account when dropouts occur. Descriptive statistics will be used for reporting and evaluating implementation of the exercise protocols including the proportion in attendance for each session and the average number of sessions attended by group. To assess the impact of non-adherence, we propose to explore using instrumental variable (IV) methodology to estimate the efficacy of our interventions in the presence of non-adherence.[70,71,72] We propose to use the two-stage IV methods which can be easily implemented using simple linear structural models for the effect of sessions attended on the primary outcome of function. We will also calculate the 6-month incidence (and 95% CI) of individual adverse events by organ system and relatedness to the study for each group. We will estimate the incidence of adverse events with a specific focus on those deemed definitely, probably, or possibly related to interventions. For adverse events, clinical judgments will be considered more important than statistical testing.

We also propose sub-group analyses to explore heterogeneity of treatment effects using several potential moderators of treatment response measured prior to randomization that may either potentiate or attenuate the effects of our intervention (e.g., patient gender, age, BMI, range of knee motion). These are the same prognostic variables used for the adaptive randomization in the study. We will examine interactions between the treatment and modifier being considered. Even if the interaction is not statistically significant, we would estimate the treatment effects stratified by age along with the 95% confidence intervals to look for consistency of treatment effects.

16. DROPOUT AND MISSING DATA

We estimate the attrition to be 10% at 3 months follow-up and 15% at 6 months. We will compare baseline characteristics between patients with and without the assessment at 3 and 6 months to assess potential biases in the complete case analysis. We will also try to obtain reasons for study drop-out to assess missing data mechanism (missing completely at random, missing at random, non-ignorable missingness). We will use several

missing methods for imputing data and re-analyze using intention-to-treat (as randomized) to assess the impact of missing data on our conclusions as recommended. [76] We will first use multiple imputations (with M=10 imputations) which assumes the data are missing at random. Since the data could be missing not at random, we will use another approach of assigning the lowest observed scores for missing values differentially by treatment group (non-ignorable missingness). The approach assumes the missingness is directly related to the value of missing data, i.e., the people who are missing data on function have worse function scores (did not come in for assessment because function was worse). Results of all approaches to missing data will be presented in the primary paper for our study. If our significance and interpretation of our treatment effect vary depending on the method of imputation, we will view any conclusion cautiously.

17. RECORD KEEPING

The majority of the forms and questionnaires used in this study are entered directly into the database. The hard copy of the forms completed on paper are being stored in subject's file and kept in a locked file cabinet in the trial coordinator's office. Only study personnel has access to these files.

Table 2 (below) shows the type of data storage for all forms. The telephone screening is completed in paper form, and only the recruitment source and the inclusion/exclusion criteria are entered in the database (without the subject's name and telephone number). Telephone Screening forms of subjects who are deemed eligible are kept in locked file cabinet separately from research data collection forms.

A copy of the Informed Consent signed by the subject and by the trial coordinator is kept in a separate locked file cabinet in the trial coordinator's office, as well as the Contact Information form.

Subjects bring an updated list of medication they take, including the ones they take over the counter, to all follow-up visits. This information is entered in the study database, but the hard copy is kept in subject's file.

The Inclusion/Exclusion criteria form is completed directly in the database. A copy of the complete form is printed and given to the PI to sign and date. This is done in order for the PI to be aware and to control only eligible subjects are randomized to the study.

The clinical examination form is completed in paper form and is immediately entered into the database by the tester. The hard copy is kept in subject's file.

The EQ-5Dis recorded in paper form. It is kept in subject's file.

Table 2. **Type of data storage.**

Form Name	Hard Copy – paper form	Electronic Entry
Telephone Screening form	X (used first)	X (entered later)
Informed Consent	X	
Contact Information form	X	
Demographics		X

Medical History form		X
Cumulative Illness rating scale		X
Medication	X (used first)	X (entered later)
WOMAC		X
Inclusion/Exclusion Criteria form	X	X
CHAMPS		X
RAND 36		X
Arthritis Self-efficacy		X
Falls history		X
CES-D 10		X
Beck Anxiety Inventory		X
Tampa Scale for Kinesiophobia		X
Coping Strategy Questionnaire		X
COPM		X
Clinical examination form	X (used first)	X (entered later)
PROMIS Physical Function & Pain Interference		X
Physical Activity Data		X
EQ-5D	X	
Physical Activity		X
Randomization		X
Global Rating change form		X
Health Status Update		X

The database is web-based for direct data entry, where subjects are identified by a study ID (i.e. KTX000), and no personally identifiable information is stored in it or used in any of the analyses.

18. DATA MANAGEMENT PLAN

Data management is overseen by the PI, Dr. Moore and Dr. Gil, and is coordinated by the Center of Research on Health Care Data Center (DC). The DC created an electronic System for Data Management (eSYSDM) for data collection, tracking, follow-up, reporting, and analysis need. The research coordinator obtains the patient's initials once a patient is recruited and deemed eligible for the study to initiate inclusion in the in the tracking system. The tracking system monitors enrollment and tracks follow-up rates and the data entry process, providing up-to-date status reports. The eSYSDM includes verifying the data, out of range data checks, and repeated evaluation of data process, eliminating the possibility of most incorrect entries and preventing extensive recoding and cleaning by the statistician.

The primary method of data collection is through the database. However, if access to the internet is disrupted, paper forms are available to ensure data collection. All data collected in paper forms are stored in the subject's research chart identified by their ID. In this case, coordinator contacts the database programmers via telephone to obtain the subject's group assignment.

19. QUALITY ASSURANCE

To ensure data quality and integrity we are using standard methods of data collection and recording, have formal staff workshops on research integrity, document computer operations and data editing procedures, and have regular meetings with project staff to review any changes in procedure. The electronic forms are maintained by the DC on a local network in a relational database. The DC performs routine data edit checks for consistency. Once data are edited, temporary files will be merged to generate the final files that will be used for data analyses. All files are backed-up daily and archived weekly.

Dr. Gil and the trial coordinator check all data collected every 3 months to certify that data is being collected and maintained properly. Equipment, such as the Biodex System, requires calibration. Research staff members are trained to calibrate the machine on a regular basis: the Biodex is calibrated every 3 month.

20. DATA SAFETY AND MONITORING BOARD

Study personnel decided to have a Data Safety and Monitoring Board (DSMB) to promote quality of monitoring the study. Since PCORI does not have its own DSMB Guidelines, this board follows the National Institute of Health (NIH) Guidelines.

The DSMB meetings occur every 6 months, and the reports are part of the annual reports the principal investigator sends to PCORI.

The DSMB reviews the accumulated study data for participants' safety, study conduct, and progress, and makes recommendations about study continuation, modification, or termination. The DSMB is responsible for defining its deliberative processes, including event triggers that would call for an unscheduled review, stopping guidelines, unmasking and voting procedures. The DSMB is also responsible for maintaining the confidentiality of its internal discussions and activities as well as the contents of reports provided.

A narrative summary and tables are compiled prior to each DSMB meeting and include the following:

Open Session:

Table 1A. Screening Information and Reasons for Ineligibility

Table 1B. Enrollment (Consented) and Randomized by Month of Study

Figure 1. Comparison of Target to Actual Enrollment by Month

Table 2. Reasons for Screen Failures

Table 3. Participant Enrollment and Status

Table 4. Demographic and Key Baseline Characteristics by Group

Table 5. Adverse Events: Level of Severity

Table 6. Adverse Event Details

Table 7. Serious Adverse Events

Table 8. Deaths

Table 9. Protocol Deviations

Table 10. Summary of Missed Visits

Table 11. Exercise Compliance

Closed session: Relevant data displayed by intervention arm. Masked research personnel is dismissed at this point of the meeting.

CONSORT Diagram (by intervention arm)

Table 12. Demographic and Key Baseline Characteristics (by intervention arm)*

Table 13. Adverse Event Details (by intervention arm)

Table 14. Protocol Deviations (by intervention arm)

Closed Executive Session: Only DSMB members to ensure complete objectivity as they discuss outcome results, make decisions, and formulate recommendations regarding the study.

After the meeting, the research team prepares the minutes to be approved by the DSMB. All reports and approved minutes are kept electronically in the study folder on the School of Health and Rehabilitation Sciences network system. DSMB members are compensated for the meetings.

DSMB Members:

- Julie Fritz, PT, PhD – Professor at the Physical Therapy/Orthopedic Surgery Operations, University of Utah (chair of the board)

- David Sinacore, PT, PhD – Professor at the Physical Therapy/Medicine, Washington University School of Medicine in St. Louis

- Margaret Conroy, MD, MPH – Assistant Professor at the Medicine, Epidemiology, Clinical Translational Science, University of Pittsburgh

- Subashan Perera, PhD – Associate Professor at the Medicine, Co-Director & Senior Statistician PEPPER CENTER.

21. <u>HUMAN SUBJECTS</u>

21.1. Institutional Review Board (IRB) Review and Inform Consent

This protocol and the informed consent document and any subsequent modifications will be reviewed and approved by the IRB (PRO14080261). A signed informed consent form is obtained from all subjects (**<u>APPENDIX</u> D. Informed Consent**). The consent form describes the purpose of the study, the procedures to be followed, and the risks and benefits of participation. A copy of the signed informed consent form is given to the subject.

21.2. Risks/Benefits Assessment

All evaluations are conducted for the purposes of the present research only. Research data comes from an in-person evaluation of subjects such as the history (e.g., demographics, biomedical factors, discharge placement, prior rehab), physical examination (knee range of motion and muscle strength), and research questionnaires (querying about their functional limitations, pain, physical activity) administered by project staff. Performance-based measures are used to collect information regarding lower extremity physical function. Real-time physical activity data is collected during a week in a free-living condition. While participating in this clinical trial the subjects are able to take their regular medications and therefore the proposed clinical trial will likely not affect the daily lives and the management of other medical conditions.

21.3. Potential Risks

The risks to the subjects are minimal. It is common for individuals to experience muscle or joint soreness in their lower extremities following functional testing or exercise intervention. This muscle or joint soreness typically occurs within 48 hours after physical activity but usually resolves within 1-2 days. Additional risks associated with exercise may include tripping and falling, or an exacerbation of the subject's knee pain and inflammation. Because subjects participate in aerobic exercises (treadmill walking or stationary bicycle), there is a rare risk that subjects may experience chest pain, dizziness, shortness of breath, or a heart attack.

21.4. Risk Management and Emergency Response

To reduce risks, all testing and treatments are administered by health professionals who monitor vital and clinical signs during the performance of exercises. To safeguard against the occurrence of injuries or falls, the exercises are performed under the close supervision of a physical therapist or leader of the group exercise class. To safeguard against the risk of a heart attack, we exclude subjects who have uncontrolled cardiovascular disease or hypertension and also subjects who have absolute or relative contraindications to exercise testing, as established by the American College of Cardiology/American Heart Association. To minimize the risk of muscle or joint soreness, subjects progress through study exercises only if they do not experience increased pain, joint effusion, or decreased range of motion. Signs and symptoms of knee inflammation are monitored during the study to determine if training activities exacerbate these conditions. Training activities associated with increased signs and symptoms of inflammation are suspended until symptoms resolve. If symptoms persist, the subject is referred to the study consultants or the patient's physicians. In addition, the exclusion criteria provide that individuals who are prone to falling or have progressive motor disorder will not participate in the proposed study. All subjects are informed of any potential risks prior to their participation in any study procedures and are told that they are free to withdraw from the study at any time. Although no other risks are anticipated, subjects

will be informed if any new information arises regarding risks of participation that may affect their decision to continue in the study. Emergency medical treatment for injuries solely and directly related to participation in this research study are provided by the hospitals affiliated with the University of Pittsburgh Medical Center (UPMC). It is possible that the hospital may bill the subject's insurance provider for the costs emergency treatment, but none of the costs will be charged directly to the subject.

21.5. Confidentiality

Patient confidentiality is maintained throughout the study. The risk of breaching subject confidentiality is minimized by using a web-based system of data entry. The data is directly entered into a computer at the time of the interviews. A relational database is stored on a local network where only select research team members have access. The Electronic System for Data Management elaborated in conjunction with the Data Center (http://www.crhc.pitt.edu/DataCenter), is stored on a local network where only select research team members have access to the database. All files are backed-up daily and archived weekly. The weekly data are stored in a safety deposit, off-site (> 1 mile off campus). The files are maintained for 1 year until the data are erased. All study subjects are assigned unique study identifiers that appear on all data collection instruments, tapes, documents, and files used in the statistical analysis and manuscript preparation. Only limited team members have access to personal information needed for tracking and informed consent. No personal information concerning study participants will be released without their written consent.

21.6. Potential Benefits of the Proposed Research to Human Subjects and Others

The potential benefits of this research include improvement in physical function and increase in physical activity after participation in the exercise program, and thus potential benefits on overall health. The potential benefits, therefore, outweigh the minimal anticipated risks to participants.

21.7. Importance of the Knowledge to be Gained

Our study is designed to test the hypothesis that exercise at later stage post TKR can overcome the functional limitations experienced by these patients. If these limitations are overcome, future disability may be prevented. Moreover, by increasing physical activity, our study may directly impact the general health of subjects following TKR. Additionally, this study will inform the management of subjects post TKR and the design of public health programs to extend the number of years free of disability in this population.

22. STUDY ORGANIZATION AND ADMINISTRATION

22.1. Administration/ Research Personnel

- Sara R. Piva, PT, PhD, Study Principal Investigator.
- James J. Irrgang, PT, ATC, PhD, Co-Investigator
    a. Liaison between the study personnel and the surgeon's practices
- Michael Schneider, DC, PhD, Co-Investigator
    a. Collaborates in developing the best practices to engage stakeholders into study implementation and dissemination

    b. Oversees the panel discussions to ensure conducting them in a manner that allows all voices to be heard

- Charity Moore, MSPH, PhD, Co-Investigator
    a. Biostatistician
    b. Responsible for overseeing randomization and all aspects of statistical analysis
- Alexandra Gil, PT, PhD, Co-Investigator
    a. Responsible for the coordination of DSMB meetings and Advisory Panel meetings
    b. Coordinates data management and processing between the PT-CTRC and the University of Pittsburgh Center for Research on Health Care Data Center
- Maria Beatriz Catelani, PT, MS, Trial coordinator
    a. Assists with recruitment
    b. Responsible for enrollment, scheduling of in-person eligibility/baseline and follow-up testing
    c. Tracks participants
    d. Participates in administering tests
- Gustavo Almeida, PT, MS, Trial coordinator
    a. Assists with recruitment
    b. Participates in administering tests (tester)
    c. Checks accuracy of data retrieved from Bodymedia Armband software for physical activity data
- Anthony DiGioia, MD, Consultant
    a. Consults any clinical issues that arise with the subjects undergoing research procedures covering both surgical and clinical aspects of subject's needs
    b. Helps with recruitment efforts
- Brian Klatt, MD, Consultant
    a. Consults any clinical issues that arise with the subjects undergoing research procedures covering both surgical and clinical aspects of subject's needs
    b. Helps with recruitment efforts
- Gwendolyn Sowa, MD, PhD, Consultant
    a. Consults any clinical issues that arise with the subjects undergoing research procedures covering both surgical and clinical aspects of subject's needs

23. STUDY PLACES

23.1. Community Centers

Two of the largest community centers in Pittsburgh are directly involved with this research study: the Vintage Senior Community Center, in the East Liberty section of Pittsburgh; and the Jewish Community Center (JCC) located in the Squirrel Hill. The community-based group exercise arm of this study are conducted at these two local community centers. Both centers are giving our research subjects access and short-membership to their facilities in order to participate in the group exercise classes that they offer to older adults. The Vintage and JCC executive directors have agreed to serve on the community stakeholder Advisory Panel, as well as the exercise instructors.

23.2. PT-CTRC

The Physical Therapy Clinical Translational Research Clinic (PT-CTRC) is serving as the central location for all testing sessions (in-person eligibility, baseline visit, and follow-up assessments) as well as for the treatment sessions for those subjects who are randomized to the individualized outpatient rehabilitative exercise.

24. RESEARCH ENGAGEMENT PLAN

This study involves several groups or stakeholders who are deeply engaged. We have assembled an Advisory Panel comprised of several stakeholders, each with different perspectives and areas of interest. We meet semi-annually, either in person or via phone conference, throughout the entire 3-year research timeframe. Members of the Advisory Panel have been and will continue to be engaged in order to provide input into the preparation, execution, and translation phases of the study as described below:

PREPARATION PHASE: Patients were involved through informal communication during research participation, structured interviews, and meetings to discuss study design. They have directly influenced the selection of comparators, outcomes, and study design; Providers provided input during study development and helped to shape the usual medical care arm. They provided key input to the individualized outpatient rehabilitative exercise arm. All providers supported the need to test the effectiveness of exercise at later stages after TKR and the inclusion of a community-based exercise group; the community groups provided input about the community-based group exercise that takes place at the community centers. They have been engaged with the development of the research design by allowing the PI to observe their group exercise classes for older adults, and to meet with the fitness instructors who teach these classes and older adult members of their organizations. The senior fitness instructors at these centers collaborated to develop pragmatic exercise protocols.

EXECUTION PHASE: Patients edited recruitment materials and did a trial-run of study procedures to ensure that the paperless system of data collection is age-appropriate and that the research personnel is well trained. They are also helping to spread the word about our study through their social media contact lists. Patients are instrumental in providing peer-information about the study for potential participants who would like to discuss study participation with someone who has been part of research studies. Patients are also part of a team of Patient Partners who interview subjects who have participated in the intervention arms of the study to collect information on their experiences and suggestions. The information collected during the interviews is key to shaping the delivery of interventions to improve the care and outcomes of patients who undergo TKR. Along with other lay members of the Advisory Panel they also give feedback on any potentially counterintuitive results. Providers, along with patients, were asked to provide input to maximize recruitment and retention, and are helping to interpret research findings from the stakeholder category to which they belong. Three prominent orthopaedic surgeons are actively engaged with the direct referrals of patients who have had TKR. The directors of the community centers, the JCC and Vintage Centers, forward information about our study through their membership email lists, e-newsletters/print newsletters, bulletin boards, and allow us to place informational brochures in their facilities. They also sponsor breakfast meetings where the PI will present information about the study. The community group representatives and payers are asked to provide interpretation of the results from public health, community policy, and health plan perspectives.

TRANSLATION PHASE: We plan specific steps to aid in the dissemination of the research results. Patients will be asked for their input on the development of lay summaries of the study results and will assist with the design and editing of informational booklets and pamphlets for patients who undergo TKR. Providers will help to facilitate presentations to disseminate the research findings at national meetings and conferences with their respective professional associations including the American Academy of Orthopedic Surgery, American Physical Therapy Association, and American College of Rheumatology. The payers will assist with dissemination of the research results to their network providers and work with the PI to organize regional meetings where the findings can be presented to clinicians. Community and advocate organizations will disseminate the research results through email newsletters to their members such as the community centers newsletter and the Arthritis Foundation Magazine.

All stakeholders will be compensated for their collaboration.

25. STUDY OPERATIONS

Any proposed changes to the protocol will be reviewed by the research team and DSMB, and recommendations will be approved by PCORI before implementation. Protocol changes made will be incorporated in the Manual of Operational Procedures (MOP) and Informed Consent document. A record of all changes, including rationale will be kept on file for future reference. Protocol Revisions will be tracked and will be inserted in the MOP, including previous information, the change made, who made the change, and the date the change was made and approved. On each page of the MOP, there will be a version number and date of approval to facilitate tracking the revisions.

26. PUBLICATIONS

Study protocol will be published at the end of the first year of the study in an open-journal such as BioMed Central Musculoskeletal Disorders. This will enable other researchers and funding agencies to see that this type of exercise trial post TKR is underway, reducing the duplication of research effort and potentially leading to future collaborations with other researchers interested in the same topic. It will also help researchers engaged in systematic reviews to find out our trial, which may reduce publication bias. Lastly, it will provide a mechanism for other researchers with similar research interests to contact the PI about gaining access to more specific research protocols. If the manuscript is not accepted for publication, it will be made available to other researchers upon request.

The investigators will pursue publication of the primary outcomes within 6 months after study completion. Authorship will be determined prior to writing the manuscript and will be based on the relative scientific contributions of the investigators and Key Personnel. All authors will review and approve the manuscript prior to submission for review.

All publications and presentations will be informed to PCORI within 30 days of submission and will include acknowledge of funding from Patient Centered Outcomes Research Institute (PCORI), CER-1310-06994.

27. REFERENCES

1. Kurtz, SM, Ong, KL, Schmier, J, et al. Primary and revision arthroplasty surgey caseloads in the United States from 1990 to 2004. J Arthroplasty. 2009; 24:195-203
2. Losina, E and Katz, JN. Total joint replacement outcomes in patients with concomitant comorbidities: A glass half empty or half full? Arthritis Rheum. 2013.
3. Stranges E, Russo A, and Friedman B. Healthcare Cost and Utilization Project (HCUP), Agency for Healthcare Research and Quality; Statistical brief #82: procedures with the most rapidly increasing hospital costs, 2004-2007. 2009.
4. O'Reilly, S, Jones, A, and Doherty, M. Muscle weakness in osteoarthritis. Curr Opin Rheumatol. 1997; 9:259-262.
5. Sharma, L. Proprioceptive impairment in knee osteoarthritis. Rheum Dis Clin North Am. 1999; 25:299-314, vi.
6. Sharma, L, Hayes, KW, Felson, DT, et al. Does laxity alter the relationship between strength and physical function in knee osteoarthritis? Arthritis Rheum. 1999; 42:25-32.
7. Slemenda, C, Brandt, KD, Heilman, DK, et al. Quadriceps weakness and osteoarthritis of the knee. Ann Intern Med. 1997; 127:97-104.
8. Do, BT, Hootman, JM, Helmick, CG, et al. Monitoring healthy people 2010 arthritis management objectives: education and clinician counseling for weight loss and exercise. Ann Fam Med. 2011; 9:136-141.
9. Murphy, L and Helmick, CG. The impact of osteoarthritis in the United States: a population-health perspective: A population-based review of the fourth most common cause of hospitalization in U.S. adults. Orthop Nurs. 2012; 31:85-91.
10. Jones, CA, Voaklander, DC, Johnston, DW, et al. Health related quality of life outcomes after total hip and knee arthroplasties in a community based population. J Rheumatol. 2000; 27:1745-1752.
11. George, LK, Ruiz, D, Jr., and Sloan, FA. The effects of total hip arthroplasty on physical functioning in the older population. J Am Geriatr Soc. 2008; 56:1057-1062.
12. Franklin, PD, Li, W, and Ayers, DC. The Chitranjan Ranawat Award: functional outcome after total knee replacement varies with patient attributes. Clin Orthop Relat Res. 2008; 466:2597-2604.
13. Konig, A, Walther, M, Kirschner, S, et al. Balance sheets of knee and functional scores 5 years after total knee arthroplasty for osteoarthritis: a source for patient information. J Arthroplasty. 2000; 15:289-294.
14. Walsh, M, Woodhouse, LJ, Thomas, SG, et al. Physical impairments and functional limitations: a comparison of individuals 1 year after total knee arthroplasty with control subjects. Phys Ther. 1998; 78:248-258.
15. Swanik, CB, Lephart, SM, and Rubash, HE. Proprioception, kinesthesia, and balance after total knee arthroplasty with cruciate-retaining and posterior stabilized prostheses. J Bone Joint Surg Am. 2004; 86-A:328-334.
16. Wada, M, Kawahara, H, Shimada, S, et al. Joint proprioception before and after total knee arthroplasty. Clin Orthop Relat Res. 2002;161-167.
17. Woolhead, GM, Donovan, JL, and Dieppe, PA. Outcomes of total knee replacement: a qualitative study. Rheumatology (Oxford). 2005; 44:1032-1037.

18. Beswick, AD, Wylde, V, Gooberman-Hill, R, et al. What proportion of patients report long-term pain after total hip or knee replacement for osteoarthritis? A systematic review of prospective studies in unselected patients. BMJ Open. 2012; 2:e000435.
19. Noble, PC, Gordon, MJ, Weiss, JM, et al. Does total knee replacement restore normal knee function? Clin Orthop Relat Res. 2005;157-165.
20. Levinger, P, Menz, HB, Wee, E, et al. Physiological risk factors for falls in people with knee osteoarthritis before and early after knee replacement surgery. Knee Surg Sports Traumatol Arthrosc. 2011; 19:1082-1089.
21. Naal, FD and Impellizzeri, FM. How active are patients undergoing total joint arthroplasty?: A systematic review. Clin Orthop Relat Res. 2010; 468:1891-1904.
22. Riddle, DL, Singh, JA, Harmsen, WS, et al. Clinically important body weight gain following knee arthroplasty: A five-year comparative cohort study. Arthritis Care Res (Hoboken). 2012.
23. Westby, MD and Backman, CL. Patient and health professional views on rehabilitation practices and outcomes following total hip and knee arthroplasty for osteoarthritis:a focus group study. BMC Health Serv Res. 2010; 10:119.
24. Rubenstein, LZ, Powers, CM, and Maclean, CH. Quality indicators for the management and prevention of falls and mobility problems in vulnerable elders. Ann Intern Med. 2001; 135:686-693.
25. Brach, JS, Vanswearingen, JM, Newman, AB, et al. Identifying early decline of physical function in community-dwelling older women: performance-based and self-report measures. Phys Ther. 2002; 82:320-328.
26. Guralnik, JM, Ferrucci, L, Simonsick, EM, et al. Lower-extremity function in persons over the age of 70 years as a predictor of subsequent disability. N Engl J Med. 1995; 332:556-561.
27. *2008 Physical Activity Guidelines for Americans* . 2008. U.S. Department of Health and Human Services.
28. Minns Lowe, CJ, Barker, KL, Dewey, M, et al. Effectiveness of physiotherapy exercise after knee arthroplasty for osteoarthritis: systematic review and meta-analysis of randomised controlled trials. BMJ. 2007; 335:812.
29. Fritz, JM, Hunter, SJ, Tracy, DM, et al. Utilization and clinical outcomes of outpatient physical therapy for medicare beneficiaries with musculoskeletal conditions. Phys Ther. 2011; 91:330-345.
30. DeJong, G, Tian, W, Smout, RJ, et al. Long-term outcomes of joint replacement rehabilitation patients discharged from skilled nursing and inpatient rehabilitation facilities. Arch Phys Med Rehabil. 2009; 90:1306-1316.
31. DeJong, G, Tian, W, Smout, RJ, et al. Use of rehabilitation and other health care services by patients with joint replacement after discharge from skilled nursing and inpatient rehabilitation facilities. Arch Phys Med Rehabil. 2009; 90:1297-1305.
32. Moffet, H, Collet, JP, Shapiro, SH, et al. Effectiveness of intensive rehabilitation on functional ability and quality of life after first total knee arthroplasty: A single-blind randomized controlled trial. Arch Phys Med Rehabil. 2004; 85:546-556.
33. LaStayo, PC, Meier, W, Marcus, RL, et al. Reversing muscle and mobility deficits 1 to 4 years after TKA: a pilot study. Clin Orthop Relat Res. 2009; 467:1493-1500.
34. Valtonen, A, Poyhonen, T, Sipila, S, et al. Effects of aquatic resistance training on mobility limitation and lower-limb impairments after knee replacement. Arch Phys Med Rehabil. 2010; 91:833-839.

35. Piva, SR, Gil, AB, Almeida, GJ, et al. A balance exercise program appears to improve function for patients with total knee arthroplasty: a randomized clinical trial. Phys Ther. 2010; 90:880-894. doi:ptj.20090150 [pii];10.2522/ptj.20090150 [doi].
36. Piva, SR, Almeida, GJ, and Wasko, MC. Association of physical function and physical activity in women with rheumatoid arthritis. Arthritis Care Res (Hoboken). 2010; 62:1144-1151.
37. Almeida, GJ, Wasko, MC, Jeong, K, et al. Physical activity measured by the SenseWear Armband in women with rheumatoid arthritis. Phys Ther. 2011; 91:1367-1376.
38. Parmelee PA, Thuras PD, et al. Validation of the Cumulative Illness Rating Scale in Geriatric Residential Population. JAGS. 1995; 43:130-137.
39. Fortin M, Steenbakkers K, et al. The electronic Cumulative Illness Rating Scale: a reliable and valid tool to assess multi-morbidity in primary care. Journal of Evaluation in Clinical Practice. 2011; 17:1089-1093.
40. Miller M, Towers A. A Manual of Guidelines for Scoring the Cumulative Illness Rating Scale for Geriatrics (CIRS-G). University of Pittsburgh, School of Medicine, Department of Geriatric Psychiatry, Western Psychiatric Institute and Clinic. 1991.
41. Linn, BS, Linn, MW, and Gurel, L. Cumulative illness rating scale. J Am Geriatr Soc. 1968; 16:622-626.
42. Bellamy, N. WOMAC Osteoarthritis Index User Guide. Version V. Brisbane, Australia 2002.
43. Bellamy, N, Buchanan, WW, Goldsmith, CH, et al. Validation study of WOMAC: a health status instrument for measuring clinically important patient relevant outcomes to antirheumatic drug therapy in patients with osteoarthritis of the hip or knee. J Rheumatol. 1988; 15:1833-1840.
44. Bellamy, N, Kean, WF, Buchanan, WW, et al. Double blind randomized controlled trial of sodium meclofenamate (Meclomen) and diclofenac sodium (Voltaren): post validation reapplication of the WOMAC Osteoarthritis Index. J Rheumatol. 1992; 19:153-159.
45. Hawker, G, Melfi, C, Paul, J, et al. Comparison of a generic (SF-36) and a disease specific (WOMAC) (Western Ontario and McMaster Universities Osteoarthritis Index) instrument in the measurement of outcomes after knee replacement surgery. J Rheumatol. 1995; 22:1193-1196.
46. Stewart, AL, Verboncoeur, CJ, McLellan, BY, et al. Physical activity outcomes of CHAMPS II: a physical activity promotion program for older adults. J Gerontol A Biol Sci Med Sci. 2001; 56:M465-M470.
47. Lopez-Olivo, MA, Landon, GC, et al. Psychosocial determinants of outcomes in knee replacement. Ann Rheum Dis. 2011; 70:1775-1781.
48. Lorig, K, Chastain, RL, Ung, E, et al. Development and evaluation of a scale to measure perceived self-efficacy in people with arthritis. Arthritis Rheum. 1989; 32:37-44.
49. Hornyak, V, Brach, JS, et al. What is the relation between fear of falling and physical activity in older adults? ACRM. 2013; 94:2529-34.
50. Andresen, EM, Malmgren, JA, Carter, WB, et al. Screening for depression in well older adults: evaluation of a short form of the CES-D (Center for Epidemiologic Studies Depression Scale). Am J Prev Med. 1994; 10:77-84.
51. Scopaz, KA, Piva, SR, Wisniewski, S, et al. Relationships of fear, anxiety, and depression with physical function in patients with knee osteoarthritis. Arch Phys Med Rehabil. 2009; 90:1866-1873.
52. Beck AT, Epstein N, Brown G, et al. An inventory for measuring clinical anxiety: psychometric properties. J Consult Clin Psych. 1988; 56:893-897.
53. Riddle, DL, Wade, JB, Jiranek, WA, et al. Preoperative pain catastrophizing predicts pain outcome after knee arthroplasty. Clin Orthop Relat Res. 2010; 468:798-806.

54. Riddle, DL, Wade, JB, and Jiranek, WA. Major depression, generalized anxiety disorder, and panic disorder in patients scheduled for knee arthroplasty. J Arthroplasty. 2010; 25:581-588.
55. Woby, SR, Watson, PJ, Roach, NK, et al. Adjustment to chronic low back pain--the relative influence of fear-avoidance beliefs, catastrophizing, and appraisals of control. Behav Res Ther. 2004; 42:761-774.
56. Fitzgerald, GK, White, DK, and Piva, SR. Associations for change in physical and psychological factors and treatment response following exercise in knee osteoarthritis: an exploratory study. Arthritis Care Res (Hoboken). 2012; 64:1673-1680.
57. Swartzman, LC, Gwadry, FG, Shapiro, AP, et al. The factor structure of the Coping Strategies Questionnaire. Pain. 1994; 57:311-316.
58. Geisser, ME, Robinson, ME, and Henson, CD. The Coping Strategies Questionnaire and chronic pain adjustment: a conceptual and empirical reanalysis. Clin J Pain. 1994; 10:98-106. Reenen, Mv, Oppe. EQ-5D-3L User Guide Basic information on how to use the EQ-5D-3L instrument. Version 5.1. 2015.
59. Zhao W, Hill MD, Palesch Y. Minimal sufficient balance--a new strategy to balance baseline covariates and preserve randomness of treatment allocation. StatMethods MedRes. 2012. doi:0962280212436447 [pii];10.1177/0962280212436447 [doi].
60. Dobson, F, Bennell, KL, et al. Recommended performance-based tests to assess physical function in people diagnosed with hip or knee osteoarthritis. Osteoarthritis Research Society International (OARSI). University of Melbourne, Australia. 2013.
61. Piva, SR, Fitzgerald, GK, Irrgang, JJ, et al. Get up and go test in patients with knee osteoarthritis. Arch Phys Med Rehabil. 2004; 85:284-289.
62. Almeida, GJ, Schroeder, CA, Gil, AB, et al. Interrater reliability and validity of the stair ascend/descend test in subjects with total knee arthroplasty. Arch Phys Med Rehabil. 2010; 91:932-938.
63. de Brito, LB, Ricardo, DR, de Araujo, DS, et al. Ability to sit and rise from the floor as a predictor of all-cause mortality. Eur J Prev Cardiol. 2012.
64. Piva, SR, Teixeira, PE, Almeida, GJ, et al. Contribution of hip abductor strength to physical function in patients with total knee arthroplasty. Phys Ther. 2011; 91:225-233.
65. Mizner, RL, Petterson, SC, Stevens, JE, et al. Preoperative quadriceps strength predicts functional ability one year after total knee arthroplasty. J Rheumatol. 2005; 32:1533-1539.
66. Hochberg, Y. A sharper Bonferroni procedure for multiple tests of signficance. Biometrika 75, 800-802. 1988.o P, Roth PL, and Buster MA. The Usefulness of Unit Weights in Creating Composite Scores: A Literature Review, Application to Content Validity, and Meta-Analysis. Organizational Research Methods. 2007; 10:689-709.
67. Zhao W. Mass weighted urn design - A new randomization algorithm for unequal allocations. Contemp Clin Trials. 2015;43:209-16. doi:10.1016/j.cct.2015.06.008.
68. Nagelkerke, N, Fidler, V, Bernsen, R, et al. Estimating treatment effects in randomized clinical trials in the presence of non-compliance. Stat Med. 2000; 19:1849-1864.
69. Lombard, DN, Lombard, TN, and Winett, RA. Walking to meet health guidelines: the effect of prompting frequency and prompt structure. Health Psychol. 1995; 14:164-170.
70. Martens, EP, Pestman, WR, de, BA, et al. Instrumental variables: application and limitations. Epidemiology. 2006; 17:260-267.
71. Maracy, M and Dunn, G. Estimating dose-response effects in psychological treatment trials: the role of instrumental variables. Stat Methods Med Res. 2011; 20:191-215.

72. Kennedy, DM, Stratford, PW, Wessel, J, et al. Assessing stability and change of four performance measures: a longitudinal study evaluating outcome following total hip and knee arthroplasty. BMC Musculoskelet Disord. 2005; 6:3.
73. Rejeski, WJ, Ettinger, WH, Jr., Schumaker, S, et al. Assessing performance-related disability in patients with knee osteoarthritis. Osteoarthritis Cartilage. 1995; 3:157-167.
74. Friedman LM, Furberg CD, and DeMets DL. Fundamentals of Clinical Trials. 4th ed. New York, NY: Springer Science, 2010.

APPENDIX *(documents from appendix are available upon request)*

A. **Study Forms**
B. **Study Forms Coded**
C. **Scoring**
D. **Informed Consent**
E. **Study Advertisement**

Manual of Operational Procedures (MOP)

for the research study:

"A comparison of treatment methods for patients following total knee replacement"

Principal Investigator:

Sara R. Piva, PhD, PT

Supported by the Patient Centered Outcomes Research Institute (PCORI)

CER-1310-06994

Version 1.1

2016

CONTENTS

1. STUDY OBJECTIVES 5
1.1. Primary Aim 5
1.2. Secondary Aim 5
1.3. Exploratory Aim 5
2. BACKGROUND 5
2.1. Rationale .... Error! Bookmark not defined.
2.2. Supporting Data .... Error! Bookmark not defined.
3. STUDY DESIGN 7
3.1. Overview of Study Design 7
3.2. Summary of Study Procedures 10
3.3. Summary of Study Recruitment and Screening Procedures 11
4. SELECTION AND ENROLLMENT OF SUBJECTS 12
4.1. Inclusion/Exclusion Criteria 12
4.2. Telephone Screening Process and In-person Eligibility Screening Visit Scheduling 13
4.3. In-person Eligibility Screening 14
5. BASELINE TESTING 15
5.1. Self-Reported Measures: 15
5.2. Clinical Examination 16
6. RANDOMIZATION 22
7. MASKING 22
8. STUDY INTERVENTIONS 22
8.1. Clinic-based Individual Outpatient Rehabilitative Exercises 22
8.2. Community-based Group Exercise 23
8.3. Usual Medical Care (waited-list control group) 23
9. EXERCISE COMPLIANCE 24
10. FOLLOW-UP PHONE-CALLS 24
11. FOLLOW-UP IN-PERSON TESTING 24
11.1. Three and Six Months Testing Sessions (and Nine Months for the Usual Medical Care Group) 25

11.2. Wait-list Usual Care group at the 6 months visit 26
12. CLINICAL MEASURES 26
12.1. Outcome Measures 26
12.1.1. Primary Outcome Measure 26
12.1.2. Secondary Outcome Measure 26
12.1.3. Other Measures 28
13. ADVERSE EVENTS 29
13.1. Management 29
13.2. Report 29
14. STUDY COMPENSATION 30
15. DATA ANALYSIS AND SAMPLE SIZE JUSTIFICATION 30
15.1. Primary Hypothesis 30
15.2. Secondary Hypothesis 31
15.3. Exploratory Aim 31
16. DROPOUT AND MISSING DATA 32
17. RECORD KEEPING 32
18. DATA MANAGEMENT PLAN 34
19. QUALITY ASSURANCE 34
20. DATA SAFETY AND MONITORING BOARD 35
21. HUMAN SUBJECTS 36
21.1. Institutional Review Board (IRB) Review and Inform Consent 36
21.2. Risks/Benefits Assessment 36
21.3. Potential Risks 37
21.4. Risk Management and Emergency Response 37
21.5. Confidentiality 37
21.6. Potential Benefits of the Proposed Research to Human Subjects and Others 38
21.7. Importance of the Knowledge to be Gained 38
22. STUDY ORGANIZATION AND ADMINISTRATION 38
22.1. Administration/ Research Personnel 38
23. STUDY PLACES 39
23.1. Community Centers 39
23.2. PT-CTRC 39

24. RESEARCH ENGAGEMENT PLAN 39
25. STUDY OPERATIONS 41
26. PUBLICATIONS 41
27. AMENDMENTS 41
28. REFERENCES 42
APPENDIX *(documents from appendix are available upon request)* 47
A. Study Forms 47
B. Study Forms Coded 47
C. Scoring 47
D. Informed Consent 47
E. Study Advertisement 47

Table of Figures

Figure 1- Study Design 8
Figure 2 - Study Flow Chart 9

Table of Tables

Table 1. Clinical measures completed at each time point. 27
Table 2. Type of data storage 33

1. STUDY OBJECTIVES

This study is a pragmatic comparative effectiveness study of exercise for patients following total knee replacement (TKR), designed as a 3-group randomized clinical trial. The main goal is to provide evidence to inform the choice of exercise programs during later stages after TKR.

1.1. Primary Aim

The primary aim of the study is to compare the outcomes of physical function and physical activity between the three groups: 1) clinic-based individual outpatient rehabilitative exercise; 2) community-based group exercise and 3) usual medical care waited-list.

The first hypothesis is that subjects in groups 1 and 2 will demonstrate greater improvement in physical function as compared to group 3. Physical function will be assessed using a self-reported questionnaire the Western Ontario and McMaster Universities Arthritis Index-WOMAC, and assessed by a battery of performance-based tests germane to patients post-TKR (walking speed, chair rise, stair climbing, single leg stance, 6-minute walk, and rising from the floor). The second hypothesis is that subjects in groups 1 and 2 will demonstrate larger increases in physical activity as compared to group 3. Physical activity will be measured in real-time using a portable monitor (SenseWear Armband) and by a questionnaire (Community Healthy Activities Model Program for Seniors-CHAMPS).

1.2. Secondary Aim

The secondary aim of this study is to identify baseline predictors of functional recovery for both exercise groups. The hypothesis with this aim is a group of baseline biomedical (age, sex, education, chronicity of disease, physical function), physical impairments (range of motion, pain, muscle strength), and psychosocial measures (fear of activity, coping, depression, self-efficacy, expectation) will be associated with treatment response. The expectation is that the predictors of treatment response to be different in the treatment groups.

1.3. Exploratory Aim

This study also has an exploratory aim to determine attrition, adherence, adverse events and co-interventions across treatment groups. The hypothesis related to this aim is adherence and co-interventions will be similar in all groups. The attrition rate and adverse events- mainly number of falls- will be lower in groups 1 and 2 compared to group 3.

This study will inform the choice of interventions for later stages after TKR and will provide evidence for the design of public health programs to extend the number of years free of disability in this population and to tailor interventions according to patient characteristics.

2. BACKGROUND

The demand for TKR is growing exponentially. Over 4 million US adults currently live with a TKR and it is projected that greater than 3 million TKRs will be performed annually in the US by 2030.[1] The lifetime risk of undergoing TKR is 8% for all persons.[2] TKR represents the highest aggregate cost among the fast increasing

surgical procedures, posing a large economic burden on the US health system.[3] Patients who undergo TKR experience considerable functional limitations, muscle weakness, de-conditioning, physical activity,[4-7] and also represent a rapidly increasing population with multiple comorbidities. Around 49% are overweight or obese, 16% have diabetes and 50% have high blood pressure.[8,9]

By most metrics, TKRs are successful surgeries, as they reduce pain and are cost-effective. [10,11] However, long-term functional and activity limitations, due to chronic joint disease prior to surgery, do not spontaneously resolve after TKR. Limitations in basic activities such as walking and managing stairs along with knee pain remain for years after TKR.[12-18] A study found that after one year, 52% of subjects who have received TKR surgeries continued to have substantial limitations during biomechanically demanding activities such as kneeling, squatting, turning and cutting, carrying loads, playing tennis, dancing, gardening, and sexual activity, in contrast to only 22% of matched controls.[19] Subjects post-TKR are also at increased risk for falls, [20] do not reach recommended levels of physical activity to prevent morbidity, [21] and gain weight in the years after surgery. [22] Patients have voiced that aside from the stress and disruption in their lives caused by the TKR, they are not getting the later benefit they expected.[23]

Persistent functional limitations combined with physical inactivity post-TKR are a major public health concern as they are precursors to disability and comorbidities. For example, decreased walking speed is a risk factor for falls, future disability, and mortality,[24-27] physical inactivity is a risk factor for hypertension, diabetes, obesity, cardiovascular disease, and cognitive decline.[28] These limitations can also affect the ability of older adults to stay in the workforce and live independently. Hence, there is an urgent need to overcome the persistent disability and physical inactivity of these patients to averting permanent disability and comorbid conditions.

Rehabilitation is a simple solution to alleviate the functional limitations, promote physical activity, and enhance TKR outcomes. While virtually all patients receive rehabilitation for about 1-2 months (early stage) after surgery,[29-31] studies demonstrated very modest benefits of rehabilitation during the first months post-TKR.[28] These modest benefits are not surprising; during the initial months post-TKR, patients are still healing from the major surgical wounds, and rehabilitative exercises are only able to focus on improving knee movement and promoting safe and independent mobility. It is not realistic to expect that early post-op rehabilitation alone can reverse years (or decades) of pre-operative muscle weakness, deconditioning and functional limitations. Participation in more extended exercise programs that intensively target the muscle weakness, deconditioning, and poor mobility is likely the only way to reverse these persistent deficits. Exercise intervention should be continued at a later stage post-TKR when patients can tolerate intense doses of exercise required to promote substantial changes.

Despite the misconception that patients reach a limit in their recovery within a few months post-TKR, emerging evidence indicates that patients who perform intense exercise at later stages post-TKR (at least 2 months post-op) achieve substantial functional recovery.[32-35] While the collective findings from these studies emphasize that patients can tolerate intense exercise programs at later stages post-TKR and that it has potential to recover function, the evidence from these studies is limited due to small samples or non-rigorous research methods. [32-35]

The effect of exercise used at later stage post-TKR has been an understudied area of research with a large gap in knowledge that affects patients, clinicians and extends to healthcare payers and policymakers. They lack the

compelling evidence to alter healthcare policy decisions about the management of patients at later stage post-TKR, to enhance the outcome of this prevalent and expensive surgery. Due to currently limited evidence, only a small fraction of patients are referred for rehabilitative exercises at later stages (2 months or more) post-TKR. This results in the majority of patients receiving insufficient care. They are prematurely discharged from rehabilitation before exercises can be intensified to enhance surgical outcome. Healthcare providers state they simply lack good evidence for such recommendations, [23] leaving patients and providers without guidance to inform decisions on prevention of morbidity and maximize the benefits of TKR.

3. STUDY DESIGN

3.1. Overview of Study Design

This comparative effectiveness study is designed to combine patient-centered research questions with rigorous research methods that minimize bias and balance internal and external validity. The study is designed as a three-group single-blind randomized clinical trial. Eligible subjects undergo baseline assessment and are randomized in a 2:2:1 allocation to one of the 3 groups: 1) clinic-based individual outpatient rehabilitative exercise; 2) community-based group exercise classes; or 3) usual medical care. The usual medical care group continues their usual care whereas the other two groups receive an exercise intervention for 12 weeks. Endpoint measures are assessed in-person at 3 and 6 months after randomization. Participants are also interviewed over the phone at 1.5 and 4.5 months after randomization to promote retention. After the 6 month follow-up, subjects in the usual medical care group are randomized to either clinic-based individual outpatient rehabilitation exercise or community-based group exercise and participate in a phone interview at 7.5 months and in-person assessment at 9 months after initial randomization. Figure 1 gives an overview of study design.

The design of this study is unbalanced with an unequal number of subjects per group, meaning that the exercise groups have twice the number of subjects as the control group. The unbalanced design was chosen because larger functional recovery is expected in both exercise groups as compared to the usual care group, thus requiring larger sample size in the two exercise arms to detect smaller differences between them as compared to larger differences expected between either of the exercise arms and the usual care arm.

Figure 1- Study Design

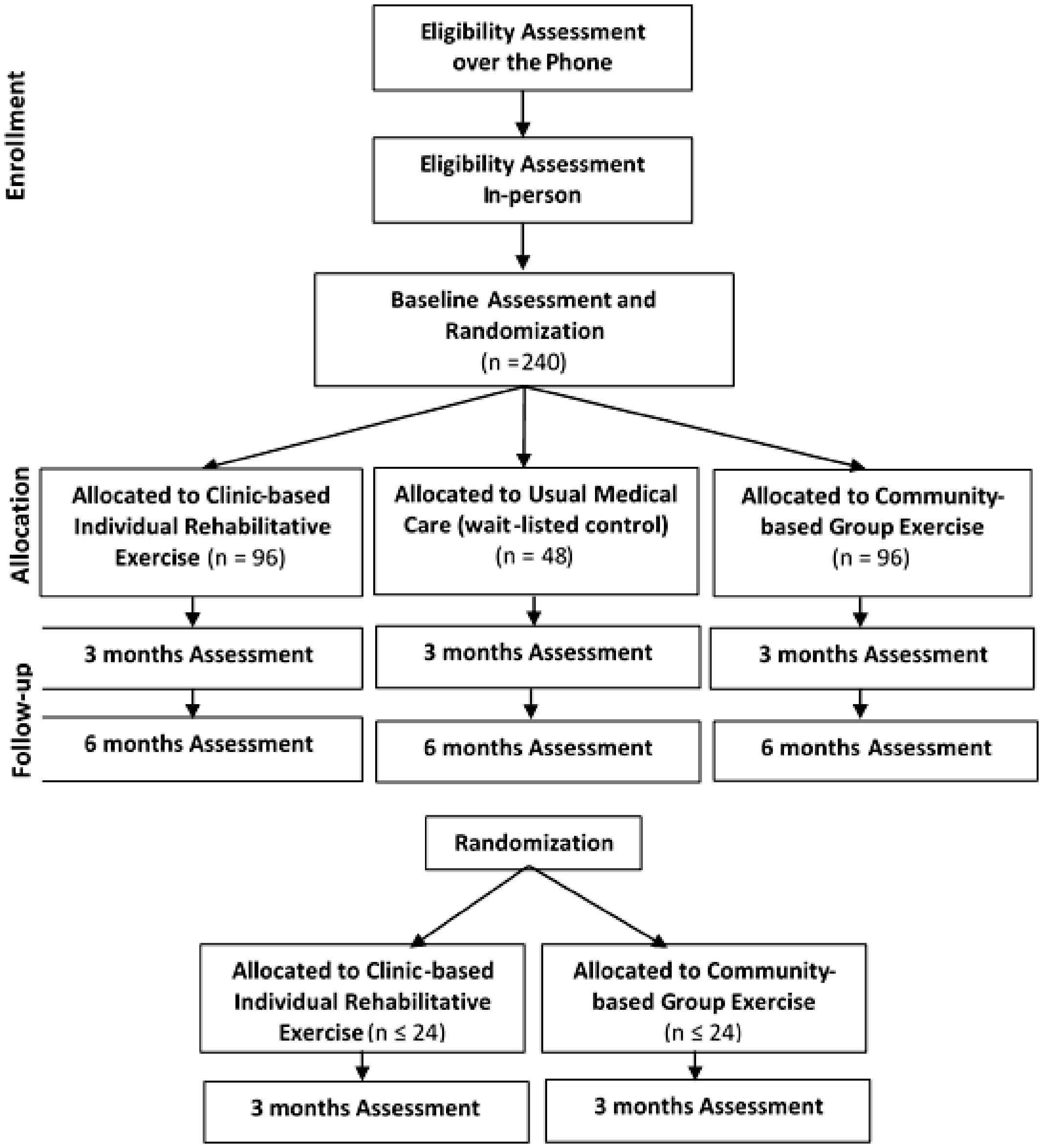

Figure 2 - Study Flow Chart

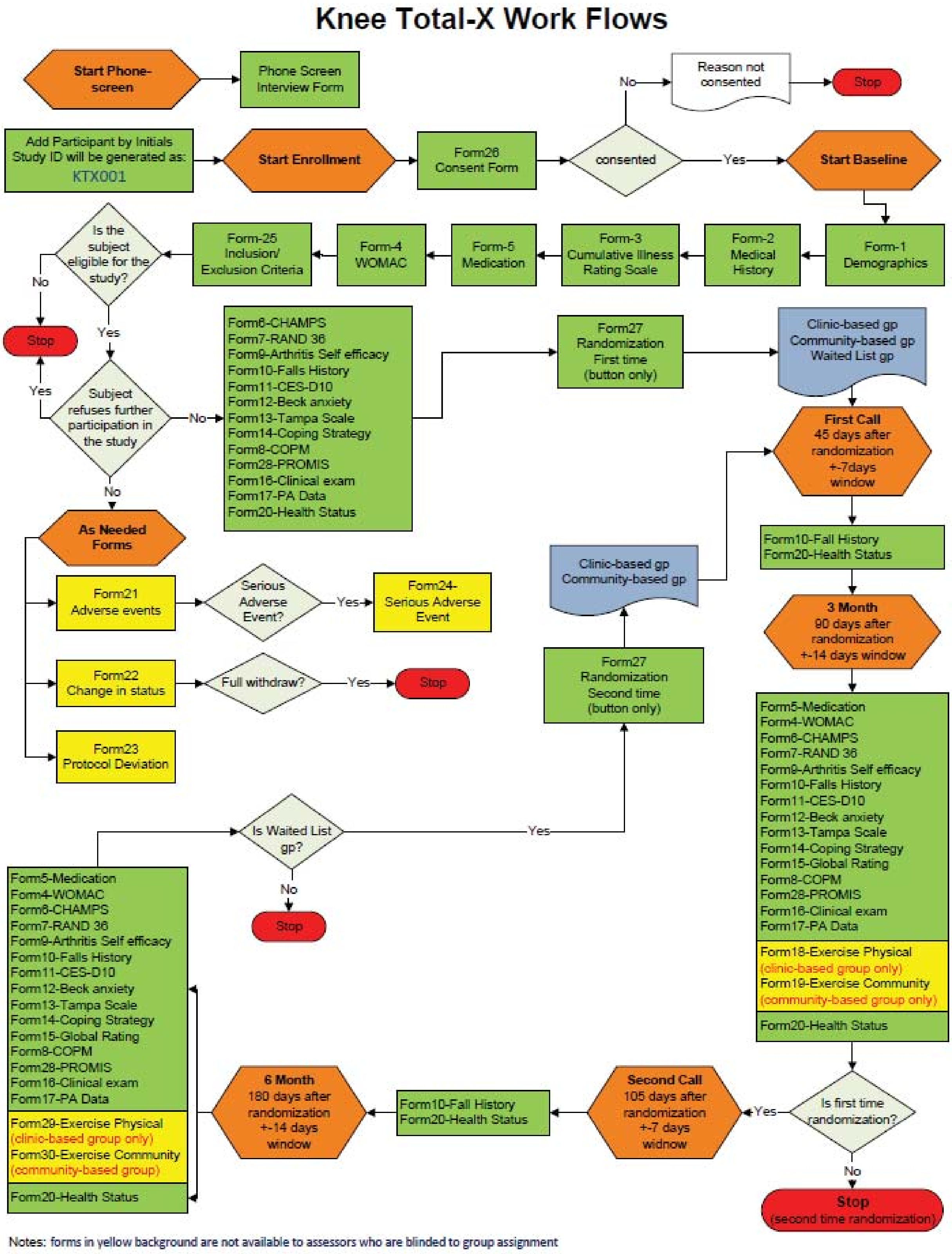

3.2. Summary of Study Procedures

For organization purposes, this study is divided into 6 main elements of study procedures. Figure 2 describes the flow chart of these procedures along with paths to continue or not study procedures, and the main elements are shown in the orange hexagons.

3.2.1. Telephone Screening: potential subjects that called the research team are contacted for telephone screening by the study research coordinator/trained research assistants. During this phone call, information about the study is given to subjects and they are screened for eligibility to participate in the study.

3.2.2. Medical Release process: subjects deemed eligible on the phone screening need clearance from the surgeon to schedule the in-person screening visit. A medical release is obtained by the research assistants and the coordinator of the study, through fax or email. Study research assistants send the Medical Referral form to the surgeons (or their assistants), and other physicians (e.g. cardiologist, primary care physician, etc) in case there is any concern with other health conditions.

3.2.3. Schedule in-person eligibility visit: after the medical release is obtained, the research coordinator can schedule in-person eligibility visit. The tentative appointment is scheduled with the subject, and it is confirmed after tester confirms availability. Once this is done, a package for in-person eligibility visit is prepared and mailed to the subject, containing: a letter with general instructions for the appointment, informed consent and driving directions to the research facility with parking instructions.

3.2.4. Call to confirm appointment: research coordinator calls subjects scheduled for the next day to confirm/reschedule the appointment. During this phone call, the coordinator must ask if the subject received, and if he/she had any questions on, the material sent in the eligibility package.

3.2.5. In-person screening visit: Trial coordinator goes over the informed consent document with the subject to clarify any questions. If the subject agrees with all study procedures, both the subject and the research coordinator sign 2 copies of the informed consent document (one copy stays with the coordinator and the other one is given to subject). The research coordinator turns study laptop on, connects the laptop to the Wi-Fi (if not done automatically) and open KTX database (http://www.crhc.pitt.edu/KneeTotalX/Default.aspx). Once in the KTX database webpage, the research coordinator logs in and add the new subject into the system (subject is given a study identification number). The subject is asked to complete questionnaires (Demographics, Medical History form and the Western Ontario and McMaster Universities Osteoarthritis Index Physical Function Subscale - WOMAC-PF) in the database, and the research coordinator checks the list of current medications (provided by the subject), and interviews the subject to collect comorbidity data using the Cumulative Illness Rating Scale. The Inclusion/Exclusion form is a smart form (using data collected in the screening visit) to deem subject's final eligibility to participate in the study.

3.2.6. Baseline visit: usually occurs on the same day immediately after the in-person eligibility screening visit. A series of self-reported questionnaires are completed by the eligible subject followed by a clinical evaluation.

3.2.7. Intervention: delivered for 3 months. Intervention sessions can be extended if there is a medical condition that requires immediate treatment and interruption of the intervention. Intervention will resume after release from the treating physician and/or subside once symptoms are resolved. Intervention will be discontinued if the subject was harmed during the sessions or deemed unsafe.

3.2.8. Phone follow-up: occurs every 1.5 months subjects are in the study. The trial coordinator calls subjects at every 1.5 months once they are in the study to track adverse events, exercise compliance and co-interventions. The window for the phone calls to be done is 1 week prior and after to phone call target date (totalizing 2 weeks).

3.2.9. In-person follow-up testing sessions: scheduled after 3 months, 6 months and 9 months from the randomization date. The window for the testing sessions to be scheduled is 1 week prior to the target date, and 3 weeks after the target date (totalizing 1 month).

3.3. Summary of Study Recruitment and Screening Procedures

The study uses several recruitment strategies with the intent of enrolling 8 to 10 subjects each month. Study recruitment started in December 2014 and it will be continued until December 2016. The primary strategy comprises of invitations sent to recent TKR patients directly from the knee surgeons who performed the procedure. Knee surgeons recruit participants either during a regular follow-up visit or send letters to their patients offering study participation. We anticipate participation in this recruitment strategy from 10 to 15 knee surgeons from several clinics, and who operate in different hospitals within Allegheny County.

Additional recruitment strategies include direct mailings of postcards to local neighborhoods, study letters sent to participants of research registries (Clinical and Translational Science Institute -CTSI - Registry and the Pittsburgh Claude D. Pepper Older Americans Independence Center Registry), advertisements on local radio stations, newspapers, and other publications. The study also recruits subjects directly from the Vintage Community Senior Center and the Squirrel Hill Jewish Community Center. Both centers are designated community senior citizen centers by the Allegheny County Area Agency on Aging. The directors of these centers have agreed to assist with the study's recruitment efforts by allowing posters and informational brochures to be placed in their facilities, by sending e-blasts to their respective members, and by posting announcements about the research study in their newsletters.

For all recruitment efforts, we use services from the University of Pittsburgh - University Marketing Communications (UMC). The UMC is a resource available to researchers at the University of Pittsburgh, which has a full-time staff that provides advertising, planning, copy-writing, design, and production services and handles reservation of newspaper space.

All recruitment materials have the research coordinator's telephone number so that subjects may contact the research team, if interested in the study. Thus, regardless of the recruitment method, the potential subjects initiate contact with study personnel. All individuals who call to inquire about the study give their verbal consent to undergo telephone screening. Those deemed potentially eligible over the phone are scheduled for an in-person assessment to sign informed consent and re-confirm eligibility. If eligibility is confirmed during the in-person assessment, the participant undergoes an in-person screening assessment (Figure 1).

4. SELECTION AND ENROLLMENT OF SUBJECTS

4.1. Inclusion/Exclusion Criteria

The study enrolls adults older than 60 years of age from Allegheny County who underwent a unilateral TKR 2 to 4 months prior to study participation. Thus, subjects have healed from the surgical insult and knee pain, effusion and motion are improved, and are no longer restricted from more intense exercises. Participants also have to experience at least moderate functional limitation in daily activities to represent those with persistent limitations after TKR (minimum score of 9 points on the Western Ontario and McMaster Universities Osteoarthritis Index Physical Function Subscale -WOMAC-PF), speak English sufficiently to understand study instructions, be willing to be randomized to one of the three treatment groups, and have a medical clearance to participate in the study. Subjects will be excluded from the study if they meet any the following criteria:

- Absolute or relative contraindication to exercise testing by the American College of Sports Medicine/American Heart Association
  - Absolute Contraindications to Exercise:
    - A recent significant change in the resting ECG suggesting significant ischemia, recent myocardial infarction (within 2 months) or other acute cardiac event;
    - Unstable angina
    - Uncontrolled cardiac dysrhythmias causing symptoms or hemodynamic compromise
    - Symptomatic severe aortic stenosis
    - Uncontrolled symptomatic heart failure
    - Acute pulmonary embolus or pulmonary infarction
    - Acute myocarditis or pericarditis
    - Suspected or known dissecting aneurysm
  - Relative Contraindications to Exercise:
    - Left main coronary stenosis
    - Moderate stenotic heart disease
    - Electrolyte abnormalities (e.g. hypokalemia, hypomagnesemia)
    - Tachydysrhythmia or Bradydysrhythmia
    - Hypertrophic cardiomyopathy and other forms of outflow tract obstruction
    - High degree atrioventricular block
    - Ventricular aneurysm
- Uncontrolled cardiovascular disease or hypertension

- Current total knee replacement is a revision
- Unable to walk 50 meters without an assistive device and to comfortably bear weight on the surgical knee
- History of muscular or neurologic disorder that may affect lower extremity function (e.g. muscular dystrophy, Parkinson's disease, multiple sclerosis)
- Participates in structured exercise more than twice a week
- Has a terminal illness
- Plans to have another total joint replacement in the lower extremities during study period
- Plans to relocate outside the immediate area during study period
- Refuses to participate in study protocol

4.2. Telephone Screening Process and In-person Eligibility Screening Visit Scheduling

All subjects who call to inquire about the study are asked for verbal consent to undergo telephone screening. The telephone screening is performed by the research staff of the study and contains questions regarding subject's demographic information, medical history and functional questionnaire (WOMAC-PF) that comprises eligibility criteria.

Research staff follows the telephone screening form (see **APPENDIX: A. Study Forms**: Telephone Screening form).

When a subject is eligible to be in the study, the research staff informs the research coordinator, who then schedules an in-person visit to perform the final screen and determines if the subject meets eligibility criteria to participate in the study.

The tentative appointment is confirmed with the physical therapist who will also evaluate the subject during the baseline visit in case eligibility is confirmed. The research coordinator then prepares an in-person eligibility screening/baseline mail package to be sent to subjects in preparation for the visit. The mailed package contains a copy of the informed consent document, driving directions to get to the research facility and a letter with general guidelines for the entire visit.

Prior to the in-person eligibility screening visit taking place, the research coordinator requests and obtains a medical release form from the surgeon for the subject to participate in the study. Additional medical releases (from a cardiologist and/or primary care physician) may be required in cases when subject answers any 'Yes' to the following questions on the telephone screening:

1. Has your doctor ever said that you have a heart condition and that you should only do physical activity recommended by a doctor?
2. Do you feel unreasonably out of breath?
3. Do you experience dizziness, fainting, or blackouts?
4. Do you feel pain in your chest when you do physical activity?
5. In the past month, have you had chest pain when you were not doing physical activity?
6. Is your blood sugar often above what doctor recommended?

7. Do you feel shaky, confused, or dizzy when you exercise?

The appointment is confirmed with the subject one day prior to the scheduled visit. The research coordinator must contact the subject to verify if the subject has any questions about the study (after reading the informed consent), remind subject to bring an updated list of medications, discuss transportation and directions, and re-confirm or reschedule the appointment.

4.3. In-person Eligibility Screening

At the in-person eligibility screening visit, the research coordinator greets and guides the subject to a clinical examination room. There, the research coordinator explains the study to the potential subject and clarifies any questions the subject might have. If the subject has no questions or after addressing the subject's concerns, both the coordinator and the subject sign 2 copies of the informed consent (1 signed copy is given to the subject and the other one is kept in a locked research file).

All subjects are asked to complete a Contact Information Form (see **APPENDIX: A. Study Forms**: Contact Information form) that is used for compensation purposes (i.e., to assign a pre-paid card - WePay System- to the subject). This form is stored in a locked file, separate from research data, to prevent breaches of confidentiality. The research coordinator logs-in the study web system and enters the subject's initials. When subject's initials are submitted, the database generates the study subject ID. The convention chosen for the subjects' IDs is the acronym of the study KTX followed by 3 digits (e.g. KTX001, KTX002, etc.)

Prior to completing the inclusion/exclusion criteria smart form, the database requires confirmation that the subject signed the informed consent form followed by the respective date in which the consent was obtained. This is a required step in the electronic database to certify that the study is in compliance with the University of Pittsburgh Institutional Review Board. Once the information is recorded the database, this allows access to study data collection forms.

The following forms are completed in the order provided below to confirm subject's eligibility (**APPENDIX: A. Study Forms**):

1. Demographics Form: demographical information is recorded in this form.
2. Medical History Form: records subject's body mass index (BMI in kg/m^2), systolic and diastolic blood pressure, information about subject's TKR surgery (surgery date, TKR side, surgical technique), other total joint replacement in the lower extremities, medical conditions that affect subject's mobility, information on the standard rehabilitation the subject received after the TKR, if subject is engaged in supervised exercise and the type of exercise, and the subject's expectation on study's exercise programs.
3. Cumulative Illness Rating Scale: measures the severity of the subject's comorbidities.[38-41] The Cumulative Illness Rating Scale is ranked from 1 to 5 (1=None, 2=Mild, 3=Moderate, 4=Severe, 5=Extreme), according to the severity of a person's comorbidity within a system of the body. The description of each ranking option is on the form. All questions marked as "Severe" or "Extreme" in this form will exclude the person from being in the study, as the condition is considered disabling.
4. Medication Form: records the name and dosage of the medications taken by the subject to improve knee symptoms.

5. The Western Ontario and McMaster Universities Osteoarthritis Index (WOMAC): This form assesses the level of pain, stiffness and physical function while performing daily activities. This form was developed for people with hip and/or knee osteoarthritis. This is the primary outcome measure of the study.[42-45]
6. Inclusion/Exclusion form: inclusion and exclusion criteria are checked. Specific information that is collected in previous forms (e.g. unilateral TKR – "YES" or "NO" collected in the Medical History form) are auto-populated from the respective forms. Criteria that are not collected from previous forms are asked to subject and entered manually by the research coordinator. Submission of this form establishes the subject's eligibility.

At any point during the screening process if a subject is deemed ineligible the evaluation is terminated and the subject is compensated for his/her time (see Section **14. STUDY COMPENSATION**).

If the subject is deemed eligible for the study, the research coordinator introduces the subject to the tester who continues onto baseline evaluation component of this visit.

5. BASELINE TESTING

5.1. Self-Reported Measures:

The baseline visit usually occurs on the same day as the in-person eligibility, unless subject requests the continuation to occur in a different day or in case there is a health concern (e.g. blood pressure above 170 x 110 mmHg) that would require a medical release from the primary care physician or other specialist. In this case, the continuation of the baseline visit is then scheduled after proper medical release is obtained.

The study tester provides the instructions and necessary assistance to subjects to complete the self-reported forms. (**APPENDIX: A. Study Forms**)

- Community Healthy Activities Model Program for Seniors (CHAMPS): measure self-reported physical activity. The CHAMPS questionnaire is formatted so that specific activities are listed on the form. Subject reports the activities he/she did in a typical week during the last 4 weeks, the number of times in a week he/she did the activity and the total amount of hours usually spent in that activity.[46]
- RAND-36: measures self-reported quality of life.[45]
- Arthritis Self-Efficacy Scale (ASES): this form has 3 subscales that are scored separately: pain, function and other symptoms subscales.[47,48]
- Falls History form: measure the number of falls the subject had in the past year.[49]
- Center of Epidemiologic Studies Depression Scale (CES-D10): measure depression symptoms.[50,51]
- Beck Anxiety Inventory: measure anxiety symptoms.[51,52]
- Tampa Scale for Kinesiophobia: 17 items scale that measures fear related to exercise activity.[53-56]
- Coping Strategies Questionnaire: measure self-reported coping strategies related to pain.[57-58]
- EQ-5D: measure general health status.[59]
- Canadian Occupational Performance Measure (COPM): measure self-reported performance and satisfaction of daily tasks that are deemed important to the subject.

- Patient-Reported Outcomes Measurement Information System (PROMIS) Physical Function & Pain Interference: questionnaire administered via Computerized Adaptive Testing (CAT). It measures self-reported physical function and self-reported pain interference during daily and social activities.
-

5.2. Clinical Examination

The Clinical Examination procedures are conducted by the tester. The results of each test are recorded on a hard copy of the Clinical Examination form (**APPENDIX: A. Study Forms**: Clinical Examination form) and immediately transposed into the database. The database is set up with possible ranges for the results of each test to avoid typos while recording data. All tests are performed on the non-operative side first and operative side second.

- Clinical Examination procedures:
  1. Knee range of motion: All ROM measures are assessed with a standard goniometer with the patient in supine on a treatment table. For all tests, the non-tested lower extremity stays flat over the treatment table in neutral rotation.
     a. Passive knee flexion: Participant is in supine with the tested knee in extension. Initially hip is in zero degrees of extension, abduction, and adduction. Tester flexes knee and hip by sliding the heel of the tested knee on the bed. Tester stabilizes the femur and bends the knee until reaching a soft tissue end feel or up to patient pain tolerance, whichever comes first. The fixed arm of the goniometer is placed over the lateral midline of the femur, referencing greater trochanter. The moving arm of the goniometer is over the lateral midline of the fibula, referencing lateral malleolus & fibular head. Possible ranges: 50 to 160 degrees.
     b. Passive knee extension: A bolster (i.e., folded towel or pillow) is placed under the participant's ankles so that knee hyperextension can be measured. The tester brings the knee close to full extension and then offers slight posterior pressure over the distal thigh until reaching a capsular end feel or pain tolerance (whichever comes first). The center of the goniometer is over the lateral epicondyle of the femur. The fixed arm of the goniometer is over the lateral midline of the femur, referencing greater trochanter. The moving arm of the goniometer is over the lateral midline of the fibula, referencing lateral malleolus & fibular head. In case the participant has a flexion contracture, the value is reported as negative (-). In the case of knee hyperextension, the value is positive (+) and recorded without a sign. Possible ranges: -30 to 20 degrees.

  2. Knee extension lag: measure through the performance of the straight leg raise test, and it assesses the ability to actively lift the lower extremity (LE) off the treatment table. The difference between the value of passive knee extension and knee extension during the active straight leg raise test. If the difference is ≥ 5° it represents a knee extension lag and is recorded as "Yes". If the difference is less than 5° it is recorded as "No".

  3. Single-leg balance test (SLS): The SLS is recommended in a battery of tests to quickly assess global functional level and its scores are related to risk for falls.

- Participants are asked to stand on one foot for 60 seconds. The other foot is raised so that the raised foot is near but not touching the ankle of their stance limb. The participant may use the arms, bend the knee, or move the body to maintain balance. The tester uses a stopwatch to measure the amount of time the participant is able to stand on one limb. Time commences when the participant raises the foot off the floor. Time ends when the participant either: (1) uses the raised foot (moved it toward or away from the standing limb or touched the floor or the other limb), (2) moves the weight-bearing foot to maintain his balance (ie, rotated foot on the ground), (3) a maximum of 60 seconds elapses. Three trials are performed on each side and recorded.
- **Tester Script:**
  Now I will show you the test. (Demonstrate) I want you to try to stand on one foot with the other foot raised near, but not touching the ankle, for about 60 seconds. You may use your arms, bend your knees, or move your body to maintain your balance, but try not to move your feet. Try to hold this position until I tell you to stop. We will do it three times.

Stand next to the participant to help him/her into the tandem position. Supply just enough support to the participant's arm to prevent loss of balance. When the participant has raised his/her foot, ask "Are you ready?" Then let go and begin timing as you say, "Ready, begin." Stop the stopwatch and say "Stop" after 60 sec. or when the participant steps out of position or grabs your arm or steady surface.[59]

4. Repeated Chair Stand Test: This is a test of sit-to-stand activity and of lower body strength and dynamic balance. Participants are asked to sit down on the chair and stand up to a full upright position, without help, repeatedly for 30 seconds.
    - Equipment: 2 timers/stopwatches, a straight back chair with a 44 cm (17 inch) seat height, preferably without arms (same chair should be used for re-testing).
    - Procedure: Participant sits in the chair in a position that allows them to place their feet flat on the floor, shoulder width apart, with knees flexed slightly more than 90 degrees so that their heels are somewhat closer to the chair than the back of their knees. The tester stands close to the side of the chair for safety and so as they can observe the technique, to ensure the participant comes to a full stand and full sit position during the test. A practice trial is recommended before testing to check technique and understanding.
    - Scoring: time (in seconds) participant takes to complete 5 sit-to-stands is recorded, and the tester counts the total number of chair stands the participant accomplished during 30 seconds. The participant can stop and rest if they become tired, but time keeps going.
    - **Tester Script:**
      "For this test, do the best you can by going as fast as you can but do not push yourself to a point of overexertion or beyond what you think is safe for you. Place your hands on the opposite shoulder so that your arms are crossed at the wrists and held close across your chest. Keep your arms in this position for the test. Keep your feet flat on the floor and at shoulder apart.On the signal of begin, stand up to a full stand position and then sit back down again so as your bottom fully touches the seat.Keep going until I say stop.Get ready and BEGIN."

5. Walking Ability Tests: walking aids are allowed to be used, but not recommended (unless there is a safety concern).

   a. Timed up and go test: This is a "transition" test of ambulatory activity that incorporates multiple activity themes (sit-to-stand activity, walking short distances, changing direction during walking, and the transitions between the activities). It is also a test of strength, agility and dynamic balance. The test records the time in seconds taken to rise from a chair, walk 3 meters (9ft 10inches), turn, walk back to the chair, then sit down. The use of walking aid is allowed but the participant may not be assisted by another person.

      - Equipment: timer/stopwatch, standard chair with armrests, tape, or other marker on the floor 3 meters (9ft 10inches), away from the chair so that it is easily seen by the participant and with enough room to turn safely. Ensure the chair cannot slide backward by placing the back of the chair against the wall. The participant sits in the chair with their back resting on the back of the chair and hands on the armrests. A chair of same height is needed for re-testing.
      - Tester: if slight safety concern, the tester stands to the side of the chair, then follows the participant to guard slightly behind and to one side but not as to pace or impede turn. If there is no concern for safety, the tester remains at the start/finish position beside the chair. A practice trial is recommended before testing to check understanding.
      - **Tester Script:**
        For this test, do the best you can and walk at your regular pace. Start by sitting in the chair with your back resting on the backrest and your hands on the armrests. On start, stand up, walk to the mark, turn around and sit back into the chair with your back resting on the back of the chair. Walk at your regular pace. Get ready and BEGIN.
        Timing starts on the signal to begin and terminates once the participant sits back down fully with their back resting on the back of the chair. One trial is performed and is recorded..[60,61]

   b. Time to walk 4-meters: This self-selected gait speed test assesses the participant's ability to walk 4 meters or 13 feet. For this test, the tester will need a stopwatch, measuring tape, and masking tape. The walking course should be set up prior to the assessment visits and the area should be free from clutter, unobstructed and should include at least an extra meter on each end. The tester will mark the start and finish lines on the floor using the masking tape and a construction meter tape to measure the correct distance.

      - The tester will ask the participant whether they feel safe walking a short distance with or without walking device for the test. If they don't, do not perform the gait speed test. A walking device can be used during the walk.
      - Participants are instructed to walk at their usual or normal walking speed (i.e., as they would normally walk to run errands) and past the finish line ~1 meter after the finish line. The tester will begin timing when the participant begins to move (not when they say "Ready, begin"). The tester will stop timing when the first foot crosses the masking tape finish line. The tester will record the time when the participant's first foot crosses the 4-meter line. It is imperative that the participant's foot crosses the line and not lands on the line as it does not end the test.
      - The tester will write the time on their data sheet. If unable to complete the test mark as

zero (not completed).

- The tester will not walk beside the participant during the gait speed test, as this may set a pace for the participant, but rather slightly behind and to the side and outside of the participant's visual field. For those that normally use a walking device, it is recommended that close attention is paid to these individuals during the test to prevent falling.
- If the tester has issues with the stopwatch, repeat the test.[60].

c. 40-meters Fast-Paced Walk Test: This test is a direct measure participant's ability to walk fast over short distances and changing direction during walking.

- Equipment: timer/stopwatch, 2 cones, bright colored tape. Mark out a 10m walkway with the bright colored tape on the floor. Place one cone 1m from each end of the marks.
- Procedure: The participant is asked to walk as fast as they can, as safely as possible, without running, along the 10m, then turn around a cone, return and repeat again for a total of 40m distance. The tester walks on the side of the participant, at the participant's pace.
- **Tester script:**
  "For this test, do the best you can by going as fast as you can, without running, but do not push yourself to a point of overexertion or beyond what you think is safe for you. Start with both feet on the start line. On start, walk as quickly but as safely as possible, without running. Walk up to the end cone, turn around and walk back to the starting cone behind you, turn again and back to the end cone, then turn once more and return back to start cone again so that you walk the 10m walkway 4 times in total. Get ready and START."
- Scoring: Time starts on the signal to start at the start line and terminates once the participant crosses back over the start line after completing the 40m. Each time the participant crosses the 10m, timing is paused whilst the participant turns around the cone and then is resumed once they cross the 10m mark again. The same is repeated for the following turns, and it is stopped once the participant crosses the start line for the final time.The walking speed is automatically calculated in the database as the distance (40m) divided by the time (in seconds).[60]

d. 6-Minute Walk Test: This test measures aerobic capacity and long distance walking activity (in meters).

- Equipment: flat walking area (e.g. track), timer/stopwatch, colored tape to mark boundaries of course or turn points, chair for resting if required.
- Procedure: Participants are asked to walk as fast as they can, without running or putting themselves at risk, for 6 minutes around the track to cover as much ground as possible. Verbal encouragement is given at minute intervals.
- **Tester script:**
  "For this test, do the best you can by going as fast as you can, but do not push yourself to a point of overexertion or beyond what you think is safe for you. Start with both feet on the start line. On start, walk as quickly but as safely as possible, around the track between the marked lines. Continue the course to cover as much ground as possible over 6 minutes. Walk

continuously if possible, but do not be concerned if you need to slow down or stop to rest. The goal is to feel at the end of the test that no more ground could have been covered in 6 minutes. You can sit down to rest if you require. Get ready and START."

- Scoring: The test starts on the signal to start and terminates at 6 minutes. The distance covered over the 6 minutes is recorded in meters (multiply the number of laps participant completed by 37.56, and sum the additional distance covered measured with a digital measuring wheel).

6. Stair climbing test: This is a test of ascending and descending stairs. It records the time in seconds it takes to ascend and descend a 12 step flight of stairs (descend time is calculated by subtracting the ascending time from the total time to ascend and descend the steps).
   A. Equipment: 2 timers/stopwatches and flight of stairs. Flight of stairs used for this test is the one closest to the building elevators (without windows) to standardize the steps heights (between 16-20cm). The test should be performed when stairs are free from traffic and external distractions.
   b. Tester: if safety is a concern, the test should not be done. The tester can guard behind/below the participant going up the stairs or stay on the starting platform (Allow participant to climb a couple of steps before testing to assess for safety). The use of a handrail is mandatory. The use of walking aid is permitted.
   c. Scoring: timing starts on the signal to begin (on both timers/stopwatches). One timer/stopwatch will record the time the participant finishes ascending the steps, and the other one will record the total time when participant completed ascending and descending the steps. The participant can stop and rest during the test if needed, but the time keeps going.
   d. **Tester Script**
   "You will ascend the flight of stairs as quickly as possible but in a safe manner. Start with both feet on the bottom landing. On begin, go to the top of the stairs as fast but as safe as you can. Always use the handrail. Ready, begin".
   In case the standard stairs cannot be used, the windowed stairs can be used, and this information needs to be reported on the form (the number of steps and the step height in cm). The instructions to use the windowed stairs are: the tester should ask the participant to ascend and descend the first flight of steps (12 steps) as if they were going from the 2nd to the 3rd floor of the building. Time is recorded in the same way as described above, and during follow-up visits, the same steps need to be used for test completion.I.[61,62]

7. Sitting/Rising Test: measures the subject's ability to sit and rise from the floor. The test is administered in a 1.52 X 1.52 m non-slippery mat. For this test, subjects should be wearing clothing that does not restrict body movement. Subjects are asked to sit down on the mat and rise to an upright position using minimal support. The number of supports used (hands, forearms, knees, side of the leg and/or of the foot) and the number of unsteadiness (partial loss of balance) are recorded for each component: sitting and rising. One practice trial is done before the test, and tester can provide instructions to improve subject's performance. In case the

subject refuses to do this test or tester deems unsafe, the test is not completed and this information is recorded.

- **Tester Script**
  "Without worrying about the speed of movement, try to sit and then to rise from the floor, using the minimum support that you believe is needed".[63]

8. Muscle Strength Tests:
   a. Hip Abductors Strength Test: measured while the patient is side lying. The tester uses a measuring tape to measure the moment arm, which is the distance in centimeters between the greater trochanter and 1 inch above the lateral epicondyle. The hip is placed in slight abduction and extension while the knee extended. A hand-held dynamometer is placed at the line (1 inch above the lateral epicondyle) and the subject is asked to push up against the dynamometer as hard as he/she can for 5 seconds. Tester is trained to match the force produced by the subject, in other words, the tester cannot exert more force than the subject and "break" their maximum voluntary contraction. The force (in kg) produced is recorded in the database. Verbal encouragement is given during the test, and the test is performed on both lower extremities.[64]
   b. Quadriceps Strength Test: is measured bilaterally using a maximum voluntary isometric contraction (MVIC) of the quadriceps muscle. Subject sits on an isokinetic dynamometer (Biodex System 4 Pro) with the dynamometer force sensing arm secured to the ankle. The knee being tested is positioned in 70 degrees of flexion. The tester fastens the subject's body with belts across the chest and hip while sitting on the chair to assure body stability and to avoid compensatory muscle force from other muscle groups. The subject is asked to exert as much force as possible while extending the knee against the force sensing arm of the dynamometer. Subject performs 3 warm-up trials: one at 50%, one at 75% and one last at 100% of the maximal voluntary isometric strength. Five trials at 100% MVIC are performed with 1-minute rest for recovery in between trials. Data is processed at a later date by a research assistant. The MVIC is measured in *newton-meter* (Nm). The highest value of each curve sustained for at least 3 time points is recorded as the MVIC for that curve. Three of the five trials with the highest values are recorded in the database.[64,65]

9. Real-time physical activity (SW monitor): measures physical activity in real-time. At the end of the baseline testing session, a clean monitor and armband are given to subjects to be worn on the back of the left arm for 8 days. The subject is instructed to remove the monitor during sleep and during water activities (e.g. shower, swimming). The subject also receives a daily log, instructions form (**APPENDIX: A. Study Forms**: Physical Activity forms) and a pre-stamped envelope with to return the monitor and daily log to the research team. Subject signs the receipt form, where monitor serial number is written.[36,37]

The in-person eligibility screening and baseline visit are estimated to last approximately 3.5 hours.

6. RANDOMIZATION

The study coordinator performs the randomization through a web-based computer system at the end of the baseline visit, thereby preserving allocation concealment. Once group assignment is established, the coordinator discloses this information to the subject followed by instructions on how to proceed with the study. Subjects randomized to either the clinic-based or the community-based exercise programs are scheduled for the first exercise visit only after physical activity data is collected (~8 days).

Patients are randomized using a 2:2:1 allocation ratio to receive one of the two exercise interventions as compared to usual medical care. This study uses an adaptive randomization approach with minimal sufficient balance algorithm [66,67] to minimize imbalances in important prognostic variables at baseline including gender, age, BMI, physical function, and knee range of motion. These measures have been selected due to their strong associations with the study outcomes of physical function and activity. Allocation is assigned based on the instantaneous imbalances instead of being generated as a fixed list prior to the beginning of the trial.

7. MASKING

While the treatment assignments clearly cannot be masked to the patient, several steps are taken to decrease bias: (1) Subjects are masked from in-depth information of intervention in the other group and are instructed not to discuss any aspects of the treatment with the testers; (2) The treating physical therapist and the leader of the group exercise are masked to subjects' performances on outcome measurements; (3) The testers are masked to subjects' group assignments. Despite the efforts to keep tester unmasked, the research team acknowledges that break of blinding may occur and a protocol deviation is completed reporting a break of blinding when that happens. To ascertain if the testers are kept masked throughout the study, at the end of the study the tester will try to guess group assignment.

8. STUDY INTERVENTIONS

8.1. Clinic-based Individual Outpatient Rehabilitative Exercises

The exercise program used in this group has been shown to be safe and feasible and combines the best research evidence. Subjects participate in 12 supervised sessions of exercise (60 minutes each) followed by a home exercise program. The 12 sessions are supervised by a physical therapist during 3 months in the following schedule: 2 sessions per week during weeks 1-3; 1 session per week in weeks 4 to 7; and 1 session every 2 weeks for the last two visits. This gradual weaning is designed is allow enough time for the subjects to learn the exercises and increase adherence with the home exercise program. Subjects are instructed to start home exercise after the 3rd week of the supervised program in a way that they exercise twice a week (either supervised exercise in the clinic or at home) during the 3-month intervention phase.

Treatment sessions utilize a pragmatic approach and include: (1) warm-up with stretching of lower extremity muscles and range of motion exercises; (2) moderate to vigorous intensity strengthening exercises of the major

lower extremity muscle groups (knee extensors, knee flexors, hip extensors, and hip abductors); (3) moderate intensity aerobic training using a treadmill or exercise bicycle; (4) functional activities such as getting up from and sitting down in a chair, squatting, walking in place, kneeling, stair climbing and dancing; and (5) agility and balance exercises. All 5 components of the exercise program are used with each subject because patients with physical limitations post-TKR are all affected to varying degrees by these impairments. Exercises are performed in both legs and are initially performed at low intensity and progressively increased to the target level, as long as subjects do not experience increased pain, effusion, or decreased range of knee motion. Treatment sessions utilize a pragmatic approach, allowing the physical therapist to make modifications accordingly to subjects' needs. Individualization of exercise occurs in the selection of what exercises are emphasized in each component and the rate of exercise progression.

8.2. Community-based Group Exercise

Participants randomized to this group attend 45-60 minutes group exercise classes for older adults at local community senior centers at the same frequency/duration as the clinic-based exercise group; 2 times per week for 3 months. The size of group exercise classes is variable but generally larger than 4 participants. The research participants attend classes along with non-research participants who are members of the community centers. The community senior centers participating in this study are the Jewish Community Center (JCC) – Squirrel Hill, JCC – South Hills, the Vintage Senior Center and the Monroeville Senior Citizen Center. In these centers, there are target classes that have the same elements targeted by the physical therapist in the clinic-based exercise group (strengthening, balance and coordination exercises). The target classes at JCCs are the SilverSneakers Circuit classes, at Vintage are the Enhanced Fitness classes and at Monroeville center are the SilverSneakers Experience classes. The classes consist of a variety of exercises designed to increase general muscular strength, improve cardiovascular fitness, joint mobility, balance, and daily living skills. No specific body region is targeted with these exercise classes. Some of the exercises include: partial squats, leg and knee extension/flexion, elastic tubing or free weight for strength training of the upper arm and chest muscles, coordination drills with a gym ball such as bouncing, throwing and catching, and low-impact cardiovascular exercise using treadmill, bikes or aerobic series on the floor. The classes are taught by trained physical fitness instructors. Subjects randomized to this group are allowed to do the activities provided in the center they chose (i.e., participate in other classes, have lunch and other social events, use the fitness center), and the activities are tracked by the research personnel.

8.3. Usual Medical Care (waited-list control group)

The usual medical care group does not receive any attempt from the research team in a way that would interfere with their activities up their 6 months follow-up visit. At this visit, subjects randomized to clinic-based individual outpatient rehabilitative exercise group or to the community-based group exercise and will be exercising for 3 months.

9. EXERCISE COMPLIANCE

The exercise procedures of the study are being monitored by the research team on a regular basis through phone calls. Every month and a half, the research team contacts the subjects to track their compliance with the exercise program, collect information on additional exercises done outside the ones proposed by the study, and on adverse events. This information is recorded using the Health Status Update form in the database.

For the individualized outpatient rehabilitative exercise group, exercise is monitored using the daily logs completed by the physical therapist. Subjects are also asked to complete the home exercise log form, where they mark the exercises done at home. For the community-based group exercise, the research team contacts the community centers participating in the study to get a monthly report of subjects' attendance (electronic swipe of the center's cards, and a copy of the class signing sheet – at the JCC).

10. FOLLOW-UP PHONE-CALLS

The phone calls target dates are calculated to take place at 1.5 months (45 days), 4.5 months (105 days) following the original randomization date. The waited-list usual care group has a follow-up phone call 45 days after the second randomization date. The window of time to have these phone calls is 7 days prior or after the target date. The goal of these calls is to keep the subjects engaged in the study. [68,69] During the phone calls, the coordinator obtains information on health status, adverse events and co-interventions (**APPENDIX: A. Study Forms**: Health Status Update form). Exercise compliance is also monitored during these phone calls with questions regarding frequency and type of exercise that subjects are doing and it is recorded on the health status update form.

Subjects are asked by the research coordinator to contact the research personnel in case any of any change in health status occurs during their participation in the study.

11. FOLLOW-UP IN-PERSON TESTING

The target date to schedule the follow-up visits are calculated to take place 90 days (3 months visit) and 180 days (6 months visit) after the date of randomization. There is also an additional follow-up visit for the Usual Medical Care group 270 days (9-month visit) after the randomization date. The ideal window of time for the follow-up visits to take place is 7 days prior or 21 days after the target date. The trial coordinator attempts to contact subjects prior to the opening of the window to ensure that the visit is successfully scheduled within the specified 4-week window.

During the scheduling call, the subject is asked to bring an updated list of the medications (including the ones over the counter), reading glasses if needed, and exercise clothes. Subjects in the individualized physical therapy group are instructed to bring the home exercise log to this visit.

11.1. Three and Six Months Testing Sessions (and Nine Months for the Usual Medical Care Group)

The follow-up visits are estimated to last approximately 3.5 hours.

The trial coordinator greets the subject and walks him/her to the clinical evaluation room. During the initial part of this visit, the research coordinator is responsible for completing the following forms:

- Health Status Update Form: measures health status, adverse events, exercise program compliance, and co-interventions.
- Global Rating of Change: this form measures subjects' overall knee condition from the time they started the research study (baseline) to the current time point.
- Medication form: this form tracks any changes in subjects' medication list since enrollment in the study.

During the second part of the visit the tester is responsible to instruct the subject on how to complete the following self-reported measures:

- WOMAC
- CHAMPS
- RAND 36
- ASES
- Falls History form (same version as the one completed during the phone calls at 1.5 months)
- CES-D10
- Beck Anxiety Inventory
- Tampa Scale for Kinesiophobia
- Coping Strategies Questionnaire
- EQ-5D
- COPM
- PROMIS Physical Function & Pain Interference

Once the self-reported information above is completed, the tester starts to perform the following tests and record onto the clinical examination form:

- Passive knee flexion and extension, knee extension lag, single-leg balance test, time up and go test, repeated chair stand test, stair climbing test, time to walk 4-meters, 40-meter fast-paced test, 6-minute walk test, sitting/rising test, and muscle strength. During the clinical examination test, the tester measures the subject's height, weight, and blood pressure. If the tester deems any of tests unsafe for the subject to perform, the test is not completed and this information is recorded. In case the subject did not perform the strength tests in the baseline visit, he/she should not do it in the follow-up visits.
- Real-time physical activity (SW monitor): an activity monitor is given to the subject at the end of the visit. Instructions are the same as in the baseline visit.

At the end of this visit, the research coordinator reminds the subject about upcoming time point according to group assignment:

- Individualized physical therapy: subjects are instructed to continue the exercises learned in the sessions at home for the next 3 months. A new home exercise log is given to them so the research team can track their exercises.
- Community group exercise: subjects are instructed to continue attending the exercises classes during the next 3 months.
- Usual care wait-listed group: subjects are instructed to continue their regular activities up to their 6-month follow-up visit.

During this final part of the visit, the trial coordinator loads the subject's WePay card to reimburse for travel and time. A receipt is printed for the trial coordinator's records and a receipt with the subject's name is printed and given to the subject for his/her records.

11.2. Wait-list Usual Care group at the 6 months visit

At the end of the 6 month testing session, subjects in the wait-list usual care group are randomized to one of the two exercise groups: 1) individualized outpatient rehabilitative exercise group; or 2) community-based group exercise. The trial coordinator checks if the subject's health history has changed in the past 6 months to ensure continued eligibility. If the subject continues to be eligible to exercise, the trial coordinator gives him/her instructions to continue for the next 3 months according to the group to which they were randomized.

The second randomization (for the wait-list group only) follows the same procedures described above. Based on the second randomization date, these subjects have one follow-up phone call (45 days) and one follow-up testing visit (90 days).

12. CLINICAL MEASURES

12.1. Outcome Measures

Table 1 describes the clinical measures completed at each time point.

12.1.1. Primary Outcome Measure

The primary outcome measure is physical function at the 3-month follow up assessed by a patient-reported survey, the WOMAC-PF. The WOMAC-PF consists of 17 items related to physical function. Each item is scored on a 5-point Likert-type Scale with descriptors from 0-4 (none, mild, moderate, severe and extreme). The WOMAC PF is calculated as the sum of the items, for a maximum total score of 68. Higher scores indicate worse functional limitations. Reliability and validity of this instrument have been established.[42–44]

12.1.2. Secondary Outcome Measure

Secondary outcomes of physical function comprise a battery of performance-based tests that include: Gait speed assessed by the 40-meters fast-paced walk test; Chair rise test that times participants during 5 repetitions of rising to a full upright position and sitting back down in the chair (18" chair without armrests) without

assistance; Single leg stance test that records the time of balancing on one leg while keeping the hands on the hips. The test lasts up to 60 seconds and is stopped if the swing leg touches the floor, support foot moves on the floor, or arms swing away from the hips; Stair ascend/descend test that times participants while climbing up and down a set of 11 stairs (30 cm depth, 17 cm height) using a handrail on the preferred side; Six min walk test that assesses the distance covered while walking during 6 min on an unobstructed, rectangular circuit (marked in meters)[60-62]; Sitting-rising test that assesses the ability of participants to sit and rise from the floor.[63] Results of these tests are combined using a composite score formed with unit-weighted z scores of constituent tests to provide a more stable measure of the subject's underlying functional performance.

Additional secondary outcome includes physical activity measured using the SenseWear Minifly (SWM) (Body Media Inc, Pittsburgh PA) and the Community Healthy Activity Model Program for Seniors questionnaire (CHAMPS). The SWM provides real-time measures of physical activity in subjects' homes or communities during normal activities of daily life. The SWM has good reliability and validity. [37] Subjects are instructed to wear the SWM on the back of the left arm during wake time (they are asked to wear the monitor on the arm from the time they get up in the morning to the time they go to bed), except during shower and water activities. Moderate-intensity activities are recorded along with data captured on sedentary behavior and physical activity performed at light intensity (up to 3METs). The CHAMPS assess self-reported physical activity, and it is a reliable, valid and responsive instrument.[46] It assesses activities such as hobbies, work- and social-related activities, walking, swimming, dancing; and complements the information obtained from the SWM.

Table 1. **Clinical measures completed at each time point.**

Questionnaire/Form	Ba	Call 1.5 m	3 m	Call 4.5 m	6 m	Call 7.5 m	9 m
Demographics	X						
Medical History form	X						
Cumulative Illness rating scale	X						
Medication	X		X		X		X
WOMAC	X		X		X		X
Inclusion/Exclusion Criteria form	X						
CHAMPS	X		X		X		X
RAND 36	X		X		X		X
Arthritis Self-efficacy	X		X		X		X
Falls history	X	X	X	X	X	X	X
CES-D 10	X		X		X		X

Beck Anxiety Inventory	X		X		X		X
Tampa Scale for Kinesiophobia	X		X		X		X
Coping Strategy Questionnaire	X		X		X		X
COPM	X		X		X		X
Clinical examination form	X		X		X		X
Physical Activity Data	X		X		X		X
EQ-5D	X		X		X		X
PROMIS Physical Function & Pain Interference	X		X		X		X
Global Rating change form			X		X		X
Health Status Update		X	X	X	X	X	X
Attrition			X		X		X
Adherence to Intervention		X	X	X	X	X	X
Adverse Events		X	X	X	X	X	X
Co-interventions		X	X	X	X	X	X

12.1.3. Other Measures

At baseline, data are collected on demographics and biomedical characteristics and comorbidity. These data are used to characterize the sample. Demographics and biomedical characteristics include age, gender, race, education, BMI, self-rated health (excellent, good, fair, poor, or bad), discharge placement, number of prior rehabilitation sessions, surgical technique, and surgeon experience. Comorbidity is assessed by the Cumulative Illness Rating Scale (**APPENDIX: A. Study Forms**).

Additionally, data on medication, psychosocial factors, and impairments of the lower extremities are collected at baseline and each in-person follow-up visit to test potential predictors or modifiers of treatment response. Medication information includes medication prescribed and over-the-counter used for pain. Psychosocial Factors include fear-avoidance beliefs, anxiety, self-efficacy, depression, and pain coping. Lower extremities impairments knee pain, knee range of motion, and muscle strength (described in Section **5.BASELINE TESTING**).

Safety and exploratory outcomes include the measures of harm assessed by adverse events and measures of study engagements including attrition, adherence to intervention, and participation in co-interventions, respectively. Adverse Events include, but are not limited to, changes in knee symptoms, falls, hospitalizations,

and TKR on the other knee. Attrition is defined as the number of patients dropping out of the study in each group. Adherence to intervention is estimated by the proportion of sessions attended in each group and the proportion of patients missing each session. Co-Intervention is defined as additional treatment sought besides the ones prescribed by the study.

13. ADVERSE EVENTS

13.1. Management

The occurrence of adverse events is monitored for each subject on an ongoing basis throughout the study. Reporting of adverse events in the context of the proposed program of research occurs according to the following definitions:

- Serious. This adverse event is fatal or life-threatening; requires hospitalization, or produces a disability.
- Moderate or greater severity. This adverse event requires medical evaluation and/or medical treatment; or is a serious adverse reaction.
- Unexpected. This event is not identified in nature, severity or frequency in the IRB-approved research protocol or informed consent document.
- Associated with the research intervention. There is a reasonable possibility that this event may have been caused by the research intervention (i.e., a causal relationship between the event and research intervention cannot be ruled out by the investigators).

13.2. Report

All adverse events that are (a) unexpected; (b) of moderate or greater severity; and (c) associated with the research intervention are reported to the IRB. In the case of a serious adverse event, an emergency meeting of the investigative team is called. At the time of this meeting, a determination is made as to whether the trial should be prematurely interrupted. Expected adverse events; unexpected adverse events of minor severity; or adverse events which are determined by the PI to be unrelated to the research intervention are not reported to the IRB. These events are reported to PCORI during the annual report.

All adverse events are reported according to the following timeline: If the event is fatal or life-threatening, the report to the IRB and the PCORI occurs within 24 hours of the event. If the event is unexpected, and of moderate or greater severity (but not fatal or life-threatening), and associated with the research intervention, it is reported to the IRB and the PCORI within 10 calendar days of the reaction. The IRB and the PCORI are also notified as soon as possible of major disputes between the PI and/or project staff and a research subject or between research investigators (including research staff) involved in the proposed program of research if the resolution of the dispute is or will be problematic. If an unexpected adverse event occurs, the PIs re-assess the risk/benefit ratio of the study and submit any modifications deemed necessary to the IRB and the PCORI for approval.

14. STUDY COMPENSATION

This study is using the UPMC WePay System (https://wepay.upmc.com/WP/) to compensate subjects for their participation. Staff online training is required.

During the baseline visit, the coordinator searches for or adds subject information to the WePay System. Subjects need to be registered in the WePay system so compensations can be processed. Once the subject is registered in the system, the coordinator assigns one pre-paid WePay card to the subject (need card verification value information and 4-digit PIN) and loads US$40.00. Two receipts are printed. The one with subject's name is given to the subject. The other, with subject's study ID, is kept by the research team.

Subjects were compensated for their time at all testing sessions (baseline, 3 and 6 months testing visits).

15. DATA ANALYSIS AND SAMPLE SIZE JUSTIFICATION

15.1. Primary Hypothesis

The primary hypothesis is that subjects in Groups 1 and 2 will demonstrate better physical function and physical activity as compared to Group 3 (usual medical care). Analysis for this hypothesis will use an intention-to-treat approach. The primary outcome for this analysis is the WOMAC-PF subscale at 3 months. This analysis will use contrasts from a linear mixed models analysis for 3 and 6-month function controlling for baseline function and the randomization covariates (age, gender, BMI, physical function, ROM). We will first explore the intervention by time interaction and then proceed to a main effects model with only group and time. Our primary interest is the 3-month comparison between the clinic-based individual outpatient exercise and the community-based exercise groups. The linear mixed models allow maximization of the number of individuals used for the analyses, as a person can contribute information at both time points, or just at one time point. To test if the improvements in outcomes are sustained, we will use contrasts from the linear mixed model at 6 months. For the secondary outcomes of physical function (battery of performance-based tests such as walking ability, chair rise, single leg stance, stair climbing, six-minute walk, and sitting-rising) and physical activity, analyses are performed as described above, one for each measure. We will combine the score of the performance-based tests using a composite score formed with unit-weighted z scores of constituent tests to provide a more stable measure of the subjects' underlying functional performance.[68]

Sample size and power calculations for primary analysis were based on the primary endpoint of WOMAC-PF subscale at 3 months. We propose to recruit 240 subjects (96 in each exercise arm and 48 in the usual care arm) to allow approximately 86 subjects in each exercise arm and 43 in the usual care arm available for a complete case analysis (assuming 10% attrition at 3 months). With an alpha level of 0.05, 2 tails test, a sample size of 172 (n=86 in each exercise group) will provide 81% power to detect a difference of 3.3-point difference between the two exercise groups in WOMAC-PF (SD of 7.7)[35]. The sample size of 43 in the usual medical care group will provide 80% power to detect a difference of 5.2-point difference in WOMAC-PF between the usual medical care group and any exercise group. Power analysis was conducted in NCSS/PASS (PASS 12 Power Analysis and Sample Size Software (2013). NCSS, LLC. Kaysville, Utah, USA, ncss.com/software/pass).

15.2. Secondary Hypothesis

The secondary hypothesis is that a group of baseline biomedical and psychosocial measures will be associated with treatment response. For this analysis, each subject will be classified as a responder or non-responder based on a minimum change score of 20% in both the WOMAC-PF and the composite score of functional performance at 3 months, thus yielding a binary outcome. Baseline variables will be summarized separately for responders and non-responders. Unadjusted odds ratios will be estimated using univariate logistic regression. To consolidate potential predictors, we will test for collinearity among baseline variables that are associated with the response. Baseline measures associated with response at the $p<0.15$ level in unadjusted models will be added to multivariable logistic regression models to assess predictors of treatment response. We will limit the number of predictors going into any one model to no more than one predictor per 10 responses (or 10 non-responses, whichever is less); if more variables are significant, the model will be limited to the most significant variables, after adjusting for those deemed a-priori to be clinically significant.[74]

Power calculation for the secondary analysis is based on the binary outcome of 20% change in physical function. Participants initially randomized to one of the exercise arms and those in the usual care group later randomized to the exercise arms will be included in the analysis for a total of approximately 200. If the expected response rate ranges between 50% and 60%, we would be able to detect an odds ratio of 2.2 to 2.4 with 80% power assuming a binary predictor with 50% split in the sample.

15.3. Exploratory Aim

For the exploratory aim, we will calculate dropout rates as proportions of subjects randomized and as a cumulative probability of remaining in the study using survival analysis techniques, such as the product-limit estimator. These statistics can be estimated at various times following randomization and take into account when dropouts occur. Descriptive statistics will be used for reporting and evaluating implementation of the exercise protocols including the proportion in attendance for each session and the average number of sessions attended by group. To assess the impact of non-adherence, we propose to explore using instrumental variable (IV) methodology to estimate the efficacy of our interventions in the presence of non-adherence.[70,71,72] We propose to use the two-stage IV methods which can be easily implemented using simple linear structural models for the effect of sessions attended on the primary outcome of function. We will also calculate the 6-month incidence (and 95% CI) of individual adverse events by organ system and relatedness to the study for each group. We will estimate the incidence of adverse events with a specific focus on those deemed definitely, probably, or possibly related to interventions. For adverse events, clinical judgments will be considered more important than statistical testing.

We also propose sub-group analyses to explore heterogeneity of treatment effects using several potential moderators of treatment response measured prior to randomization that may either potentiate or attenuate the effects of our intervention (e.g., patient gender, age, BMI, range of knee motion). These are the same prognostic variables used for the adaptive randomization in the study. We will examine interactions between the treatment and modifier being considered. Even if the interaction is not statistically significant, we would estimate the treatment effects stratified by age along with the 95% confidence intervals to look for consistency of treatment effects.

16. DROPOUT AND MISSING DATA

We estimate the attrition to be 10% at 3 months follow-up and 15% at 6 months. We will compare baseline characteristics between patients with and without the assessment at 3 and 6 months to assess potential biases in the complete case analysis. We will also try to obtain reasons for study drop-out to assess missing data mechanism (missing completely at random, missing at random, non-ignorable missingness). We will use several missing methods for imputing data and re-analyze using intention-to-treat (as randomized) to assess the impact of missing data on our conclusions as recommended. [76] We will first use multiple imputations (with M=10 imputations) which assumes the data are missing at random. Since the data could be missing not at random, we will use another approach of assigning the lowest observed scores for missing values differentially by treatment group (non-ignorable missingness). The approach assumes the missingness is directly related to the value of missing data, i.e., the people who are missing data on function have worse function scores (did not come in for assessment because function was worse). Results of all approaches to missing data will be presented in the primary paper for our study. If our significance and interpretation of our treatment effect vary depending on the method of imputation, we will view any conclusion cautiously.

17. RECORD KEEPING

The majority of the forms and questionnaires used in this study are entered directly into the database. The hard copy of the forms completed on paper are being stored in subject's file and kept in a locked file cabinet in the trial coordinator's office. Only study personnel has access to these files.

Table 2 (below) shows the type of data storage for all forms. The telephone screening is completed in paper form, and only the recruitment source and the inclusion/exclusion criteria are entered in the database (without the subject's name and telephone number). Telephone Screening forms of subjects who are deemed eligible are kept in locked file cabinet separately from research data collection forms.

A copy of the Informed Consent signed by the subject and by the trial coordinator is kept in a separate locked file cabinet in the trial coordinator's office, as well as the Contact Information form.

Subjects bring an updated list of medication they take, including the ones they take over the counter, to all follow-up visits. This information is entered in the study database, but the hard copy is kept in subject's file.

The Inclusion/Exclusion criteria form is completed directly in the database. A copy of the complete form is printed and given to the PI to sign and date. This is done in order for the PI to be aware and to control only eligible subjects are randomized to the study.

The clinical examination form is completed in paper form and is immediately entered into the database by the tester. The hard copy is kept in subject's file.

The EQ-5Dis recorded in paper form. It is kept in subject's file.

Table 2. Type of data storage.

Form Name	Hard Copy – paper form	Electronic Entry
Telephone Screening form	X (used first)	X (entered later)
Informed Consent	X	
Contact Information form	X	
Demographics		X
Medical History form		X
Cumulative Illness rating scale		X
Medication	X (used first)	X (entered later)
WOMAC		X
Inclusion/Exclusion Criteria form	X	X
CHAMPS		X
RAND 36		X
Arthritis Self-efficacy		X
Falls history		X
CES-D 10		X
Beck Anxiety Inventory		X
Tampa Scale for Kinesiophobia		X
Coping Strategy Questionnaire		X
COPM		X
Clinical examination form	X (used first)	X (entered later)
PROMIS Physical Function & Pain Interference		X
Physical Activity Data		X
EQ-5D	X	

Physical Activity		X
Randomization		X
Global Rating change form		X
Health Status Update		X

The database is web-based for direct data entry, where subjects are identified by a study ID (i.e. KTX000), and no personally identifiable information is stored in it or used in any of the analyses.

18. DATA MANAGEMENT PLAN

Data management is overseen by the PI, Dr. Moore and Dr. Gil, and is coordinated by the Center of Research on Health Care Data Center (DC). The DC created an electronic System for Data Management (eSYSDM) for data collection, tracking, follow-up, reporting, and analysis need. The research coordinator obtains the patient's initials once a patient is recruited and deemed eligible for the study to initiate inclusion in the in the tracking system. The tracking system monitors enrollment and tracks follow-up rates and the data entry process, providing up-to-date status reports. The eSYSDM includes verifying the data, out of range data checks, and repeated evaluation of data process, eliminating the possibility of most incorrect entries and preventing extensive recoding and cleaning by the statistician.

The primary method of data collection is through the database. However, if access to the internet is disrupted, paper forms are available to ensure data collection. All data collected in paper forms are stored in the subject's research chart identified by their ID. In this case, coordinator contacts the database programmers via telephone to obtain the subject's group assignment.

19. QUALITY ASSURANCE

To ensure data quality and integrity we are using standard methods of data collection and recording, have formal staff workshops on research integrity, document computer operations and data editing procedures, and have regular meetings with project staff to review any changes in procedure. The electronic forms are maintained by the DC on a local network in a relational database. The DC performs routine data edit checks for consistency. Once data are edited, temporary files will be merged to generate the final files that will be used for data analyses. All files are backed-up daily and archived weekly.

Dr. Gil and the trial coordinator check all data collected every 3 months to certify that data is being collected and maintained properly. Equipment, such as the Biodex System, requires calibration. Research staff members are trained to calibrate the machine on a regular basis: the Biodex is calibrated every 3 months

20. DATA SAFETY AND MONITORING BOARD

Study personnel decided to have a Data Safety and Monitoring Board (DSMB) to promote quality of monitoring the study. Since PCORI does not have its own DSMB Guidelines, this board follows the National Institute of Health (NIH) Guidelines.

The DSMB meetings occur every 6 months, and the reports are part of the annual reports the principal investigator sends to PCORI.

The DSMB reviews the accumulated study data for participants' safety, study conduct, and progress, and makes recommendations about study continuation, modification, or termination. The DSMB is responsible for defining its deliberative processes, including event triggers that would call for an unscheduled review, stopping guidelines, unmasking and voting procedures. The DSMB is also responsible for maintaining the confidentiality of its internal discussions and activities as well as the contents of reports provided.

A narrative summary and tables are compiled prior to each DSMB meeting and include the following:

Open Session:

Table 1A. Screening Information and Reasons for Ineligibility
Table 1B. Enrollment (Consented) and Randomized by Month of Study
Figure 1. Comparison of Target to Actual Enrollment by Month
Table 2. Reasons for Screen Failures
Table 3. Participant Enrollment and Status
Table 4. Demographic and Key Baseline Characteristics by Group
Table 5. Adverse Events: Level of Severity
Table 6. Adverse Event Details
Table 7. Serious Adverse Events
Table 8. Deaths
Table 9. Protocol Deviations
Table 10. Summary of Missed Visits
Table 11. Exercise Compliance

Closed session: Relevant data displayed by intervention arm. Masked research personnel is dismissed at this point of the meeting.

CONSORT Diagram (by intervention arm)
Table 12. Demographic and Key Baseline Characteristics (by intervention arm)*
Table 13. Adverse Event Details (by intervention arm)
Table 14. Protocol Deviations (by intervention arm)

Closed Executive Session: Only DSMB members to ensure complete objectivity as they discuss outcome results, make decisions, and formulate recommendations regarding the study.

After the meeting, the research team prepares the minutes to be approved by the DSMB. All reports and approved minutes are kept electronically in the study folder on the School of Health and Rehabilitation Sciences network system. DSMB members are compensated for the meetings.

DSMB Members:

- Julie Fritz, PT, Ph.D. – Professor at the Physical Therapy/Orthopedic Surgery Operations, University of Utah (chair of the board)

- David Sinacore, PT, Ph.D. – Professor at the Physical Therapy/Medicine, Washington University School of Medicine in St. Louis

- Margaret Conroy, MD, MPH – Assistant Professor at the Medicine, Epidemiology, Clinical Translational Science, University of Pittsburgh

- Subashan Perera, Ph.D. – Associate Professor at the Medicine, Co-Director & Senior Statistician PEPPER CENTER

21. HUMAN SUBJECTS

21.1. Institutional Review Board (IRB) Review and Inform Consent

This protocol and the informed consent document and any subsequent modifications will be reviewed and approved by the IRB (PRO14080261). A signed informed consent form is obtained from all subjects (**APPENDIX D.**

Informed Consent). The consent form describes the purpose of the study, the procedures to be followed, and the risks and benefits of participation. A copy of the signed informed consent form is given to the subject.

21.2. Risks/Benefits Assessment

All evaluations are conducted for the purposes of the present research only. Research data comes from an in-person evaluation of subjects such as the history (e.g., demographics, biomedical factors, discharge placement, prior rehab), physical examination (knee range of motion and muscle strength), and research questionnaires (querying about their functional limitations, pain, physical activity) administered by project staff. Performance-based measures are used to collect information regarding lower extremity physical function. Real-time physical activity data is collected during a week in a free-living condition. While participating in this clinical trial the subjects are able to take their regular medications and therefore the proposed clinical trial will likely not affect the daily lives and the management of other medical conditions.

21.3. Potential Risks

The risks to the subjects are minimal. It is common for individuals to experience muscle or joint soreness in their lower extremities following functional testing or exercise intervention. This muscle or joint soreness typically occurs within 48 hours after physical activity but usually resolves within 1-2 days. Additional risks associated with exercise may include tripping and falling, or an exacerbation of the subject's knee pain and inflammation. Because subjects participate in aerobic exercises (treadmill walking or stationary bicycle), there is a rare risk that subjects may experience chest pain, dizziness, shortness of breath, or a heart attack.

21.4. Risk Management and Emergency Response

To reduce risks, all testing and treatments are administered by health professionals who monitor vital and clinical signs during the performance of exercises. To safeguard against the occurrence of injuries or falls, the exercises are performed under the close supervision of a physical therapist or leader of the group exercise class. To safeguard against the risk of a heart attack, we exclude subjects who have uncontrolled cardiovascular disease or hypertension and also subjects who have absolute or relative contraindications to exercise testing, as established by the American College of Cardiology/American Heart Association. To minimize the risk of muscle or joint soreness, subjects progress through study exercises only if they do not experience increased pain, joint effusion, or decreased range of motion. Signs and symptoms of knee inflammation are monitored during the study to determine if training activities exacerbate these conditions. Training activities associated with increased signs and symptoms of inflammation are suspended until symptoms resolve. If symptoms persist, the subject is referred to the study consultants or the patient's physicians. In addition, the exclusion criteria provide that individuals who are prone to falling or have progressive motor disorder will not participate in the proposed study. All subjects are informed of any potential risks prior to their participation in any study procedures and are told that they are free to withdraw from the study at any time. Although no other risks are anticipated, subjects will be informed if any new information arises regarding risks of participation that may affect their decision to continue in the study. Emergency medical treatment for injuries solely and directly related to participation in this research study are provided by the hospitals affiliated with the University of Pittsburgh Medical Center (UPMC). It is possible that the hospital may bill the subject's insurance provider for the costs emergency treatment, but none of the costs will be charged directly to the subject.

21.5. Confidentiality

Patient confidentiality is maintained throughout the study. The risk of breaching subject confidentiality is minimized by using a web-based system of data entry. The data is directly entered into a computer at the time of the interviews. A relational database is stored on a local network where only select research team members have access. The Electronic System for Data Management elaborated in conjunction with the Data Center (http://www.crhc.pitt.edu/DataCenter), is stored on a local network where only select research team members have access to the database. All files are backed-up daily and archived weekly. The weekly data are stored in a safety deposit, off-site (> 1 mile off campus). The files are maintained for 1 year until the data are erased. All study subjects are assigned unique study identifiers that appear on all data collection instruments, tapes, documents, and files used in the statistical analysis and manuscript preparation. Only limited team members have access to personal information needed for tracking and informed consent. No personal information concerning study participants will be released without their written consent.

21.6. Potential Benefits of the Proposed Research to Human Subjects and Others

The potential benefits of this research include improvement in physical function and increase in physical activity after participation in the exercise program, and thus potential benefits on overall health. The potential benefits, therefore, outweigh the minimal anticipated risks to participants.

21.7. Importance of the Knowledge to be Gained

Our study is designed to test the hypothesis that exercise at later stage post TKR can overcome the functional limitations experienced by these patients. If these limitations are overcome, future disability may be prevented. Moreover, by increasing physical activity, our study may directly impact the general health of subjects following TKR. Additionally, this study will inform the management of subjects post TKR and the design of public health programs to extend the number of years free of disability in this population.

22. STUDY ORGANIZATION AND ADMINISTRATION

22.1. Administration/ Research Personnel

- Sara R. Piva, PT, PhD, Study Principal Investigator.
- James J. Irrgang, PT, ATC, PhD, Co-Investigator
    a. Liaison between the study personnel and the surgeon's practices
- Michael Schneider, DC, PhD, Co-Investigator
    a. Collaborates in developing the best practices to engage stakeholders into study implementation and dissemination
    b. Oversees the panel discussions to ensure conducting them in a manner that allows all voices to be heard
- Charity Moore, MSPH, PhD, Co-Investigator
    a. Biostatistician
    b. Responsible for overseeing randomization and all aspects of statistical analysis
- Alexandra Gil, PT, PhD, Co-Investigator
    a. Responsible for the coordination of DSMB meetings and Advisory Panel meetings
    b. Coordinates data management and processing between the PT-CTRC and the University of Pittsburgh Center for Research on Health Care Data Center
- Maria Beatriz Catelani, PT, MS, Trial coordinator
    a. Assists with recruitment
    b. Responsible for enrollment, scheduling of in-person eligibility/baseline and follow-up testing
    c. Tracks participants
    d. Participates in administering tests
- Gustavo Almeida, PT, MS, Trial coordinator
    a. Assists with recruitment
    b. Participates in administering tests (tester)
    c. Checks accuracy of data retrieved from Bodymedia Armband software for physical activity data
- Anthony DiGioia, MD, Consultant

a. Consults any clinical issues that arise with the subjects undergoing research procedures covering both surgical and clinical aspects of subject's needs
b. Helps with recruitment efforts

- Brian Klatt, MD, Consultant
  a. Consults any clinical issues that arise with the subjects undergoing research procedures covering both surgical and clinical aspects of subject's needs
  b. Helps with recruitment efforts
- Gwendolyn Sowa, MD, PhD, Consultant
  a. Consults any clinical issues that arise with the subjects undergoing research procedures covering both surgical and clinical aspects of subject's needs

23. STUDY PLACES

23.1. Community Centers

Four of the largest community centers in Pittsburgh are directly involved with this research study: the Vintage Senior Community Center, in the East Liberty section of Pittsburgh; and the Jewish Community Center (JCC) located in the Squirrel Hill and the one located in South Hills, and the Monroeville Senior Citizen Center. The community-based group exercise arm of this study are conducted at these four local community centers. All four centers are giving our research subjects access and short-membership to their facilities in order to participate in the group exercise classes that they offer to older adults. The Vintage and JCC executive directors have agreed to serve on the community stakeholder Advisory Panel, as well as the exercise instructors.

23.2. PT-CTRC

The Physical Therapy Clinical Translational Research Clinic (PT-CTRC) is serving as the central location for all testing sessions (in-person eligibility, baseline visit, and follow-up assessments) as well as for the treatment sessions for those subjects who are randomized to the individualized outpatient rehabilitative exercise group.

24. RESEARCH ENGAGEMENT PLAN

This study involves several groups or stakeholders who are deeply engaged. We have assembled an Advisory Panel comprised of several stakeholders, each with different perspectives and areas of interest. We meet semi-annually, either in person or via phone conference, throughout the entire 3-year research timeframe. Members of the Advisory Panel have been and will continue to be engaged in order to provide input into the preparation, execution, and translation phases of the study as described below:

PREPARATION PHASE: Patients were involved through informal communication during research participation, structured interviews, and meetings to discuss study design. They have directly influenced the selection of comparators, outcomes, and study design; Providers provided input during study development and helped to shape the usual medical care arm. They provided key input to the individualized outpatient rehabilitative

exercise arm. All providers supported the need to test the effectiveness of exercise at later stages after TKR and the inclusion of a community-based exercise group; the community groups provided input about the community-based group exercise that takes place at the community centers. They have been engaged with the development of the research design by allowing the PI to observe their group exercise classes for older adults, and to meet with the fitness instructors who teach these classes and older adult members of their organizations. The senior fitness instructors at these centers collaborated to develop pragmatic exercise protocols.

EXECUTION PHASE: Patients edited recruitment materials and did a trial-run of study procedures to ensure that the paperless system of data collection is age-appropriate and that the research personnel is well trained. They are also helping to spread the word about our study through their social media contact lists. Patients are instrumental in providing peer-information about the study for potential participants who would like to discuss study participation with someone who has been part of research studies. Patients are also part of a team of Patient Partners who interview subjects who have participated in the intervention arms of the study to collect information on their experiences and suggestions. The information collected during the interviews is key to shaping the delivery of interventions to improve the care and outcomes of patients who undergo TKR. Along with other lay members of the Advisory Panel they also give feedback on any potentially counterintuitive results. Providers, along with patients, were asked to provide input to maximize recruitment and retention, and are helping to interpret research findings from the stakeholder category to which they belong. Three prominent orthopaedic surgeons are actively engaged with the direct referrals of patients who have had TKR. The directors of the community centers, the JCC and Vintage Centers, forward information about our study through their membership email lists, e-newsletters/print newsletters, bulletin boards, and allow us to place informational brochures in their facilities. They also sponsor breakfast meetings where the PI will present information about the study. The community group representatives and payers are asked to provide interpretation of the results from public health, community policy, and health plan perspectives.

TRANSLATION PHASE: We plan specific steps to aid in the dissemination of the research results. Patients will be asked for their input on the development of lay summaries of the study results and will assist with the design and editing of informational booklets and pamphlets for patients who undergo TKR. Providers will help to facilitate presentations to disseminate the research findings at national meetings and conferences with their respective professional associations including the American Academy of Orthopedic Surgery, American Physical Therapy Association, and American College of Rheumatology. The payers will assist with dissemination of the research results to their network providers and work with the PI to organize regional meetings where the findings can be presented to clinicians. Community and advocate organizations will disseminate the research results through email newsletters to their members such as the community centers newsletter and the Arthritis Foundation Magazine.

All stakeholders will be compensated for their collaboration.

25. STUDY OPERATIONS

Any proposed changes to the protocol will be reviewed by the research team and DSMB, and recommendations will be approved by PCORI before implementation. Protocol changes made will be incorporated in the Manual of Operational Procedures (MOP) and Informed Consent document. A record of all changes, including rationale will be kept on file for future reference. Protocol Revisions will be tracked and will be inserted in the MOP, including previous information, the change made, who made the change, and the date the change was made and approved. On each page of the MOP, there will be a version number and date of approval to facilitate tracking the revisions.

26. PUBLICATIONS

Study protocol will be published at the end of the first year of the study in an open-journal such as BioMed Central Musculoskeletal Disorders. This will enable other researchers and funding agencies to see that this type of exercise trial post-TKR is underway, reducing the duplication of research effort and potentially leading to future collaborations with other researchers interested in the same topic. It will also help researchers engaged in systematic reviews to find out our trial, which may reduce publication bias. Lastly, it will provide a mechanism for other researchers with similar research interests to contact the PI about gaining access to more specific research protocols. If the manuscript is not accepted for publication, it will be made available to other researchers upon request.

The investigators will pursue publication of the primary outcomes within 6 months after study completion. Authorship will be determined prior to writing the manuscript and will be based on the relative scientific contributions of the investigators and Key Personnel. All authors will review and approve the manuscript prior to submission for review.

All publications and presentations will be informed to PCORI within 30 days of submission and will include acknowledgement of funding from Patient Centered Outcomes Research Institute (PCORI), CER-1310-06994.

27. AMENDMENTS

Protocol Version	Approved Date
Original Protocol	August 31, 2015
Version 1.1	May 2, 2016

Amendments below are listed beginning with the most recent amendment.

Amendment 1

The overall reason for amendment: To include the Jewish Community Center (JCC) – South Hills and the Monroeville Senior Citizen Center to the community centers providing exercise classes to the community-based group exercise.

Protocol Changes: Addition of the JCC – South Hills and the Monroeville Senior Citizen Center to intervention locations.

Change: Addition of the JCC – South Hills and the Monroeville Senior Citizen Center to intervention locations.

Amendment 2

The overall reason for amendment: To include detailed description of the tests being performed on the clinical examination.

Protocol Changes: No changes to protocol or to procedures.

Change: Detailed description of the tests was included.

Amendment 3

The overall reason for amendment: To clarify rating in the Cumulative Illness Rating Scale form.

Protocol Changes: No changes were made in the protocol or study procedures.

Change: Addition of description on rating the Cumulative Illness Rating Scale.

---

28. REFERENCES

1. Kurtz, SM, Ong, KL, Schmier, J, et al. Primary and revision arthroplasty surgery caseloads in the United States from 1990 to 2004. J Arthroplasty. 2009; 24:195-203
2. Losina, E and Katz, JN. Total joint replacement outcomes in patients with concomitant comorbidities: A glass half empty or half full? Arthritis Rheum. 2013.
3. Stranges E, Russo A, and Friedman B. Healthcare Cost and Utilization Project (HCUP), Agency for Healthcare Research and Quality; Statistical brief #82: procedures with the most rapidly increasing hospital costs, 2004-2007. 2009.

4. O'Reilly, S, Jones, A, and Doherty, M. Muscle weakness in osteoarthritis. Curr Opin Rheumatol. 1997; 9:259-262.
5. Sharma, L. Proprioceptive impairment in knee osteoarthritis. Rheum Dis Clin North Am. 1999; 25:299-314, vi.
6. Sharma, L, Hayes, KW, Felson, DT, et al. Does laxity alter the relationship between strength and physical function in knee osteoarthritis? Arthritis Rheum. 1999; 42:25-32.
7. Slemenda, C, Brandt, KD, Heilman, DK, et al. Quadriceps weakness and osteoarthritis of the knee. Ann Intern Med. 1997; 127:97-104.
8. Do, BT, Hootman, JM, Helmick, CG, et al. Monitoring healthy people 2010 arthritis management objectives: education and clinician counseling for weight loss and exercise. Ann Fam Med. 2011; 9:136-141.
9. Murphy, L and Helmick, CG. The impact of osteoarthritis in the United States: a population-health perspective: A population-based review of the fourth most common cause of hospitalization in U.S. adults. Orthop Nurs. 2012; 31:85-91.
10. Jones, CA, Voaklander, DC, Johnston, DW, et al. Health related quality of life outcomes after total hip and knee arthroplasties in a community based population. J Rheumatol. 2000; 27:1745-1752.
11. George, LK, Ruiz, D, Jr., and Sloan, FA. The effects of total hip arthroplasty on physical functioning in the older population. J Am Geriatr Soc. 2008; 56:1057-1062.
12. Franklin, PD, Li, W, and Ayers, DC. The Chitranjan Ranawat Award: functional outcome after total knee replacement varies with patient attributes. Clin Orthop Relat Res. 2008; 466:2597-2604.
13. Konig, A, Walther, M, Kirschner, S, et al. Balance sheets of knee and functional scores 5 years after total knee arthroplasty for osteoarthritis: a source for patient information. J Arthroplasty. 2000; 15:289-294.
14. Walsh, M, Woodhouse, LJ, Thomas, SG, et al. Physical impairments and functional limitations: a comparison of individuals 1 year after total knee arthroplasty with control subjects. Phys Ther. 1998; 78:248-258.
15. Swanik, CB, Lephart, SM, and Rubash, HE. Proprioception, kinesthesia, and balance after total knee arthroplasty with cruciate-retaining and posterior stabilized prostheses. J Bone Joint Surg Am. 2004; 86-A:328-334.
16. Wada, M, Kawahara, H, Shimada, S, et al. Joint proprioception before and after total knee arthroplasty. Clin Orthop Relat Res. 2002;161-167.
17. Woolhead, GM, Donovan, JL, and Dieppe, PA. Outcomes of total knee replacement: a qualitative study. Rheumatology (Oxford). 2005; 44:1032-1037.
18. Beswick, AD, Wylde, V, Gooberman-Hill, R, et al. What proportion of patients report long-term pain after total hip or knee replacement for osteoarthritis? A systematic review of prospective studies in unselected patients. BMJ Open. 2012; 2:e000435.
19. Noble, PC, Gordon, MJ, Weiss, JM, et al. Does total knee replacement restore normal knee function? Clin Orthop Relat Res. 2005;157-165.
20. Levinger, P, Menz, HB, Wee, E, et al. Physiological risk factors for falls in people with knee osteoarthritis before and early after knee replacement surgery. Knee Surg Sports Traumatol Arthrosc. 2011; 19:1082-1089.
21. Naal, FD and Impellizzeri, FM. How active are patients undergoing total joint arthroplasty?: A systematic review. Clin Orthop Relat Res. 2010; 468:1891-1904.

22. Riddle, DL, Singh, JA, Harmsen, WS, et al. Clinically important body weight gain following knee arthroplasty: A five-year comparative cohort study. Arthritis Care Res (Hoboken). 2012.
23. Westby, MD and Backman, CL. Patient and health professional views on rehabilitation practices and outcomes following total hip and knee arthroplasty for osteoarthritis:a focus group study. BMC Health Serv Res. 2010; 10:119.
24. Rubenstein, LZ, Powers, CM, and Maclean, CH. Quality indicators for the management and prevention of falls and mobility problems in vulnerable elders. Ann Intern Med. 2001; 135:686-693.
25. Brach, JS, Vanswearingen, JM, Newman, AB, et al. Identifying early decline of physical function in community-dwelling older women: performance-based and self-report measures. Phys Ther. 2002; 82:320-328.
26. Guralnik, JM, Ferrucci, L, Simonsick, EM, et al. Lower-extremity function in persons over the age of 70 years as a predictor of subsequent disability. N Engl J Med. 1995; 332:556-561.
27. *2008 Physical Activity Guidelines for Americans*. 2008. U.S. Department of Health and Human Services.
28. Minns Lowe, CJ, Barker, KL, Dewey, M, et al. Effectiveness of physiotherapy exercise after knee arthroplasty for osteoarthritis: systematic review and meta-analysis of randomised controlled trials. BMJ. 2007; 335:812.
29. Fritz, JM, Hunter, SJ, Tracy, DM, et al. Utilization and clinical outcomes of outpatient physical therapy for medicare beneficiaries with musculoskeletal conditions. Phys Ther. 2011; 91:330-345.
30. DeJong, G, Tian, W, Smout, RJ, et al. Long-term outcomes of joint replacement rehabilitation patients discharged from skilled nursing and inpatient rehabilitation facilities. Arch Phys Med Rehabil. 2009; 90:1306-1316.
31. DeJong, G, Tian, W, Smout, RJ, et al. Use of rehabilitation and other health care services by patients with joint replacement after discharge from skilled nursing and inpatient rehabilitation facilities. Arch Phys Med Rehabil. 2009; 90:1297-1305.
32. Moffet, H, Collet, JP, Shapiro, SH, et al. Effectiveness of intensive rehabilitation on functional ability and quality of life after first total knee arthroplasty: A single-blind randomized controlled trial. Arch Phys Med Rehabil. 2004; 85:546-556.
33. LaStayo, PC, Meier, W, Marcus, RL, et al. Reversing muscle and mobility deficits 1 to 4 years after TKA: a pilot study. Clin Orthop Relat Res. 2009; 467:1493-1500.
34. Valtonen, A, Poyhonen, T, Sipila, S, et al. Effects of aquatic resistance training on mobility limitation and lower-limb impairments after knee replacement. Arch Phys Med Rehabil. 2010; 91:833-839.
35. Piva, SR, Gil, AB, Almeida, GJ, et al. A balance exercise program appears to improve function for patients with total knee arthroplasty: a randomized clinical trial. Phys Ther. 2010; 90:880-894. doi:ptj.20090150 [pii];10.2522/ptj.20090150 [doi].
36. Piva, SR, Almeida, GJ, and Wasko, MC. Association of physical function and physical activity in women with rheumatoid arthritis. Arthritis Care Res (Hoboken). 2010; 62:1144-1151.
37. Almeida, GJ, Wasko, MC, Jeong, K, et al. Physical activity measured by the SenseWear Armband in women with rheumatoid arthritis. Phys Ther. 2011; 91:1367-1376.
38. Parmelee PA, Thuras PD, et al. Validation of the Cumulative Illness Rating Scale in Geriatric Residential Population. JAGS. 1995; 43:130-137.
39. Fortin M, Steenbakkers K, et al. The electronic Cumulative Illness Rating Scale: a reliable and valid tool to assess multi-morbidity in primary care. Journal of Evaluation in Clinical Practice. 2011; 17:1089-1093.

40. Miller M, Towers A. A Manual of Guidelines for Scoring the Cumulative Illness Rating Scale for Geriatrics (CIRS-G). University of Pittsburgh, School of Medicine, Department of Geriatric Psychiatry, Western Psychiatric Institute and Clinic. 1991.
41. Linn, BS, Linn, MW, and Gurel, L. Cumulative illness rating scale. J Am Geriatr Soc. 1968; 16:622-626.
42. Bellamy, N. WOMAC Osteoarthritis Index User Guide. Version V. Brisbane, Australia 2002.
43. Bellamy, N, Buchanan, WW, Goldsmith, CH, et al. Validation study of WOMAC: a health status instrument for measuring clinically important patient relevant outcomes to antirheumatic drug therapy in patients with osteoarthritis of the hip or knee. J Rheumatol. 1988; 15:1833-1840.
44. Bellamy, N, Kean, WF, Buchanan, WW, et al. Double blind randomized controlled trial of sodium meclofenamate (Meclomen) and diclofenac sodium (Voltaren): post validation reapplication of the WOMAC Osteoarthritis Index. J Rheumatol. 1992; 19:153-159.
45. Hawker, G, Melfi, C, Paul, J, et al. Comparison of a generic (SF-36) and a disease specific (WOMAC) (Western Ontario and McMaster Universities Osteoarthritis Index) instrument in the measurement of outcomes after knee replacement surgery. J Rheumatol. 1995; 22:1193-1196.
46. Stewart, AL, Verboncoeur, CJ, McLellan, BY, et al. Physical activity outcomes of CHAMPS II: a physical activity promotion program for older adults. J Gerontol A Biol Sci Med Sci. 2001; 56:M465-M470.
47. Lopez-Olivo, MA, Landon, GC, et al. Psychosocial determinants of outcomes in knee replacement. Ann Rheum Dis. 2011; 70:1775-1781.
48. Lorig, K, Chastain, RL, Ung, E, et al. Development and evaluation of a scale to measure perceived self-efficacy in people with arthritis. Arthritis Rheum. 1989; 32:37-44.
49. Hornyak, V, Brach, JS, et al. What is the relation between fear of falling and physical activity in older adults? ACRM. 2013; 94:2529-34.
50. Andresen, EM, Malmgren, JA, Carter, WB, et al. Screening for depression in well older adults: evaluation of a short form of the CES-D (Center for Epidemiologic Studies Depression Scale). Am J Prev Med. 1994; 10:77-84.
51. Scopaz, KA, Piva, SR, Wisniewski, S, et al. Relationships of fear, anxiety, and depression with physical function in patients with knee osteoarthritis. Arch Phys Med Rehabil. 2009; 90:1866-1873.
52. Beck AT, Epstein N, Brown G, et al. An inventory for measuring clinical anxiety: psychometric properties. J Consult Clin Psych. 1988; 56:893-897.
53. Riddle, DL, Wade, JB, Jiranek, WA, et al. Preoperative pain catastrophizing predicts pain outcome after knee arthroplasty. Clin Orthop Relat Res. 2010; 468:798-806.
54. Riddle, DL, Wade, JB, and Jiranek, WA. Major depression, generalized anxiety disorder, and panic disorder in patients scheduled for knee arthroplasty. J Arthroplasty. 2010; 25:581-588.
55. Woby, SR, Watson, PJ, Roach, NK, et al. Adjustment to chronic low back pain--the relative influence of fear-avoidance beliefs, catastrophizing, and appraisals of control. Behav Res Ther. 2004; 42:761-774.
56. Fitzgerald, GK, White, DK, and Piva, SR. Associations for change in physical and psychological factors and treatment response following exercise in knee osteoarthritis: an exploratory study. Arthritis Care Res (Hoboken). 2012; 64:1673-1680.
57. Swartzman, LC, Gwadry, FG, Shapiro, AP, et al. The factor structure of the Coping Strategies Questionnaire. Pain. 1994; 57:311-316.

58. Geisser, ME, Robinson, ME, and Henson, CD. The Coping Strategies Questionnaire and chronic pain adjustment: a conceptual and empirical reanalysis. Clin J Pain. 1994; 10:98-106. Reenen, Mv, Oppe. EQ-5D-3L User Guide Basic information on how to use the EQ-5D-3L instrument. Version 5.1. 2015.
59. Zhao W, Hill MD, Palesch Y. Minimal sufficient balance--a new strategy to balance baseline covariates and preserve randomness of treatment allocation. StatMethods MedRes. 2012. doi:0962280212436447 [pii];10.1177/0962280212436447 [doi].
60. Dobson, F, Bennell, KL, et al. Recommended performance-based tests to assess physical function in people diagnosed with hip or knee osteoarthritis. Osteoarthritis Research Society International (OARSI). University of Melbourne, Australia. 2013.
61. Piva, SR, Fitzgerald, GK, Irrgang, JJ, et al. Get up and go test in patients with knee osteoarthritis. Arch Phys Med Rehabil. 2004; 85:284-289.
62. Almeida, GJ, Schroeder, CA, Gil, AB, et al. Interrater reliability and validity of the stair ascend/descend test in subjects with total knee arthroplasty. Arch Phys Med Rehabil. 2010; 91:932-938.
63. de Brito, LB, Ricardo, DR, de Araujo, DS, et al. Ability to sit and rise from the floor as a predictor of all-cause mortality. Eur J Prev Cardiol. 2012.
64. Piva, SR, Teixeira, PE, Almeida, GJ, et al. Contribution of hip abductor strength to physical function in patients with total knee arthroplasty. Phys Ther. 2011; 91:225-233.
65. Mizner, RL, Petterson, SC, Stevens, JE, et al. Preoperative quadriceps strength predicts functional ability one year after total knee arthroplasty. J Rheumatol. 2005; 32:1533-1539.
66. Hochberg, Y. A sharper Bonferroni procedure for multiple tests of significance. Biometrika 75, 800-802. 1988.o P, Roth PL, and Buster MA. The Usefulness of Unit Weights in Creating Composite Scores: A Literature Review, Application to Content Validity, and Meta-Analysis. Organizational Research Methods. 2007; 10:689-709.
67. Zhao W. Mass weighted urn design - A new randomization algorithm for unequal allocations. Contemp Clin Trials. 2015;43:209-16. doi:10.1016/j.cct.2015.06.008.
68. Nagelkerke, N, Fidler, V, Bernsen, R, et al. Estimating treatment effects in randomized clinical trials in the presence of non-compliance. Stat Med. 2000; 19:1849-1864.
69. Lombard, DN, Lombard, TN, and Winett, RA. Walking to meet health guidelines: the effect of prompting frequency and prompt structure. Health Psychol. 1995; 14:164-170.
70. Martens, EP, Pestman, WR, de, BA, et al. Instrumental variables: application and limitations. Epidemiology. 2006; 17:260-267.
71. Maracy, M and Dunn, G. Estimating dose-response effects in psychological treatment trials: the role of instrumental variables. Stat Methods Med Res. 2011; 20:191-215.
72. Kennedy, DM, Stratford, PW, Wessel, J, et al. Assessing stability and change of four performance measures: a longitudinal study evaluating outcome following total hip and knee arthroplasty. BMC Musculoskelet Disord. 2005; 6:3.
73. Rejeski, WJ, Ettinger, WH, Jr., Schumaker, S, et al. Assessing performance-related disability in patients with knee osteoarthritis. Osteoarthritis Cartilage. 1995; 3:157-167.
74. Friedman LM, Furberg CD, and DeMets DL. Fundamentals of Clinical Trials. 4th ed. New York, NY: Springer Science, 2010.

<u>APPENDIX</u> *(documents from appendix are available upon request)*

A. **Study Forms**
B. **Study Forms Coded**
C. **Scoring**
D. **Informed Consent**
E. **Study Advertisement**

Summary of changes in study protocol:

- To include the Jewish Community Center (JCC) – South Hills and the Monroeville Senior Citizen Center to the community centers providing exercise classes to the community-based group exercise.
- To include detailed description of the tests being performed on the clinical examination.
- To clarify rating in the Cumulative Illness Rating Scale form.

A Comparison of Treatment Methods for Patients Following Total Knee Replacement

Statistical Analysis Plan
(SAP)

Author: Sara R. Piva, PT, PhD
Charity G. Moore, PhD, MSHP

Date: 01/31/2018

CONTENTS

1 INTRODUCTION

2 DATA SOURCE

All case report forms and administrative forms (e.g., patient screening log, enrollment, delegation of responsibilities) will be tested for accuracy and appropriate sequence of data collection across follow-up time points. When all study forms are ready we will configure them electronically for a paperless data entry system that will be used by this study. All questionnaires and patient self-report instruments will be converted into electronic versions that will be completed on tablet computer, eliminating the need for double data entry. Data sources for the study are observed by the research staff such as clinical exam and performance-based testing and self-reported by the participants. No data are sourced from laboratory, imaging, or medical records.

Data Management will be overseen by the PI, Dr. Moore and Dr. Gil and will be coordinated by the Center for Research on Health Care Data Center (DC) at the University of Pittsburgh. The DC will create an electronic System for Data Management (eSYSDM) for data collection, tracking, follow-up, reporting, and analysis needs. The research coordinator will obtain the patient profile (name, age, gender) once a patient is recruited and deemed eligible for the study to initiate inclusion in the tracking system. The tracking system will monitor enrollment and track follow-up rates and the data entry process, providing up to date status reports. The eSYSDM includes verifying the data, out of range data checks, and repeated evaluation of data process, eliminating the possibility of most incorrect entries and preventing extensive recoding and cleaning by the statistician. To ensure data quality and integrity we will use standard methods of data collection and recording specified in the MOP, have formal staff workshop on research integrity at the beginning of the study and with new hires, document computer operations and data editing procedures, and have regular meetings with project staff to review any changes in procedure. The electronic forms will be maintained by the DC on a local network in a relational database. The DC will perform routine data edit checks for consistency. Once edited, temporary files will be merged to generate final files for data analysis. All files will be backed-up daily and archived weekly.

For all reporting and analysis, the systems analyst at the DC will export the eSYSDM data to an Access database with tables for each of the case report forms. Dr. Moore and the statisticians on the project will use the Access database for regular quality monitoring, Data and Safety Monitoring reports, and final statistical analyses of the primary and secondary manuscripts.

3 ANALYSIS OBJECTIVES

This study will be a pragmatic comparative effectiveness study, designed as a 3-group randomized clinical trial. The main goal is to provide evidence to inform the choice of exercise programs during the later stages after TKR. The three comparison groups will be:

1. Clinic-based individual outpatient rehabilitative exercise
2. Community-based group exercise classes
3. Usual medical care

The aims are to compare the outcomes of physical function and physical activity between the 3 treatment groups, to identify baseline predictors of functional recovery for both exercise groups, and to determine attrition, adherence, adverse events and co-interventions across treatment groups.

Comparison of physical function and physical activity between the 3 treatment groups will be conducted using linear mixed models with contrasts for continuous measures and chi-square tests for dichotomous measures. Logistic regression will be used to identify baseline predictors of functional response. Chi-square analyses and ANOVA will be used to compare attrition, adherence, adverse events, and co-interventions across the 3 treatment groups.

4 ANALYSIS SETS/ POPULATIONS/SUBGROUPS

Subjects will be **included** in the study if they underwent a unilateral TKR 2 to 4 months prior to study, are older than 60 years of age, experience functional limitation in daily activities (score in the WOMAC-PF of at least 9 points), s*peak English sufficient to understand study instructions, a*re willing to be randomized to one of the 3 treatment groups, and have medical clearance to participate in the study. Subjects will be **excluded** if

they:

- Have absolute or relative contraindications to exercise testing as established by the American College of Cardiology/American Heart Association;[99,100]
- Have a history of uncontrolled cardiovascular disease or hypertension;
- Are unable to walk 50 meters without an assistive device and to comfortably bear weight on the surgical knee;
- Have a history of muscular disease (e.g., muscular dystrophy) or neurological disorder that may affect lower extremity function (e.g., CVA, neuropathy, Parkinson's disease, multiple sclerosis);
- Regularly participate in structured exercise;
- Have a terminal illness;
- Are planning to have another joint replacement during the next 12 months; Do not plan to be around during the next 12 months (e.g., plan to travel, relocate to another city, etc).

The analysis population will include all participants randomized to 1 of the 3 intervention groups. All analyses will follow intention to treat.

Subgroup analyses will be conducted to test for heterogeneity of treatment effects (see section 7.2 below).

5 ENDPOINTS AND COVARIATES

Physical Function

• For patient-reported function we will use the Western Ontario and McMaster Universities Osteoarthritis Index Physical Function Subscale (WOMAC-PF). The score at 3 months will be the primary end point. The WOMAC-PF consists of 17 items related to physical function. Each item is scored on a 5-point Likert-type Scale with descriptors from 0-4 (none, mild, moderate, severe, and extreme difficulty). Scores of each item are summed for a maximum total score on the WOMAC-PF of 68. Higher scores indicate worse functional limitations. Reliability and validity of this instrument have been established.

• For performance-based physical function we will measure a battery of 6 tests easily performed in the clinical setting: (1) Self-selected gait speed-measured in m/sec while patients walk at their regular pace over 4 meters. (2) Chair rise- seated in a chair

(18"height) without armrests with arms crossed over the chest. Patients are timed during 5 repetitions of rising to a full upright position and sitting back down in the chair without assistance. (3) Single leg stance test – we will record the time of balancing on one leg while keeping the hands on the hips. The test lasts up to 60 sec and is stopped if the swing leg touches the floor, support foot moves on the floor, or arms swing away from the hips. (4) Stair ascend/descend test- patients will be timed while climbing up and down a set of 11 stairs (30 cm depth, 17 cm height) using a handrail on the preferred side. (5) Six min walk test-patients are instructed to cover as much distance as possible during 6 min with the opportunity to stop and rest if required. The test is conducted on an unobstructed, rectangular circuit (marked in meters). (6) Sitting-rising test- it scores the ability of patients to sit and rise from the floor. Assistive devices will not be permitted during these tests.

**Table 1- Time points and outcome measures of the study**	Baseline	Month 3 and 6	Call Month 1.5 & 4.5
**Aim 1**			
Physical Function (WOMAC-PF & performance tests)<br>Physical Activity (SWA & CHAMPS)	x	x	
**Aim 2**			
Demographics and Biomedical	x		
Medication	x		
Comorbidities	x		
Psychosocial (fear, anxiety, self-efficacy, depression, coping)	x	x	
Physical Impairments (pain, range of motion, muscle strength)	x	x	
**Exploratory Aim**			
Adverse Events		x	x
Attrition		x	
Adherence with intervention	monitored each visit		
Co-Interventions		x	x

Physical Activity-

- Real-time physical activity will be measured by the SenseWear Minifly (SWM)(Body Media Inc., Pittsburgh PA). Subjects will be instructed to wear the SWM on the back of the right arm during 24 hours/7 days (except during shower or water activities).
- Self-reported physical activity will be assessed using the Community Healthy Activities Model Program for Seniors questionnaire (CHAMPS). The CHAMPS is a reliable, valid, and responsive instrument. It assesses activities such as hobbies, work- and social-related activities, walking, swimming, dancing; and will complement the information obtained from the SenseWear technology.

Covariates: For the primary aim comparing the 3 intervention groups, only variables included in the randomization will be controlled in the models. This includes age, sex, body mass index, baseline physical function (measured with the WOMAC physical function subscale) and range of motion.

For the aim of predicting response, variables at the baseline that we believe may be potential predictors of treatment response, in addition to physical function include:

- Demographics and Biomedical Characteristics such as age, gender, race, education, BMI, self-rated health (excellent, good, fair, poor, or bad), discharge placement, number of prior rehabilitation sessions, TKR technique, and surgeon experience will be collected.

- Medication prescribed and over-the-counter used for pain will be recorded in the medication form. We will record the current and highest dosage of any pain medication used during the last month.

- Comorbidity data will be gathered using the Cumulative Illness Rating Scale. Subjects will be asked to indicate conditions with which they have been formally diagnosed by a physician in the past.

- Psychosocial Measures will include factors that our and other groups have demonstrated to be associated with physical function in knee OA and include: 1) Fear; 2) Anxiety; 3) Self-Efficacy; 4) Depression; 5) Coping. We hypothesize that patients with higher fear, anxiety, depression and catastrophizing coping along with low self-efficacy who participated in the community-based group exercise classes will be able to improve physical function, whereas the ones who participated in the individual outpatient exercise will not. Fear-avoidance beliefs will be measured by the Tampa Scale for Kinesiophobia. Anxiety will be measured using the Beck Anxiety Index. Self-efficacy will be measured by the Arthritis Self Efficacy Scale. Depression will be assessed by the Center for Epidemiologic Studies Short Depression Scale. Coping will the measured by the Coping Strategy Questionnaire.

- Knee Impairments – We hypothesize that patients with increased knee pain, decreased knee range of motion, and lower extremity muscle strength who participated in the outpatient rehabilitation will be able to improve physical function whereas the ones who

participated in the community-based group exercise classes will not. Knee pain in the surgical and non-surgical knee will also be measured using an 11-point pain scale. Knee range of motion will be measured by a standard goniometer. We will measure the strength of the muscle groups that have been related to the outcome of TKR[45,79] including knee extension strength and hip abduction strength measured using an isokinetic dynamometer (Biodex System 4 Pro, Shirley, NY) as we described before.

Adverse Events- We will capture information on (1) changes in knee pain, swelling, and stiffness; (2) difficulty to bear weight on the surgical leg; (3) falls; (4) if subject has been hospitalized or disabled (> ½ day in bed or required to cut back on routine activities), and; (5) if subject had a TKR on the other knee. Falls will be defined as unintentionally coming to rest at a lower position. In this study serious adverse events (SAEs) are defined as hospitalization, death, or permanent disabilty. Adverse Events (AEs) are defined as exercise-related discomforts that remains for 3 days or longer (e.g., muscle and joint soreness/pain), minor injuries (e.g., strains, sprains), and non-injurious falls. Transient side effects are defined as complaints of increased pain, stiffness, or muscle weakness for periods of 2 days or shorter. Adverse events will be captured using the CTCAE classification system version 4.02. We will compare the measures of harm such as adverse events and attrition by treatment interventions.

We will also monitor attrition, adherence and co-interventions:

- <u>Attrition</u> is defined in this study as the number of patients dropping out of the study in each group.
- <u>Adherence</u> to intervention will be estimated by the proportion of sessions attended in each group and the proportion of patients missing each session. Adherence to the individualized outpatient exercise will be recorded by the physical therapist. Adherence to the group exercise classes will be obtained from reports generated by the community centers.
- <u>Co-interventions</u> will be queried during every assessment. Some subjects could decide to seek additional treatment options while enrolled in the study. We will ask about additional treatment sought (e.g., seeking specialized care), and participation in exercises besides the ones prescribed by the study. If the subject participated in additional exercises,

we will record the frequency, duration, and type of the exercise and will analyze these data for their potential effect on the primary outcome.

6 HANDLING OF MISSING VALUES AND OTHER DATA CONVENTIONS

In this study we estimate the attrition to be 10% at the 3 months follow-up. We also estimate the attrition to be 15% at 6 months, based on our pilot work and other studies with similar populations and timeframes. We will compare baseline characteristics between patients with and without the assessment at 3 and 6 months to assess potential biases in the complete case analysis. We will also try to obtain reasons for study drop out to assess the missing data mechanism (missing completely at random, missing at random, non-ignorable missingness). If the missingness is >15%, we will use several missing data methods for imputing data and re-analyze using intention to treat (as randomized) to assess the impact of missing data on our conclusions as recommended.[97] We will first use multiple imputation (with M=10 imputations) which assumes the data are missing at random. Since the data could be missing not at random, we will use another approach of assigning the lowest observed scores for missing values differentially by treatment group (non-ignorable missingness). The approach assumes the missingness is directly related to the value of the missing data, i.e., the people who are missing data on function have worse function scores (did not come in for assessment because function was worse). Results of all approaches to missing data will be presented in the primary paper for our study. If our significance and interpretation of our treatment effect vary depending on the method of imputation, we will view any conclusions cautiously.

7 STATISTICAL METHODOLOGY

7.1 STATISTICAL PROCEDURES

General descriptive statistics: We will evaluate the statistical properties of baseline and follow-up outcome measures, including potential outliers, normality and missing data. Measures of central tendency (means, medians, other percentiles) and dispersion (standard deviations, ranges) will be computed for continuous variables, whereas frequency distributions will be calculated for categorical data. Distributions of baseline characteristics will be compared between groups to assess effectiveness of

randomization. Data transformations may be applied if needed and guided by clinical meaningfulness.

Hypothesis 1.1 *- Subjects in Groups 1 and 2 will demonstrate better physical function as compared to Group 3.*

The primary outcome of this aim is the WOMAC-PF at 3 months. This analysis will use an intention-to-treat approach. We will analyze this aim using contrasts from linear mixed models analysis for 3 and 6 month function controlling for baseline function and the randomization covariates (age, gender, BMI, physical function, ROM). We will first explore the intervention by time interaction, and then proceed to a main effects model with only group and time. Our primary interest is the 3 month comparison between the clinic-based individual outpatient exercise and the community-based exercise groups. The linear mixed models allow us to maximize the number of individuals used for the analyses as a person can contribute information at both time points, or just one time point. This analysis is advantageous to conducting a simple baseline adjusted ANCOVA at 3 months because persons missing 3 month data would not be included. The linear mixed model "borrows" information pertaining to the relationship between the 3 and 6 month outcomes such that persons missing either (but not both) can still be used in the analyses. To test if the improvements in outcomes are sustained, we will use contrasts from the linear mixed model at 6 months. For missing 3 and 6 month outcomes see missing data section.

For the battery of performance-based tests we will perform the analyses as described above, one for each measure. For these secondary outcomes we will use Hochberg's step-up procedure to control the experiment-wise Type I error rate (α=0.05),[92] which otherwise would be inflated due to the multiple endpoints. Hochberg's procedure is more powerful than Bonferroni adjustment and performs well when the number of endpoints is small and correlated with small to mid-size correlations. For this outcome we will also perform analysis using a composite score formed with unit-weighted z scores of constituent tests to provide a more stable measure of the subjects' underlying functional performance.

Hypothesis 1.2*- Subjects in Groups 1 and 2 will demonstrate increased physical activity as compared to Group 3.*

Adjusted analysis for the outcome of physical activity will parallel the analyses described

above for performance-based physical function. The outcomes of this hypothesis are real-time physical activity data captured by the SWM and self-reported physical activity from the CHAMPS questionnaire

Hypothesis 2- *A group of baseline biomedical and psychosocial measures will associate with treatment response.*

Each subject will be classified as a responder or non-responder based on a minimum change score of 20% in BOTH the WOMAC-PF and the composite score of functional performance at 3 months, thus yielding a binary outcome. Baseline variables will be summarized separately for responders and non-responders. Unadjusted odds ratios will be estimated using univariate logistic regression. To consolidate potential predictors, we will test for collinearity among baseline variables that are associated with response. Baseline measures associated with response at the $p<0.15$ level in unadjusted models will be added to multivariable logistic regression models to assess predictors of treatment response. We will limit the number of predictors going into any one model to no more than one predictor per 10 responses (or 10 non-responses, whichever is less); if more variables are significant, the model will be limited to the most significant variables, after adjusting for those deemed a-priori to be clinically significant. We expect different predictors for each group. For example, patients with worse physical function and impairments (e.g., limited range of motion and muscle weakness) will do better with individual outpatient exercise whereas those with heightened psychosocial factors (e.g., anxiety and depressive symptoms) will respond better to group exercise in the community.

Exploratory Hypothesis- *Adherence and co-interventions will be similar in all groups. The attrition rate and adverse events- mainly number of falls- will be lower in Groups 1 and 2 compared to Group 3.*

We will calculate dropout rates as proportions of subjects randomized and as a cumulative probability of remaining in the study using survival analysis techniques such as the product-limit estimator. This statistics can be estimated at various times following randomization and take into account when dropouts occur. Descriptive statistics will be used for reporting and evaluating implementation of the exercise protocols including the proportion in attendance for each session and the average number of sessions attended by

group. The two intervention groups require the same number of exercise sessions (2 per week) for 3 months. To assess the impact of non-adherence, we could conduct a per-protocol analysis for the treatment effect but this would likely result in a biased estimate of the treatment effect due to selection bias of those who are more likely to adhere not representing a true random sample of trial participants. We propose to explore using instrumental variable methodology to estimate the efficacy for our interventions in the presence of non-adherence. Instrumental variables have been mainly used in the econometrics but have been proposed as a useful tool in estimating dose-response effects in psychological treatments where participants are expected to attend multiple sessions as part of the intervention protocol. We propose to use the two-stage IV methods which can be easily implemented in Stata software using simple linear structural models for the effect of sessions attended on the primary outcome of function. We will also calculate the 6-month incidence (and 95% CI) of individual adverse events by organ system and relatedness to the study for each group. We will estimate the incidence of adverse events with specific focus on those deemed definitely, probably, or possibly related to interventions. For adverse events, clinical judgments will be considered more important than statistical testing.

7.2 MEASURES TO ADJUST FOR MULTIPLICITY, CONFOUNDERS, HETEROGENEITY, ETC.

For the battery of performance-based tests we will perform the analyses as described above, one for each measure. For these secondary outcomes we will use Hochberg's step-up procedure to control the experiment-wise Type I error rate (α=0.05),[92] which otherwise would be inflated due to the multiple endpoints. Hochberg's procedure is more powerful than Bonferroni adjustment and performs well when the number of endpoints is small and correlated with small to mid-size correlations.

Heterogeneity of Treatment Effects (HTE): Responses to our proposed intervention may vary across individuals with some having an intended benefit and others having no response, and perhaps even some patients having a negative response. We will explore HTE using several potential moderators of treatment response measured prior to randomization that may either potentiate or attenuate the effects of our intervention (e.g.,

patient gender, age, BMI, range of knee motion). These are the same prognostic variables used for the adaptive randomization in the study. These measures have been selected due to their strong associations with the study outcomes of physical function and activity. Age will be categorized into two groups ≤74 and >74; BMI will be categorized as ≤29 and >29, and knee flexion range of motion will be categorized into limited (≤94 degrees) and less limited (>94 degrees). We will also examine race and psychosocial status (e.g., anxiety and depressive symptoms) as potential effect modifiers.

As recommended by the PCORI Methodology Report, we will formally explore HTE by examining interactions between the treatment and modifier being considered. For example, we hypothesize the treatment effect would be greater in younger (≤74 years old) compared to older (>74 years old) patients due to their better health; therefore, we would test for the treatment*age interaction. Even if the interaction was not statistically significant, we would estimate the treatment effects stratified by age along with the 95% confidence intervals to look for consistency of treatment effects.

8 SENSITIVITY ANALYSES

No sensitivity analyses are planned with the exception of missing data analysis under different assumptions.

9 RATIONALE FOR ANY DEVIATION FROM PRE-SPECIFIED ANALYSIS PLAN

For the group comparisons for the primary and secondary continuous measures, linear mixed models were used with baseline in the outcome vector and unstructured covariance matrix for repeated measures within person. Fixed effects were time (baseline, 3 months, 6 months) and group*time. The main intervention effects were tested using the group*time interaction and time specific contrasts. All analyses controlled for age, gender, BMI, physical function, ROM (randomization stratification variables).

We used the combined unit weighted combined z score as the primary performance based measure. The 6 performance based measures were compared individually with no adjustment for multiplicity using Hochberg’s method as these are supplementary and not used individually for inference pertaining to performance based measures.

For dropout, we made did not use survival analysis methods for comparisons due to the very few dropouts. Dropouts were compared with percentages.

We did not conduct different missing data analysis for group comparisons because the attrition was so low (total 7.5%). In addition, we used linear mixed models which assumes data are missing at random and performs as well as multiple imputation. We did not conduct instrumental variable analyses because the adherence to each program (physical therapy and community exercise) was very high.

For the primary analysis, we added a comparison of the response rates across the three groups using three definitions of response based on external guidance. The three definitions of response:

1) 20% improvement in both the WOMAC-PF and at least 3/6 tests of performance. (originally planned response outcome)
2) 50% improvement in WOMAC-PF, 20% improvement in at least 2/6 tests of performance, and a rate of at least “somewhat better” in patient global assessment of change in health status
3) A rate of at least “moderately better” in patient global assessments of change in health status.

Analyses looking at baseline predictors or response will be submitted in a separate manuscript from the primary analysis.
