# Supplementary material for: Effectiveness of Later-Stage Exercise Programs vs Usual Medical Care on Physical Function and Activity After Total Knee Replacement: A Randomized Clinical Trial
Source: JAMA Netw Open. 2019 Feb 22;2(2):e190018. doi: 10.1001/jamanetworkopen.2019.0018 (PMC6484600; doi:10.1001/jamanetworkopen.2019.0018)
Supplement: Supplement 2. — eFigure. Graphs of Individual Performance-Based Tests Over Time eTable 1. Performance-Based Tests Over Time in Study Arms eTable 2. Baseline Values of Variables Tested as Potential Moderators eTable 3. Mean Values of Physical Function Measured by WOMAC-PF and Number of Participants at Each Time Point for the Levels of Potential Moderators eTable 4. Test of Heterogeneity of Treatment Effects at Each Time Point [file jamanetwopen-2-e190018-s002.pdf]

Supplementary Online Content

Piva SR, Schneider MJ, Moore CG, et al. Effectiveness of later-stage exercise programs vs usual medical care on physical function and activity after total knee replacement: a randomized clinical trial. *JAMA Netw Open*. 2019;2(2):e190018. doi:10.1001/jamanetworkopen.2019.0018

eFigure. Graphs of Individual Performance-Based Tests Over Time

eTable 1. Performance-Based Tests Over Time in Study Arms

eTable 2. Baseline Values of Variables Tested as Potential Moderators

eTable 3. Mean Values of Physical Function Measured by WOMAC-PF and Number of Participants at Each Time Point for the Levels of Potential Moderators

eTable 4. Test of Heterogeneity of Treatment Effects at Each Time Point

This supplementary material has been provided by the authors to give readers additional information about their work.

© 2019 Piva SR et al. *JAMA Network Open*.

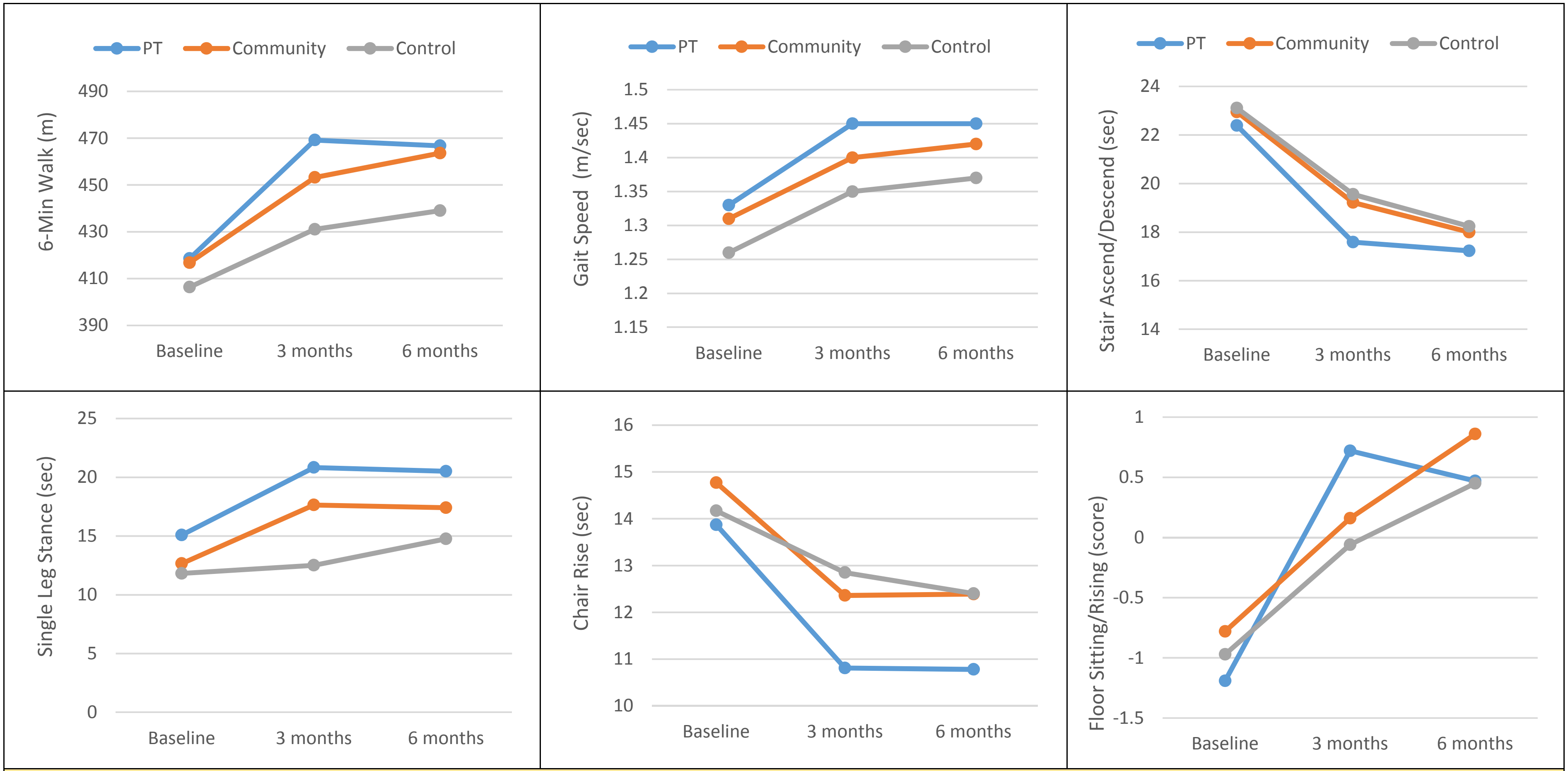

eFigure. Graphs of Individual Performance-Based Tests Over Time

Abbreviation: PT, Physical Therapy.

Data are the means for each performance-based test for baseline, 3 months, and 6 months. 6 minute walk test assesses the distance covered while walking during 6 min, gait speed is calculated during walking over 40-meters, stair test measures the time to go up and down a set of 11 stairs, single leg stance test records the time balancing on one leg while keeping the hands on the hips, chair rise test measures time during 5 repetitions of rising to a full upright position and sitting back down in the chair, and sitting/rising test assesses the ability of participants to sit and rise from the floor.

© 2019 Piva SR et al. *JAMA Network Open*.

eTable 1. Performance-Based Tests Over Time in Study Arms

	Baseline N	Baseline Mean (SD)	3 months N	3 months Mean (SD)	6 months N	6 months Mean (SD)	Δ Ba to 3-mo	Δ%	Δ Ba to 6-mo	Δ%
Six-minute Walk Test (meters)										
Physical Therapy	96	419 (97)	88	469 (100)	83	467 (116)	50	12	48	12
Community	96	417 (105)	86	453 (104)	76	464 (120)	36	9	47	11
Control	48	406 (91)	43	431 (82)	40	439 (98)	25	6	33	8
Gait Speed (meters/sec)										
Physical Therapy	96	1.33 (0.27)	89	1.45 (0.28)	83	1.45 (0.28)	0.12	9	0.12	9
Community	96	1.31 (0.28)	86	1.40 (0.29)	76	1.42 (0.31)	0.09	7	0.11	8
Control	48	1.26 (0.27)	44	1.35 (0.25)	40	1.37 (0.27)	0.09	7	0.11	9
Stair Ascend/Descend Test (sec)										
Physical Therapy	96	22.4 (10.8)	89	17.6 (7.0)	83	17.2 (6.9)	-4.8	-21	-5.2	-23
Community	96	23.0 (10.8)	85	19.2 (8.5)	75	18.0 (8.4)	-3.8	-17	-5	-22
Control	48	23.1 (9.2)	44	19.6 (9.7)	40	18.2 (8.4)	-3.5	-15	-4.9	-21
Single Leg Stance Test (sec)										
Physical Therapy	96	15.1 (17.5)	89	20.8 (19.5)	83	20.5 (19.4)	5.7	38	5.4	36
Community	96	12.7 (14.2)	87	17.6 (18.8)	76	17.4 (17.0)	4.9	39	4.7	37
Control	48	11.8 (12.8)	44	12.5 (13.4)	40	14.8 (16.9)	0.7	6	3	25
Chair Rise Test (sec)										
Physical Therapy	95	13.9 (5.4)	89	10.8 (3.0)	83	10.8 (2.9)	-3.1	-22	-3.1	-22
Community	94	14.8 (5.2)	85	12.4 (3.6)	75	12.4 (5.4)	-2.4	-16	-2.4	-16
Control	46	14.2 (4.1)	43	12.9 (3.2)	39	12.4 (4.1)	-1.3	-9	-1.8	-13
Siting/Rising Test Score[a]										
Physical Therapy	75	-1.2 (4.0)	72	0.72 (3.5)	69	0.5 (5.6)	1.92	160	1.7	142
Community	69	-0.78 (4.0)	67	0.16 (4.3)	60	0.86 (4.2)	0.94	121	1.64	210
Control	36	-0.97 (3.9)	34	-0.06 (4.7)	31	0.45 (4.1)	0.91	94	1.42	146

Abbreviations: Ba, Baseline; mo, months; Δ, change; Δ%, percentage change
a Negative scores in sitting/rising test represents worse physical function

© 2019 Piva SR et al. *JAMA Network Open*.

eTable 2. Baseline Values of Variables Tested as Potential Moderators

**Variables** [a]	**Physical Therapy (n=96)**	**Community (n=96)**	**Control (n=48)**
Age – yr	69 ± 6	70 ± 7	70 ± 7
Female – no. (%)	59 (62)	58 (60)	31 (65)
Body-mass index (kg/m^2)	30.8 ± 5.3	31.1 ± 6.3	31.5 ± 5.1
Knee flexion surgical knee (degrees)	124 ± 11	123 ± 11	124 ± 10
Knee extension surgical knee (degrees)	-1.2 ± 5.8	-0.7 ± 4.4	-2.1 ± 5.2
Knee flexion non-surgical knee (degrees)	131 ± 12	130 ± 13	131 ± 11
Knee extension non-surgical knee (degrees)	1.6 ± 5.1	1.5 ± 4.5	0.7 ± 4.9
Depression [b]	8.8 ± 3.0	8.0 ± 2.8	7.6 ± 2.1
Anxiety [c]	4.0 ± 4.3	3.3 ± 3.3	2.8 ± 3.0
Fear of movement [d]	34.0 ± 5.1	33.6 ± 6.4	33.7 ± 5.8
Self-Efficacy pain [e]	76.8 ± 15.4	75.1 ± 16.0	74.1 ± 18.8
Self-efficacy function	83.3 ± 13.2	82.6 ± 12.5	82.4 ± 14.1
Self-efficacy symptom	82.9 ± 12.4	80.9 ± 14.7	79.1 ± 16.4

a Data presented as mean ± SD, unless otherwise specified.
b Depressive symptom assessed by the Center for Epidemiologic Studies Short Depression Scale.[30]
c Anxiety measured using the Beck Anxiety Index.[31]
d Fear of movement measured by the Tampa Scale for Kinesiophobia.[66]
e Self-efficacy measured by the Arthritis Self Efficacy Scale.[33]

© 2019 Piva SR et al. *JAMA Network Open*.

eTable 3. Mean Values of Physical Function Measured by WOMAC-PF and Number of Participants at Each Time Point for the Levels of Potential Moderators

Potential Moderators	Time Point[a]	Physical Therapy		Community		Control	
		n	Mean	n	Mean	n	Mean
Age ≤74	0	76	21.09	73	20.26	34	20.88
	3	71	10.11	65	11.62	31	12.68
	6	70	9.51	66	10.68	31	10.97
Age >74	0	20	20.05	23	21	14	18.57
	3	19	10.26	22	14.05	13	9.92
	6	19	10.63	22	11.14	14	13.57
Male	0	37	19.19	38	18.92	17	18.47
	3	34	10.68	34	10.97	16	10.75
	6	34	9.53	34	9.09	17	11
Female	0	59	21.93	58	21.43	31	21.16
	3	56	9.82	53	13.04	28	12.5
	6	55	9.89	54	11.87	28	12.25
BMI<30	0	46	18.02	46	20	20	19.1
	3	45	8.4	42	9.69	19	10.53
	6	44	6.73	43	8.58	19	12.11
BMI≥30	0	50	23.5	50	20.84	28	21
	3	45	11.89	45	14.6	25	12.88
	6	45	12.71	45	12.91	26	11.54
WOMAC-PF < 20	0	46	14.22	47	14.55	26	14.23
	3	43	7.37	42	10.21	24	8.58
	6	43	6.86	43	8.37	24	9.17
WOMAC-PF ≥ 20	0	50	27	49	26.08	22	27.27
	3	47	12.68	45	14.11	20	15.8
	6	46	12.46	45	13.11	21	14.76
Surgical knee flexion ≤120	0	30	25.57	37	22.03	19	20.58
	3	27	13.59	34	14.47	19	12.05
	6	29	14.55	33	13.58	19	11.11
Surgical knee flexion >120	0	66	18.74	59	19.44	29	19.97
	3	63	8.67	53	10.79	25	11.72
	6	60	7.43	55	9.13	26	12.27

© 2019 Piva SR et al. *JAMA Network Open*.

Surgical knee extension <0 degrees	0	56	21.77	54	20.37	35	20.34
	3	52	11.75	51	13	33	12.33
	6	51	10.63	51	10.65	34	12.79
Surgical knee extension ≥0 degrees	0	40	19.63	42	20.52	13	19.85
	3	38	7.95	36	11.14	11	10.45
	6	38	8.58	37	11	11	8.64
Non-surgical knee flexion ≤120	0	20	25.2	22	23.23	8	21.75
	3	17	13.12	20	15	8	15.63
	6	18	12.83	20	15.05	8	12.13
Non-surgical knee flexion >120	0	76	19.74	74	19.61	40	19.9
	3	73	9.45	67	11.4	36	11.03
	6	71	8.97	68	9.54	37	11.7
Non-surgical knee extension <0 degrees	0	35	22.09	33	22.06	22	22.59
	3	33	13.7	32	15.72	21	13.81
	6	32	11.91	32	12.53	21	13.24
Non-surgical knee extension ≥0 degrees	0	61	20.18	63	19.59	26	18.19
	3	57	8.09	55	10.2	23	10.09
	6	57	8.54	56	9.8	24	10.5
No anxiety or depression [b]	0	58	19.81	67	19.67	34	20.06
	3	54	9.57	60	10.2	32	12.41
	6	53	9.08	63	9.03	32	11.88
Anxiety and/or depression	0	38	22.5	29	22.21	14	20.57
	3	36	11	27	16.74	12	10.42
	6	36	10.75	25	15.24	13	11.54
Low fear of movement [c]	0	41	19.93	47	20.26	25	19.44
	3	38	8.74	43	11.7	24	10.04
	6	40	8.43	44	9.73	24	10.17
High fear of movement	0	55	21.58	49	20.61	23	21.04
	3	52	11.17	44	12.75	20	14.05
	6	49	10.84	44	11.86	21	13.62
Low pain self-efficacy [d]	0	46	21.87	44	21.64	25	22.08
	3	44	11.5	41	14	21	11.9
	6	42	9.98	41	12.76	22	14
High pain self-efficacy	0	50	19.96	52	19.42	23	18.17
	3	46	8.85	46	10.65	23	11.83

© 2019 Piva SR et al. *JAMA Network Open*.

	6	47	9.55	47	9.09	23	9.65
Low function self-efficacy	0	42	23.93	49	23.08	27	22.93
	3	41	12.12	43	15.37	24	14.92
	6	38	12.92	45	14.58	25	14.12
High function self-efficacy	0	54	18.5	47	17.68	21	16.71
	3	49	8.49	44	9.16	20	8.2
	6	51	7.39	43	6.84	20	8.85
Low symptom self-efficacy	0	41	22.54	45	20.67	22	21.27
	3	41	11.44	41	14.17	20	10.2
	6	38	10.16	42	12.86	21	12.1
High symptom self-efficacy	0	55	19.64	51	20.24	26	19.31
	3	49	9.06	46	10.5	24	13.25
	6	51	9.45	46	8.91	24	11.5

Abbreviation: WOMAC-PF, Western Ontario and McMaster Universities Osteoarthritis Index Physical-function Scale.
a Time points 0, 3, and 6 correspond to baseline, 3 and 6 months follow-ups.
b Anxiety assessed by Beck Anxiety Index (anxiety defined by values ≥ 8).[31] Depressive symptom assessed by the Center for Epidemiologic Studies Short Depression Scale (depression defined by values ≥ 10).[30] Due to low prevalence, individuals were classified as having either anxiety, depression, or both.
c Fear of movement measured by the Tampa Scale for Kinesiophobia (medians were used as cut-offs).[66]
d Self-efficacy measured by the Arthritis Self Efficacy Scale (medians were used as cut-offs).[33]

© 2019 Piva SR et al. *JAMA Network Open*.

eTable 4. Test of Heterogeneity of Treatment Effects at Each Time Point

Potential Moderators	Time Point [a]	Physical Therapy vs Control	Community vs Control	Physical Therapy vs Community
		Mean Difference (95% CI) in WOMAC-PF		
Age ≤74	3	-2.10 (-4.75; 0.56)	-0.20 (-2.89; 2.49)	-1.90 (-4.00; 0.21)
Age >74		-1.87 (-6.29; 2.55)	1.09 (-3.21; 5.38)	-2.95 (-6.82; 0.92)
Age ≤74	6	-1.17 (-3.89; 1.55)	0.61 (-2.14; 3.36)	-1.78 (-3.93; 0.37)
Age >74		-3.86 (-8.32; 0.60)	-4.13 (-8.46; 0.20)	0.27 (-3.69; 4.23)
Male	3	-1.17 (-4.86; 2.52)	-0.54 (-4.23; 3.16)	-0.64 9-3.60; 2.32)
Female		-2.68 (-5.52; 0.15)	0.46 (-2.40; 3.31)	-3.14 (-5.47; -0.80)
Male	6	-1.27 (-5.04; 2.50)	-1.61 (-5.38; 2.17)	0.34 (-2.72; 3.40)
Female		-2.66 (-5.60; 0.28)	-0.33 (-3.28; 2.62)	-2.33 (-4.75; 0.09)
BMI<30	3	-1.51 (-4.88; 1.86)	-0.82 (-4.22; 2.58)	-0.69 (-3.33; 1.94)
BMI≥30		-2.73 (-5.77; 0.32)	0.97 (-2.09; 4.02)	-3.69 (-6.27; -1.12)
BMI<30	6	**-4.71* (-8.17; -1.25)**	**-3.47* (-6.94; 0.01**	-1.24 (-3.94; 1.45)
BMI≥30		**-0.02 (-3.12; 3.07)**	**1.30 (-1.80; 4.40)**	-1.32 (-3.96; 1.32)
WOMAC-PF < 20	3	-0.85 (-4.02; 2.32)	1.50 (-1.68; 4.69)	-2.35 (-5.04; 0.33)
WOMAC-PF ≥ 20		-3.80 (-7.10; -0.49)	-1.48 (-4.80; 1.85)	-2.32 (-4.91; 0.26)
WOMAC-PF < 20	6	-1.93 (-5.22; 1.37)	-0.96 (-4.25; 2.34)	-0.97 (-3.76; 1.81)
WOMAC-PF ≥ 20		-2.61 (-6.01; 0.79)	-0.83 (-4.25; 2.59)	-1.78 (-4.48; 0.91)
Surgical knee flexion ≤120	3	-0.61 (-4.27; 3.05)	2.22 (-1.29; 5.74)	-2.83 9-5.97; 0.30)
Surgical knee flexion >120		-3.12 (-6.01; -0.22)	-1.34 (-4.31; 1.63)	-1.77 (-4.06; 0.51)
Surgical knee flexion ≤120	6	**1.34* (-2.35; 5.02)**	**2.39* (-1.20; 5.98**	-1.05 (-4.21; 2.10)
Surgical knee flexion >120		**-4.17 (-7.11; -1.24)**	**-2.95 (-5.93; 0.03)**	-1.22 (-3.55; 1.10)
Surgical knee extension <0 degree	3	-1.49 (-4.21; 1.22)	0.32 (-2.41; 3.05)	-1.81 (-4.22; 0.59)
Surgical knee extension ≥0 degree		-2.44 (-6.64; 1.77)	0.25 (-3.97; 4.47)	-2.69 (-5.53; 0.16)
Surgical knee extension <0 degree	6	-2.88 (-5.69; -0.07)	-1.73 (-4.54; 1.08)	-1.15 (-3.65; 1.35)
Surgical knee extension ≥0 degree		0.12 (-4.24; 4.48)	1.68 (-2.69; 6.04)	-1.56 (-4.49; 1.38)
Non-surgical knee flexion≤120	3	-0.86 (-6.24; 4.52)	3.06 (-2.24; 8.36)	-3.92 (-8.01; 0.17)
Non-surgical knee flexion>120		-3.29 (-8.57; 1.99)	-0.51 (-5.68; 4.66)	-2.78 (-6.82; 1.25)
Non-surgical knee flexion ≤120	6	-1.83 (-4.34; 0.68)	0.20 (-2.35; 2.74)	-2.03 (-4.11; 0.06)
Non-surgical knee flexion>120		-2.40 (-4.97; 0.17)	-1.82 (-4.41; 0.77)	-0.58 (-2.72; 1.56)
Non-surgical knee extension <0 degrees	3	-0.02 (-3.36; 3.31)	2.34 (-1.02; 5.70)	-2.36 (-5.32; 0.59)
Non-surgical knee extension ≥0 degrees		-3.24 (-6.19; -0.29)	-1.13 (-4.09; 1.82)	-2.11 (-4.36; 0.15)

© 2019 Piva SR et al. *JAMA Network Open*.

Non-surgical knee extension <0 degrees	6	-1.45 (-5.02; 2.11)	-0.30 (-3.88; 3.27)	-1.15 (-4.31; 2.02)
Non-surgical knee extension ≥0 degrees		-2.53 (-5.64; 0.58)	-1.07 (-4.18; 2.04)	-1.46 (-3.86; 0.93)
No anxiety or depression [b]	3	-2.39 (-5.04; 0.26)	**-1.94** (-4.54; 0.66)**	**-0.45** (-2.66; 1.77)**
Anxiety and/or depression		-1.18 (-5.11; 2.75)	**4.84 (0.74; 8.93)**	**-6.01 (-9.02; -3.00)**
No anxiety or depression	6	-2.69 (-5.44; 0.05)	**-2.78** (-5.45; -[illegible]**	**0.09* (-2.19; 2.37)**
Anxiety and/or depression		-0.96 (-4.92; 3.01)	**3.89 (-0.28; 8.07)**	**-4.85 (-8.01; -1.69)**
Low fear of movement [c]	3	-1.34 (-4.52; 1.84)	1.82 (-1.29; 4.93)	-3.16 (-5.86; -0.46)
High fear of movement		-3.37 (-6.58; -0.17)	-1.91 (-5.19; 1.38)	-1.47 (-3.97; 1.03)
Low fear of movement	6	-1.96 (-5.21; 1.29)	-0.62 (-3.81; 2.58)	-1.34 (-4.08; 1.40)
High fear of movement		-2.64 (-5.93; 0.64)	-1.19 (-4.53; 2.15)	-1.45 (-4.05; 1.14)
Low pain self-efficacy [d]	3	-0.93 (-4.16; 2.31)	1.67 (-1.61; 4.94)	-2.59 (-5.25; 0.06)
High pain self-efficacy		-3.13 (-6.26; -0.01)	-1.24 (-4.36; 1.89)	-1.90 (-4.43; 0.64)
Low pain self-efficacy	6	-3.86 (-7.16; -0.56)	-0.98 (-4.30; 2.34)	-2.87 (-5.62; -0.13)
High pain self-efficacy		-0.46 (-3.65; 2.74	-0.53 (-3.73; 2.66)	0.08 (-2.50; 2.66)
Low function self-efficacy	3	**-4.04* (-7.12; -0.96)**	-0.21 (-3.26; 2.84)	-3.83 (-6.45; -1.21)
High function self-efficacy		**0.43 (-2.76; 3.62)**	0.93 (-2.32; 4.17)	-0.50 (-2.98; 1.99)
Low function self-efficacy	6	-2.12 (-5.27; 1.03)	0.12 (-2.95; 3.19)	-2.24 (-4.93; 0.45)
High function self-efficacy		-1.39 (-4.63; 1.86)	-1.50 (-4.82; 1.83)	0.11 (-2.42; 2.64)
Low symptom self-efficacy	3	0.11 (-3.18; 3.41)	**3.16* (-0.12; 6.44)**	-3.05 (-5.72; -0.38)
High symptom self-efficacy		-3.88 (-6.89; -0.87)	**-2.57 (-5.62; 0.48)**	-1.31 (-3.78; 1.17)
Low symptom self-efficacy	6	-2.18 (-5.59; 1.24)	0.83 (-2.52; 4.19)	-3.01 (-5.81; -0.21)
High symptom self-efficacy		-2.07 (-5.18; 1.04)	-2.36 (-5.53; 0.80)	0.29 (-2.26; 2.85)

Abbreviation: WOMAC-PF, Western Ontario and McMaster Universities Osteoarthritis Index Physical-function Scale.
a Time points 3 and 6 correspond to 3 and 6 months follow-ups.
b Anxiety assessed by Beck Anxiety Index (anxiety defined by values ≥ 8). Depressive symptom assessed by the Center for Epidemiologic Studies Short Depression Scale (depression defined by values ≥ 10). Due to low prevalence, individuals were classified as having either anxiety, depression, or both.
c Fear of movement measured by the Tampa Scale for Kinesiophobia (medians were used as cut-offs).66
d Self-efficacy measured by the Arthritis Self Efficacy Scale (medians were used as cut-offs).33
* p-values < 0.05 for the subgroup-treatment effect interaction.
** p-values < 0.01 for the subgroup-treatment effect interaction.
Bold values denote mean difference and 95% CI for significant subgroup-treatment effect interaction.

© 2019 Piva SR et al. *JAMA Network Open*.
