# Supplementary material for: Effectiveness of Later-Stage Exercise Programs vs Usual Medical Care on Physical Function and Activity After Total Knee Replacement: A Randomized Clinical Trial
Source: JAMA Netw Open. 2019 Feb 22;2(2):e190018. doi: 10.1001/jamanetworkopen.2019.0018 (PMC6484600; doi:10.1001/jamanetworkopen.2019.0018)
Supplement: Supplement 3. — Data Sharing Statement [file jamanetwopen-2-e190018-s003.pdf]

Data Sharing Statement

Piva. Effectiveness of Later-Stage Exercise Programs vs Usual Medical Care on Physical Function and Activity After Total Knee Replacement. *JAMA Netw Open*. Published February 22, 2019. 10.1001/jamanetworkopen.2019.0018

Data
Data available: Yes
Data types: Deidentified participant data, Data dictionary
How to access data: The final dataset of this study will include de-identified information on demographics, health status, results of physical examinations, questionnaires, and outcomes measures. When this study is completed, the original, the de-identified dataset along with the code books and documented programming code used for creating the final study population, for creating variables, and for conducting all outcomes analyses will be made available to researchers upon written request under a data-sharing agreement that provides for: a) a commitment to use the data only for research purposes and not to identify any individual participant; b) a commitment to securing the data using appropriate computer technology; and c) a commitment to destroying or returning the data after analyses have been completed. These data will be ready after publication of the manuscripts with the results of specific aims. Communicate with the corresponding author to request the data.
When available: With publication

Supporting Documents
Document types: Statistical/analytic code
How to access documents: The protocol was submitted as Supplemental 1.
When available: With publication

Additional Information
Who can access the data: Researchers whose proposed use of the data has been approved.
Types of analyses: For any purpose.
Mechanisms of data availability: With a data access agreement.
